# Supplementary material for: Is it feasible to implement a community-based participatory group programme to address issues of access to healthcare for people with disabilities in Luuka district Uganda? A study protocol for a mixed-methods pilot study
Source: BMJ Open. 2023 Sep 28;13(9):e074217. doi: 10.1136/bmjopen-2023-074217 (PMC10546107; doi:10.1136/bmjopen-2023-074217)
Supplement: Supplementary data [file bmjopen-2023-074217supp001.pdf]

Supplement 1: MRC/UVRI & LSHTM Uganda Research Unit **and LSHTM**

Questionnaire: Adult version

Table of contents

Section 100: Respondent and household characteristics
Section 200: Washington Group Short Set Enhanced
Section 300: Quality of life
Section 400: Health condition and nutrition
Section 500: Health care utilization
Section 600: Participation
Section 700: Attitudes
Section 800: PLA-D specific questions

Nr.		
001	LOCATION ID NUMBER	________________
002	**ID NUMBER**	________________
003	**INTERVIEW TYPE**	1=Person with disabilities (direct)<br>2=Proxy for person with disabilities (unable to communicate/understand)<br>2=Proxy for person with disabilities (child<18)<br>________________
004	**Interviewer 1 initials and code**	
005	**Date of interview**	__ __ 2023<br>(DAY MONTH)
006	**Time start interview**	__ hrs __ min
007	**Time end interview**	__ hrs __ min

SECTION 100 - RESPONDENT AND HOUSEHOLD CHARACTERISTICS (WOPS)

I am going to start by asking you some questions about yourself and your living situation.

101	SEX	1= Male<br>2= Female
102	How old are you now?	Years……..
103	In what year were you born?<br>**IF KNOWN INCLUDE DATE OF BIRTH; CORRECT INCONSISTENCIES WITH AGE**	Year of birth….
		Date of birth<br>…../……/……….
104	What is current marital status?	1= Single/ Never married<br>2= Married / Cohabitating<br>3= Divorced / Separated<br>4= Widowed
105	What is / was your occupation?<br>Any other?<br>**TICK ALL THAT APPLY**	House work
		Farming
		Fishing
		Labouring (e.g. clearing streets, weeding)
		Tailoring
		Teacher / nurse
		Government administration
		Shop attendant
		other,
105a	Other spouse occupation SPECIFY	______________________
106	What is / was your spouse's occupation? Any other?<br>**TICK ALL THAT APPLY**	House work
		Farming
		Fishing
		Labouring(e.g. clearing streets, weeding etc)
		Tailoring
		Teacher / nurse
		Government administration
		Shop attendant
		Other
106a	Other respondent occupation SPECIFY	___________________
107	Are you still working?	1= Yes, full-time<br>1a= Yes, part-time<br>1b= Yes, self-employed

		1c= No, retired<br>2= No
108	What is your highest level of education?	1= No formal education<br>2= Less than primary (incomplete) school<br>3= Completed primary school<br>4= complete O level<br>5= Completed A level<br>6= Higher education than secondary school (diploma course or BTVET)<br>7= Adult education only<br>8= College/ University or more<br>88= Don't know
109	What is the total number of people who live in your household?	[ ][ ]<br>Persons

LIVING CONDITIONS

110	What is the predominant material used for roofing?	1= Thatch<br>2= Tile<br>3= Iron sheets<br>4= Mixed iron/tile<br>5= Other
111	What is the state of the roof?	1= Very Good<br>1= Good<br>2= Moderate<br>3= Bad<br>4= Very bad
112	What are the walls of the house made of?	1= Burned brick<br>2= Unburned brick<br>3= Mud
113	What is the house floor made of?	1= Mud<br>2= Cement<br>3= Tiles
114	Who owns the house?	1= Self<br>2= Spouse<br>3= Respondent's children<br>4= Someone else in the household<br>5= Renting (landlord)<br>87= Other

Kuper H, *et al. BMJ Open* 2023; 13:e074217. doi: 10.1136/bmjopen-2023-074217

SECTION 200: Washington Group Enhanced Set

**201**	Do you have difficulty seeing even when wearing your glasses/contact lenses? Would you say….	1= No difficulty<br>2= Some difficulty<br>3= A lot of difficulty<br>4= Cannot do at all
**202**	Do you have difficulty hearing, even when using a hearing aid(s)? Would you say…	1= No difficulty<br>2= Some difficulty<br>3= A lot of difficulty<br>4= Cannot do at all
**203**	Do you have difficulty walking or climbing steps? Would you say…	1= No difficulty<br>2= Some difficulty<br>3= A lot of difficulty<br>4= Cannot do at all
**204**	Using your usual languate do you have difficulty communicating, for example, understanding or behing understood? Would you say…	1= No difficulty<br>2= Some difficulty<br>3= A lot of difficulty<br>4= Cannot do at all
**205**	Do you have difficulty remembering or concentrating? Would you say…	1= No difficulty<br>2= Some difficulty<br>3= A lot of difficulty<br>4= Cannot do at all
**206**	Do you have difficulty with self care, such as washing all over or dressing? Would you say…	1= No difficulty<br>2= Some difficulty<br>3= A lot of difficulty<br>4= Cannot do at all
**207**	Do you have difficulty raising a 2 liter bottle of water or soda from the waist to eye level? Would you say…	1= No difficulty<br>2= Some difficulty<br>3= A lot of difficulty<br>4= Cannot do at all
**208**	Do you have difficulty using your hands and fingers, such as picking up small objects, for example, a button or pencil, or opening or closing containers or bottles? Would you say…	1= No difficulty<br>2= Some difficulty<br>3= A lot of difficulty<br>4= Cannot do at all
**209**	How often do you feel worried, nervous or anxious? Would you say…	1= Daily<br>2= Weekly<br>3= Monthly<br>4= A few times a year<br>5= Never
	Thinking about the last time you felt worried, nervous or anxious, how would you describe the level of these feelings. Would you say…	1= A little<br>2= A lot<br>3= Somewhere between a little and a lot
**209**	How often do you feel depressed? Would you say…	1= Daily<br>2= Weekly<br>3= Monthly<br>4= A few times a year

		5= Never
**210**	Thinking about the last time you felt depressed, how would you describe the level of these feelings. Would you say…	1= A little<br>2= A lot<br>3= Somewhere between a little and a lot
**211**	At what age did you first start experiencing these difficulties	____ years<br>0 if age<1<br>999 if don't know

SECTION 300 QUALITY OF LIFE (WHO-QOL BREF)

This assessment asks how you feel about your quality of life, health, or other areas of your life. Please answer all the questions. If you are unsure about which response to give to a question, please choose the one that appears most appropriate. This can often be your first response. Please keep in mind your standards, hopes, pleasures and concerns. We ask that you think about your life in the last two weeks.

301	How would you rate your quality of life?	1= Very poor<br>2= Poor<br>3= Neither poor nor good<br>4= Good<br>5= Very good
302	How satisfied are you with your health?	1= Very dissatisfied<br>2= Disatisfied<br>3= Neither satisfied nor dissatisfied<br>4= Satisfied<br>5= Very satisfied
303	To what extent do you feel that physical pain prevents you from doing what you need to do?	1= Not at all<br>2= A little<br>3= A moderate amount<br>4= Very much<br>5= An extreme amount
304	How much do you need any medical treatment to function in your daily life?	1= Not at all<br>2= A little<br>3= A moderate amount<br>4= Very much<br>5= An extreme amount
305	How much do you enjoy life?	1= Not at all<br>2= A little<br>3= A moderate amount<br>4= Very much<br>5= An extreme amount
306	To what extent do you feel your life to be meaningful?	1= Not at all<br>2= A little<br>3= A moderate amount<br>4= Very much<br>5= An extreme amount

307	How well are you able to concentrate?	1= Not at all<br>2= A little<br>3= A moderate amount<br>4= Very much<br>5= Extremely
308	How safe do you feel in your daily life?	1= Not at all<br>2= A little<br>3= A moderate amount<br>4= Very much<br>5= Extremely
309	How healthy is your physical environment?	1= Not at all<br>2= A little<br>3= A moderate amount<br>4= Very much<br>5= Extremely
310	Do you have enough energy for your everday activities?	1= Not at all<br>2= A little<br>3= Moderately<br>4= Mostly<br>5= Completely
311	Are you able to accept your bodily appearance?	1= Not at all<br>2= A little<br>3= Moderately<br>4= Mostly<br>5= Completely
312	Have you enough money to meet your needs?	1= Not at all<br>2= A little<br>3= Moderately<br>4= Mostly<br>5= Completely
313	How available to you is the information that you need in your day-to-day life?	1= Not at all<br>2= A little<br>3= Moderately<br>4= Mostly<br>5= Completely
314	To what extent do you have the opportunity for leisure activities?	1= Not at all<br>2= A little<br>3= Moderately<br>4= Mostly<br>5= Completely

315	How well are you able to get around?	1=Very poor<br>2= Poor<br>3= Neither poor nor good<br>4= Good<br>5= Very good
316	How satisfied are you with your sleep?	1= Very dissatisfied<br>2= Disatisfied<br>3= Neither satisfied nor dissatisfied<br>4= Satisfied<br>5= Very satisfied
317	How satisfied are you with your ability to perform your daily activities?	1= Very dissatisfied<br>2= Disatisfied<br>3= Neither satisfied nor dissatisfied<br>4= Satisfied<br>5= Very satisfied
318	How satisfied are you with your capacity for work?	1= Very dissatisfied<br>2= Disatisfied<br>3= Neither satisfied nor dissatisfied<br>4= Satisfied<br>5= Very satisfied
319	How satisfied are you with yourself?	1= Very dissatisfied<br>2= Disatisfied<br>3= Neither satisfied nor dissatisfied<br>4= Satisfied<br>5= Very satisfied
320	How satisfied are you with your personal relationships?	1= Very dissatisfied<br>2= Disatisfied<br>3= Neither satisfied nor dissatisfied<br>4= Satisfied<br>5= Very satisfied
321	How satisfied are you with your sex life?	1= Very dissatisfied<br>2= Disatisfied<br>3= Neither satisfied nor dissatisfied<br>4= Satisfied<br>5= Very satisfied
322	How satisfied are you with the support you get from your friends?	1= Very dissatisfied<br>2= Disatisfied<br>3= Neither satisfied nor dissatisfied<br>4= Satisfied<br>5= Very satisfied

323	How satisfied are you with the conditions of your living place?	1= Very dissatisfied<br>2= Disatisfied<br>3= Neither satisfied nor dissatisfied<br>4= Satisfied<br>5= Very satisfied
324	How satisfied are you with your access to health services?	1= Very dissatisfied<br>2= Disatisfied<br>3= Neither satisfied nor dissatisfied<br>4= Satisfied<br>5= Very satisfied
325	How satisfied are you with your transport?	1= Very dissatisfied<br>2= Disatisfied<br>3= Neither satisfied nor dissatisfied<br>4= Satisfied<br>5= Very satisfied
326	How often do you have negative feelings such as blue mood, depair, anxiety or depression?	1= Never<br>2= Infrequently<br>3= Sometimes<br>4= Frequently<br>5= Always

SECTION 400: HEALTH CONDITIONS AND NUTRITION
I am now going to ask some questions about your health

Self-rated health

401	In general, how would you rate your health?	1= Good<br>2= Rather good<br>3= Poor<br>4= Very poor

Nutrition (WOPS)

402	How many servings of fruit do you eat on a typical day?	Servings
403	How many servings of vegetables do you eat on a typical day?	Servings
404	In the last 12 months, were you ever hungry, but didn't eat because you couldn't afford enough food?	1 = Yes<br>2 = No
405	In the last 12 months, how often did you eat less than you felt you should because there wasn't enough food?	1= every month<br>2= almost every month<br>3= some months. But not every month<br>4= only in 1 or 2 months<br>5= Never

HEALTH STATUS (Model disability survey)

	**Have you been told by a doctor or health professional that you have…**		**If yes, have you been given medicine or treatment in the last 6-12 months?**
**406**	Vision loss	1 = Yes<br>2 = No	
**407**	Hearing loss	1 = Yes<br>2 = No	
**408**	High blood pressure	1 = Yes<br>2 = No	
**409**	Diabetes	1 = Yes<br>2 = No	
**410**	Arthritis	1 = Yes<br>2 = No	
**411**	Heart disease, coronary disease, heart attack	1 = Yes<br>2 = No	
**412**	Chronic bronchitis or emphysema	1 = Yes	

		2 = No	
**413**	Asthma, allergic, respiratory disease	1 = Yes<br>2 = No	
**414**	Back pain or disc problems	1 = Yes<br>2 = No	
**415**	Migraine (recurrent headaches)	1 = Yes<br>2 = No	
**416**	Stroke	1 = Yes<br>2 = No	
**417**	Depression or anxiety	1 = Yes<br>2 = No	
**418**	Gastritis or ulcer	1 = Yes<br>2 = No	
**419**	Tumour or cancer	1 = Yes<br>2 = No	
**420**	Trauma	1 = Yes<br>2 = No	
**421**	Dementia	1 = Yes<br>2 = No	
**422**	Kidney disease	1 = Yes<br>2 = No	
**423**	Skin disease	1 = Yes<br>2 = No	
**424**	Tuberculosis	1 = Yes<br>2 = No	
**425**	Mental (psychiatric) or behavioural disorders	1 = Yes<br>2 = No	

Section 500: Health care utilization

I would now like to know about your recent experiences with obtaining health care from health care workers, hospitals, clinics and the health care system. I want to know if you needed health care recently, and if so, why you needed health care provider youreceived care from.

501	In the last 2 years did you need health care?	1=Yes<br>2=No
502	The last time you needed health care, did you get health care?<br>**If "yes" Skip to 504**	1= Yes<br>2= No
**Interviewer: if respondent did not get health care, Q 502= "NO", ask Q 503A, 503a,503b and 503I then Skip to question 511**		
503A	**Interviewer: Respondent can select ONLY one <u>main reason</u> for visit. Use showcard**<br>**If respondent is having difficulties answering, you can read the categories**<br>What was the main reason you needed care, even if you did not get care?	
	1= Communicable diseases, infections, malaria, infection TB, HIV	
	2= Nutritional deficiencies	
	3= Acute conditions, (diarrhea, flu, headaches, fever, cough and others)	
	4= Injury (if FALLS record 4 and fill in FALLS in 403a (SPECIFY)	
	5= Surgery	
	6= Sleep problem	
	7= Occupational /work related condition/injury	
	8= Chronic pain in joints/arthritis (joints, back, neck)	
	9= Diabetes or related complications	
	10= Problems with heart including unexplained pain in chest	
	11= Problems with mouth, teeth, swallowing	
	12= Problems with breathing	
	13= High blood pressure, hypertension	
	14= Stroke/ sudden paralysis of one side of body	

	15= Generalized pain(stomach, muscle or other nonspecific pain)	
	16= Depression, anxiety	
	17= Cancer	
	87= Other	
503a	Other main reason SPECIFY	______________________
503b	Which reason(s) best explains why you did not get health care?<br><br>**Record all the numbers that respondent mentions spontaneously.**	
	1= could not afford the cost of the visit	
	2= no transport available	
	3= could not afford the cost of the transport	
	4= you were previously badly treated	
	5= could not make time off work or had other commitments	
	6= the health care provider's drugs or equipment were inadequate	
	7= the health care provider's skills were inadequate	
	8= you did not know where to go	
	9= you tried but were denied health care	
	10= you thought you were not sick enough	
	87= other	
503I	Other best reason why not get health care SPECIFY	______________________<br>______________________
504	Thinking about health care you needed since last visit / in the last 2 years, where did you go most often when you felt sick or needed to consult someone about your health?	1= Private doctor's office
		2= Private clinic or health centre
		3= Government / public clinic or health centre
		4= Government / public hospital
		5= Charity or church run clinic

	**Interviewer: <u>Only one answer</u> allowed**	(name specific ones)
		6= Charity or church run hospital (name specific ones)
		7= Traditional healers / herbalist /shrine
		8= pharmacy
		9= Pastor or religious healer
		87= other
504a	How many visits do you make to a health facility in a year?	Number __________________
505	Did you have to pay fees for consultation and/or drugs?**If no skip to 507**	1= Yes<br>2= No
506	Who paid for the last visit?	Self
		Spouse
		Son/daughter
		Other relative
		Other not relative
		Insurance
		was free

Interviewer ask Q 507, 508,509, and 510 ONLY when respondent use health services check Q 504
If not using health service skip to 511

507	When you visit the health facility, how long do you usually have to wait before it is your turn to be seen by a nurse or doctor?	1= Not long<br>2= Quite long<br>3= Long<br>4= Very long
508	When you visit the health facility, do the health professionals usually give you the time to explain to them what your health problem is?	1= Always<br>2= Most of the time<br>3= Sometimes<br>4= Not usually<br>5= Never
509	When you visit the health facility, do the health professionals usually take the time to explain your health problem and treatment in a way that you understand?	1= Always<br>2= Most of the time<br>3= Sometimes<br>4= Not usually<br>5= Never
509a	When you visit the health facility, do the health professionals usually take time to involve you in making decisions for your treatment?[1]	1= Always<br>2= Most of the time<br>3= Sometimes<br>4= Not usually<br>5= Never
510	When you visit the health facility, are you treated respectfully?	1= Always<br>2= Most of the time<br>3= Sometimes<br>4= Not usually<br>5= Never
511	Overall, are you satisfied with the services?	1= Very satisfied<br>2= Satisfied<br>3= Neither satisfied nor dissatisfied<br>4= Dissatisfied<br>5= Very dissatisfied
512	What are the reason(s) that people will go to the traditional healer for treatment?<br>**TICK ALL THAT APPLY**	Closer distance
		Traditional healer are cheaper
		Traditional healer allow you to pay in kind/ with goods
		Traditional healer will wait for payment
		Traditional healer is more knowledgeable about this

[1] Question added from Model Disability Survey

		problem
		Traditional healers give better treatment
		Decision made by caregiver or family
512a	Have you visited a traditional healer for treatment?<br>**If no skip to 513**	1= yes<br>2= no
512b	During the last 12 months/one year, how often have you visited a traditional healer?	1 = Once or twice<br>2 = Three to six times<br>3 = More than six times<br>8 = Don't know

Health centre/clinic, hospital stays

513	In the last two years have you stayed an overnight in a health centre/ health clinic/hospital?<br>**IF NO SKIP TO 5600**	1= Yes<br>2= No
514	How many times have you been hospitalized in the last 2 years?	Number of admissions<br>______________
515	What type of hospital was it the last time you were hospitalized? Public, private or church run?	1 = Public hospital<br>2 = Private hospital<br>3 = Charity or church run hospital<br>87 = Other Specify
**516**<br>.	**RECORD REASON HOSPITALISATION**<br>**ONLY ONE REASON**<br>Which reason best describes why you were last hospitalized?<br><br>1= Communicable diseases, infections, malaria, infection TB, HIV<br>2= Nutritional deficiencies<br>3= Acute conditions, (diarrhea, flu, headaches, fever, cough and others)<br>4= Injury (if FALLS record 4 and fill in FALLS in 403a (SPECIFY)<br>5= Surgery<br>6= Sleep problem<br>7= Occupational /work related condition/injury<br>8= Chronic pain in joints/arthritis (joints, back, neck)<br>9= Diabetes or related complications<br>10= Problems with heart including unexplained pain in chest<br>11= Problems with mouth, teeth, swallowing<br>12= Problems with breathing<br>13= High blood pressure, hypertension<br>14= Stroke/ sudden paralysis of one side of body	

	15= Generalized pain(stomach, muscle or other nonspecific pain)<br>16= Depression, anxiety<br>17= Cancer<br>87= Other	
516a	Other best reason respondent was hospitalized	________________
517	Who paid for this hospitalization?	Self
		Spouse
		Son/daughter
		Other relative
		Insurance
		was free
		Other
517a	Other paid hospitalization specify	
518	What was the outcome or result of your stay in the health centre/ health clinic/hospital? Did your condition…	1= Get much better<br>2= Get better<br>3= No change<br>4= Get worse<br>5= Get much worse
519	Was this the outcome or results you had expected?	1= yes<br>2= no

SECTION 600: Participation (SINTEF)

I'm going to ask you some questions about your involvement in different aspects of family, social life and society. Please listen to each one and answer yes, no, sometimes, sometimes or not applicable.

Q. #	Question	Codes					Go to Q
		Yes	No	Sometimes	N/A	Don't know	
601	Are you consulted about making household decisions?	1	2	3	4	5	
602	Do you go with the family to events such as family gatherings, weddings, thanksgivings, funerals and birthdays, and other social functions?	1	2	3	4	5	
603	Do you feel involved and part of the household or family?	1	2	3	4	5	
604	Does the family involve you in conversations?	1	2	3	4	5	
605	Does the family help you with daily activities/tasks?	1	2	3	4	5	"No, DK, N/A" →607
606	Do you appreciate it or like the fact that you get this help?	1	2	3	4	5	
607	Do you/did you take part in your own traditional practices (e.g initiation ceremonies)?	1	2	3	4	5	
608	Are you aware of organizations for people with disabilities (DPO)?	1	2				"No" →610
609	Are you a member of a DPO?	1	2				
610	Do you participate in local community meetings?	1	2	3	4	5	"No" →612
611	Do you feel your voice is being heard?	1	2			5	
612	Did you vote in the last election?	1	2			5	**"No"→ sect 700**
613	Was it related to your disability that you didn't vote?	1	2			5	

Kuper H, *et al. BMJ Open* 2023; 13:e074217. doi: 10.1136/bmjopen-2023-074217

Section 700: Attitudes (Model Disability Survey)

Now I want to ask you some questions about the attitudes of people around you. When answering these questions please tell me on a scale from 1 to 5, where 1 is not at all and 5 means completely.

Q. #	Question	Codes					
		1 Not at all	2	3	4	5 Yes, complete ly	98 Not applicable
701	Do you have problems getting involved in society because of the attitudes of others?	1	2	3	4	5	98
702	Do you feel that some people treat you unfairly?	1	2	3	4	5	98
703	Do you make your own choices about your day-to-day life? For example, where you go, what to eat.	1	2	3	4	5	98
704	Do you feel other people accept you?	1	2	3	4	5	98
705	Do you feel that other people respect you? For example, do you feel that others value you as a person and listen to what you have to say?	1	2	3	4	5	98
706	Do you consider yourself a burden on society?	1	2	3	4	5	98
707	Do people around you tend to become impatient with you?	1	2	3	4	5	98
708	Do people around you not expect much from you?	1	2	3	4	5	98
709	Is living with dignity a problem for you because of the attitudes and actions of others?	1	2	3	4	5	98

SECTION 800: PLA-D

BASELINE only

Now I would like to ask you some questions about your needs and expectations of the programme

Nr.		CODE
801	What are the three biggest issues that in your day-to-day life that you find difficult or need support with?	
802	What are the three biggest issues related to your health you find difficult or need support with?	
803	What are your main goals for attending this group?	

END-LINE only
Now I would like to ask you some questions about your feelings about the programme?

Remind the participant of their goals from the baseline

Nr.		CODE
804	Do you feel that you have achieved or met your goals?	1=Not at all<br>2= Partially<br>3= Fully
805	Please explain	
806	How satisfied were you with the content of the group programme?	1= Very dissatisfied<br>2= Disatisfied<br>3= Neither satisfied nor dissatisfied<br>4= Satisfied<br>5= Very satisfied
807	How satisfied were you with the organization of the programme (e.g. transport, location, refreshments)?	1= Very dissatisfied<br>2= Disatisfied<br>3= Neither satisfied nor dissatisfied<br>4= Satisfied<br>5= Very satisfied
808	How satisfied were you with the facilitators of the programme?	1= Very dissatisfied<br>2= Disatisfied<br>3= Neither satisfied nor dissatisfied<br>4= Satisfied<br>5= Very satisfied
809	Can you suggest ways in which the	

	programme could be improved?	

810	**RECORD THE TIME**	___ Hrs __Min	TIMEPART2END

Closing statement and thank the respondent
INTERVIEWER: This is the end of the interview
This completes the interview. We thank you for your time and answers. I have your contact details and may be in touch again.
