# Supplementary material for: Is it feasible to implement a community-based participatory group programme to address issues of access to healthcare for people with disabilities in Luuka district Uganda? A study protocol for a mixed-methods pilot study
Source: BMJ Open. 2023 Sep 28;13(9):e074217. doi: 10.1136/bmjopen-2023-074217 (PMC10546107; doi:10.1136/bmjopen-2023-074217)
Supplement: Supplementary data [file bmjopen-2023-074217supp002.pdf]

MRC/UVRI & LSHTM Uganda Research Unit **and LSHTM**

Questionnaire: Child version (aged 5-17)

Table of contents

Section 100: Respondent and household characteristics
Section 200: Washington Group Short Set Enhanced
Section 300: Quality of life
Section 400: Health condition and nutrition
Section 500: Health care utilization
Section 600: Participation
Section 700: Attitudes
Section 800: PLA-D specific questions

Nr.		
001	LOCATION ID NUMBER	________________
002	**ID NUMBER**	________________
003	**INTERVIEW TYPE**	1=Person with disabilities (direct)<br>2=Proxy for person with disabilities (unable to communicate/understand)<br>2=Proxy for person with disabilities (child<18)<br>________________
004	**Interviewer 1 initials and code**	
005	**Date of interview**	__ __ 2023<br>(DAY MONTH)
006	**Time start interview**	__ hrs __ min
007	**Time end interview**	__ hrs __ min

SECTION 100 - RESPONDENT AND HOUSEHOLD CHARACTERISTICS (WOPS)

I am going to start by asking you some questions about your child and his/her living situation.

101	SEX	1= Male<br>2= Female
102	How old is [NAME] now?	Years……..
103	In what year was [NAME] born?<br>**IF KNOWN INCLUDE DATE OF BIRTH; CORRECT INCONSISTENCIES WITH AGE**	Year of birth….
		Date of birth<br>…../……/………..
104	Has [NAME] ever attended school or any early childhood education programme	1=Yes<br>2=No
105	What is the highest grade of school [NAME] has ever attended?	1=Early childhood education<br>2=Primary<br>3=Lower secondary<br>4=Upper secondary<br>5=Higher
106	At any time during the current school year did [NAME] attend school of any early childhood education programme?	1=Yes<br>2=No
107	During this current school year, what grade of school is [NAME] attending?	1=Early childhood education<br>2=Primary<br>3=Lower secondary<br>4=Upper secondary<br>5=Higher
108	What is the occupation of your head of household? Any other?<br>**TICK ALL THAT APPLY**	House work
		Farming
		Fishing
		Labouring (e.g. clearing streets, weeding)
		Tailoring
		Teacher / nurse
		Government administration
		Shop attendant
		other,
109	What is head of	1= No formal education

	household's highest level of education?	2= Less than primary (incomplete) school<br>3= Completed primary school<br>4= complete O level<br>5= Completed A level<br>6= Higher education than secondary school (diploma course or BTVET)<br>7= Adult education only<br>8= College/ University or more<br>88= Don't know
110	What is the total number of people who live in your household?	☐☐<br>Persons

LIVING CONDITIONS

111	What is the predominant material used for roofing?	1= Thatch<br>2= Tile<br>3= Iron sheets<br>4= Mixed iron/tile<br>5= Other
112	What is the state of the roof?	1= Very Good<br>1= Good<br>2= Moderate<br>3= Bad<br>4= Very bad
113	What are the walls of the house made of?	1= Burned brick<br>2= Unburned brick<br>3= Mud
114	What is the house floor made of?	1= Mud<br>2= Cement<br>3= Tiles
115	Who owns the house?	1= Self<br>2= Spouse<br>3= Respondent's children<br>4= Someone else in the household<br>5= Renting (landlord)<br>87= Other

SECTION 200: Washington Group Child Functioning Set
I would like to ask you some questions about difficulties [NAME] may have

201	Does [NAME] wear glasses or contact lenses?	1=Yes<br>2=No	No – skip to 203
202	When wearing his/her glasses, does [NAME] have difficulty seeing? Would you say… [read response categories]	1=No difficulty<br>2=Some difficulty<br>3= A lot of difficulty<br>4= Cannot do	
203	Does [NAME] have difficulty seeing? Would you say… [read response categories]	1=No difficulty<br>2=Some difficulty<br>3= A lot of difficulty<br>4= Cannot do	
204	Does [NAME] use a hearing aid?	1=Yes<br>2=No	No – skip to 206
205	When using his/her hearing aid, does [NAME] have difficulty hearing sounds like people's voices or music? Would you say… [read response categories]	1=No difficulty<br>2=Some difficulty<br>3= A lot of difficulty<br>4= Cannot do	
206	Does [NAME] have difficulty hearing sounds like people's voices or music? Would you say… [read response categories]	1=No difficulty<br>2=Some difficulty<br>3= A lot of difficulty<br>4= Cannot do	
207	Does [NAME] use any equipment or receive assistance for walking?	1=Yes<br>2=No	No – skip to 212
208	Without his/her equipment or assistance, does [NAME] have difficulty walking 100 yards/meters on level ground? That would be about the length of 1 football field. Would you say… [read response categories]	1=No difficulty<br>2=Some difficulty<br>3= A lot of difficulty<br>4= Cannot do	
209	Without (his/her) equipment or assistance, does [NAME] have difficulty walking 500 meters/yards on level ground?<br>That would be about the length of 5 football fields.	1=No difficulty<br>2=Some difficulty<br>3= A lot of difficulty<br>4= Cannot do	
210	With (his/her) equipment or assistance, does [NAME] have difficulty walking 100 meters/yards on level ground? That would be about the length of 1 football field.	1=No difficulty<br>2=Some difficulty<br>3= A lot of difficulty<br>4= Cannot do	
211	With (his/her) equipment or assistance, does [NAME] have difficulty walking 500 meters/yards on level ground?<br>That would be about the length of 5 football fields.	1=No difficulty<br>2=Some difficulty<br>3= A lot of difficulty<br>4= Cannot do	
212	Compared with children of the same age, does [NAME] have difficulty walking 100 meters/yards	1=No difficulty<br>2=Some difficulty	

	on level ground?<br>That would be about the length of 1 football field.	3= A lot of difficulty<br>4= Cannot do	
213	Compared with children of the same age, does [NAME] have difficulty walking 500 meters/yards on level ground? That would be about the length of 5 football fields.	1=No difficulty<br>2=Some difficulty<br>3= A lot of difficulty<br>4= Cannot do	
214	Does [NAME] have difficulty with self-care such as feeding or dressing (himself/herself)?	1=No difficulty<br>2=Some difficulty<br>3= A lot of difficulty<br>4= Cannot do	
215	When **[NAME]** speaks, does (he/she) have difficulty being understood by people inside of this household?	1=No difficulty<br>2=Some difficulty<br>3= A lot of difficulty<br>4= Cannot do	
216	When [NAME] speaks, does (he/she) have difficulty being understood by people outside of this household?	1=No difficulty<br>2=Some difficulty<br>3= A lot of difficulty<br>4= Cannot do	
217	Compared with children of the same age, does [NAME] have difficulty learning things?	1=No difficulty<br>2=Some difficulty<br>3= A lot of difficulty<br>4= Cannot do	
218	Compared with children of the same age, does [NAME] have difficulty remembering things?	1=No difficulty<br>2=Some difficulty<br>3= A lot of difficulty<br>4= Cannot do	
219	Does [NAME] have difficulty concentrating on an activity that (he/she) enjoys doing?	1=No difficulty<br>2=Some difficulty<br>3= A lot of difficulty<br>4= Cannot do	
220	Does [NAME] have difficulty accepting changes in (his/her) routine?	1=No difficulty<br>2=Some difficulty<br>3= A lot of difficulty<br>4= Cannot do	
221	Compared with children of the same age, does [NAME] have difficulty controlling (his/her) behaviour?	1=No difficulty<br>2=Some difficulty<br>3= A lot of difficulty<br>4= Cannot do	
222	Does [NAME] have difficulty making friends?	1=No difficulty<br>2=Some difficulty<br>3= A lot of difficulty<br>4= Cannot do	
	The next questions have different options for answers. I am going to read these to you after each question.		

223	I would like to know how often [NAME] seems very anxious, nervous or worried.<br><br>Would you say: daily, weekly, monthly, a few times a year or never?	1=Daily<br>2=Weekly<br>3=Monthly<br>4=A few times a year<br>5=Never	
224	I would also like to know how often [NAME] seems very sad or depressed.<br><br>Would you say: daily, weekly, monthly, a few times a year or never?	1=Daily<br>2=Weekly<br>3=Monthly<br>4=A few times a year<br>5=Never	

SECTION 300 QUALITY OF LIFE (KIDSCREEN-PARENT)

301	In general, how would you say your child's health is?	1=Poor<br>2=Fair<br>3=Good<br>4=Very good<br>5=Excellent
	Over the past 2 weeks….	
302	Has your child felt fit and well?	1=Not at all<br>2=Slightly<br>3=Moderately<br>4=Very<br>5=Extremely
303	Has your child felt full of energy?	1=Never<br>2=Seldom<br>3=Quite often<br>4=Very often<br>5=Always
304	Has your child felt sad?	1=Never<br>2=Seldom<br>3=Quite often<br>4=Very often<br>5=Always
305	Has your child felt lonely?	1=Never<br>2=Seldom<br>3=Quite often<br>4=Very often<br>5=Always
306	Has your child had enough time for him/herself?	1=Never<br>2=Seldom<br>3=Quite often<br>4=Very often<br>5=Always
307	Has your child been able to do the things he/she wants to do in his/her free time?	1=Never<br>2=Seldom<br>3=Quite often<br>4=Very often<br>5=Always

308	Has your child felt that his/her parent(s) treated him/her fairly?	1=Never<br>2=Seldom<br>3=Quite often<br>4=Very often<br>5=Always
309	Has your child had fun with his/her friends?	1=Never<br>2=Seldom<br>3=Quite often<br>4=Very often<br>5=Always
310	Has your child been able to pay attention?	1=Never<br>2=Seldom<br>3=Quite often<br>4=Very often<br>5=Always
311	Has your child got on well at school?	1=Not at all<br>2=Slightly<br>3=Moderately<br>4=Very<br>5=Extremely

Kuper H, *et al. BMJ Open* 2023; 13:e074217. doi: 10.1136/bmjopen-2023-074217

SECTION 400: HEALTH CONDITIONS AND NUTRITION

I am now going to ask some questions about your child's health

Nutrition (WOPS)

402	How many servings of fruit does [NAME] eat on a typical day?	Servings
403	How many servings of vegetables does [NAME]eat on a typical day?	Servings
404	In the last 12 months, did [NAME] ever hungry, but didn't eat because you couldn't afford enough food?	1 = Yes<br>2 = No
405	In the last 12 months, how often did [NAME] eat less than you felt [NAME] should because there wasn't enough food?	1= every month<br>2= almost every month<br>3= some months. But not every month<br>4= only in 1 or 2 months<br>5= Never

HEALTH STATUS (Model disability survey)

	**Has [NAME] been told by a doctor or health professional that he/she has…**		**If yes, has [NAME] been given medicine or treatment in the last 6-12 months?**
**406**	Vision loss	1 = Yes<br>2 = No	
**407**	Hearing loss	1 = Yes<br>2 = No	
**408**	High blood pressure	1 = Yes<br>2 = No	
**409**	Diabetes	1 = Yes<br>2 = No	
**410**	Arthritis	1 = Yes<br>2 = No	
**411**	Heart disease, coronary disease, heart attack	1 = Yes<br>2 = No	
**412**	Chronic bronchitis or emphysema	1 = Yes<br>2 = No	
**413**	Asthma, allergic, respiratory disease	1 = Yes<br>2 = No	
**414**	Back pain or disc problems	1 = Yes<br>2 = No	
**415**	Migraine (recurrent headaches)	1 = Yes	

		2 = No	
**416**	Stroke	1 = Yes<br>2 = No	
**417**	Depression or anxiety	1 = Yes<br>2 = No	
**418**	Gastritis or ulcer	1 = Yes<br>2 = No	
**419**	Tumour or cancer	1 = Yes<br>2 = No	
**420**	Trauma	1 = Yes<br>2 = No	
**421**	Dementia	1 = Yes<br>2 = No	
**422**	Kidney disease	1 = Yes<br>2 = No	
**423**	Skin disease	1 = Yes<br>2 = No	
**424**	Tuberculosis	1 = Yes<br>2 = No	
**425**	Mental (psychiatric) or behavioural disorders	1 = Yes<br>2 = No	

Section 500: Health care utilization

I would now like to know about your recent experiences with obtaining health care for your child from health care workers, hospitals, clinics and the health care system. I want to know if your child needed health care recently, and if so, why you needed health care provider you received care from.

501	In the last 2 years did your child need health care?	1=Yes<br>2=No
502	The last time your child needed health care, did you get health care?<br>**If "yes" Skip to 504**	1= Yes<br>2= No
**Interviewer: if respondent did not get health care, Q 502= "NO", ask Q 503A, 503a,503b and 503I then Skip to question 511**		
503A	**Interviewer: Respondent can select ONLY one <u>main reason</u> for visit. Use showcard**<br>**If respondent is having difficulties answering, you can read the categories**<br>What was the main reason your child needed care, even if your child did not get care?	
	1= Communicable diseases, infections, malaria, infection TB, HIV	
	2= Nutritional deficiencies	
	3= Acute conditions, (diarrhea, flu, headaches, fever, cough and others)	
	4= Injury (if FALLS record 4 and fill in FALLS in 403a (SPECIFY)	
	5= Surgery	
	6= Sleep problem	
	7= Occupational /work related condition/injury	
	8= Chronic pain in joints/arthritis (joints, back, neck)	
	9= Diabetes or related complications	
	10= Problems with heart including unexplained pain in chest	
	11= Problems with mouth, teeth, swallowing	
	12= Problems with breathing	
	13= High blood pressure, hypertension	

	14= Stroke/ sudden paralysis of one side of body	
	15= Generalized pain(stomach, muscle or other nonspecific pain)	
	16= Depression, anxiety	
	17= Cancer	
	87= Other	
503a	Other main reason SPECIFY	________________________
503b	Which reason(s) best explains why your child did not get health care?<br><br>**Record all the numbers that respondent mentions spontaneously.**	
	1= could not afford the cost of the visit	
	2= no transport available	
	3= could not afford the cost of the transport	
	4= your child was previously badly treated	
	5= could not make time off work or had other commitments	
	6= the health care provider's drugs or equipment were inadequate	
	7= the health care provider's skills were inadequate	
	8= you did not know where to go	
	9= you tried but were denied health care	
	10= you thought your child was not sick enough	
	87= other	
503l	Other best reason why not get health care SPECIFY	________________________<br><br>________________________
504	Thinking about health care your child needed since last visit / in the last 2 years, where did your child go most often when he/she felt sick or needed to consult someone about health?	1= Private doctor's office
		2= Private clinic or health centre
		3= Government / public clinic or health centre
		4= Government / public hospital
		5= Charity or church run clinic

	**Interviewer: <u>Only one answer</u> allowed**	(name specific ones)
		6= Charity or church run hospital (name specific ones)
		7= Traditional healers / herbalist /shrine
		8= pharmacy
		9= Pastor or religious healer
		87= other
504a	How many visits do your child make to a health facility in a year?	Number __________________
505	Did you have to pay fees for consultation and/or drugs?**If no skip to 507**	1= Yes<br>2= No
506	Who paid for the last visit?	Self (i.e. parent being interviewed)
		Spouse
		Son/daughter
		Other relative
		Other not relative
		Insurance
		was free

Interviewer ask Q 507, 508,509, and 510 ONLY when respondent use health services check Q 504
If not using health service skip to 511

507	When you visit the health facility, how long do you usually have to wait before it is your child's turn to be seen by a nurse or doctor?	1= Not long<br>2= Quite long<br>3= Long<br>4= Very long
508	When you visit the health facility, do the health professionals usually give you the time to explain to them what your child's health problem is?	1= Always<br>2= Most of the time<br>3= Sometimes<br>4= Not usually<br>5= Never
509	When you visit the health facility, do the health professionals usually take the time to explain your child's health problem and treatment in a way that you understand?	1= Always<br>2= Most of the time<br>3= Sometimes<br>4= Not usually<br>5= Never
509a	When you visit the health facility, do the health professionals usually take time to involve you/your child in making decisions for your treatment?[1]	1= Always<br>2= Most of the time<br>3= Sometimes<br>4= Not usually<br>5= Never
510	When you visit the health facility, is your child treated respectfully?	1= Always<br>2= Most of the time<br>3= Sometimes<br>4= Not usually<br>5= Never
511	Overall, are you satisfied with the services?	1= Very satisfied<br>2= Satisfied<br>3= Neither satisfied nor dissatisfied<br>4= Dissatisfied<br>5= Very dissatisfied
512	What are the reason(s) that people will go to the traditional healer for treatment?<br>**TICK ALL THAT APPLY**	Closer distance
		Traditional healer are cheaper
		Traditional healer allow you to pay in kind/ with goods
		Traditional healer will wait for payment
		Traditional healer is more knowledgeable about this

[1] Question added from Model Disability Survey

		problem
		Traditional healers give better treatment
		Decision made by caregiver or family
512a	Have you visited a traditional healer for treatment for your child?<br>**If no skip to 513**	1= yes<br>2= no
512b	During the last 12 months/one year, how often have you visited a traditional healer for treatment for your child?	1 = Once or twice<br>2 = Three to six times<br>3 = More than six times<br>8 = Don’t know

Health centre/clinic, hospital stays

513	In the last two years has your child stayed an overnight in a health centre/ health clinic/hospital?<br>**IF NO SKIP TO 5600**	1= Yes<br>2= No
514	How many times has your child been hospitalized in the last 2 years?	Number of admissions<br>______________
515	What type of hospital was it the last time your child was hospitalized? Public, private or church run?	1 = Public hospital<br>2 = Private hospital<br>3 = Charity or church run hospital<br>87 = Other Specify

**516** .	**RECORD REASON HOSPITALISATION**<br>**ONLY ONE REASON**<br>Which reason best describes why your child was last hospitalized?<br><br>1= Communicable diseases, infections, malaria, infection TB, HIV<br>2= Nutritional deficiencies<br>3= Acute conditions, (diarrhea, flu, headaches, fever, cough and others)<br>4= Injury (if FALLS record 4 and fill in FALLS in 403a (SPECIFY)<br>5= Surgery<br>6= Sleep problem<br>7= Occupational /work related condition/injury<br>8= Chronic pain in joints/arthritis (joints, back, neck)<br>9= Diabetes or related complications<br>10= Problems with heart including unexplained pain in chest<br>11= Problems with mouth, teeth, swallowing<br>12= Problems with breathing<br>13= High blood pressure, hypertension<br>14= Stroke/ sudden paralysis of one side of body<br>15= Generalized pain(stomach, muscle or other nonspecific pain)<br>16= Depression, anxiety<br>17= Cancer<br>87= Other	
516a	Other best reason child was hospitalized	______________
517	Who paid for this hospitalization?	Self<br>Spouse<br>Son/daughter<br>Other relative<br>Insurance<br>was free<br>Other
517a	Other paid hospitalization specify	
518	What was the outcome or result of your child's stay in the health centre/ health clinic/hospital? Did his/her condition…	1= Get much better<br>2= Get better<br>3= No change<br>4= Get worse<br>5= Get much worse
519	Was this the outcome or results you had expected?	1= yes<br>2= no

SECTION 600: Participation (CASP)

We are interested in finding out about the activities that your child participates in at home, school and in the community. You will be asked about your child's current level of participation with activities as compared to others their age. For each item, choose one of the following:

Full participation, your child participates in the activities the same as or more than others his/her age. [*With or without assistive devices or equipment*]

Somewhat limited, your child participates in the activities somewhat less than others his/her age. [*He/she may also need occasional supervision or assistance*]

Very limited, your child participates in the activities much less than others his/her age. [*He/she may also need a lot of supervision or assistance*]

Unable, your child cannot participate in the activities, although others his/her age do participate.

Not applicable, others your child's age would not be expected to participate in the activities.

	Compared to other children of your child's age, what is your child's current level of participation in the following activities<br><br>HOME PARTICIPATION	
601	Social, play or leisure activities with family members at home (e.g., games, hobbies, "hanging out")	1=Full participation<br>2=Somewhat limited<br>3=Very limited<br>4=Unable<br>5=Not applicable
602	Social, play or leisure activities with friends at home	1=Full participation<br>2=Somewhat limited<br>3=Very limited<br>4=Unable<br>5=Not applicable
603	Family chores, responsibilities and decisions at home (e.g., involvement in household chores and decisions about plans))	1=Full participation<br>2=Somewhat limited<br>3=Very limited<br>4=Unable<br>5=Not applicable
604	Self-care activities (e.g. eating, dressing, bathing, combing or brushing hair, using the toilet)	1=Full participation<br>2=Somewhat limited<br>3=Very limited<br>4=Unable

		5=Not applicable
605	Moving about in and around the home	1=Full participation<br>2=Somewhat limited<br>3=Very limited<br>4=Unable<br>5=Not applicable
606	Communicating with others at home	1=Full participation<br>2=Somewhat limited<br>3=Very limited<br>4=Unable<br>5=Not applicable
	NEIGHBOURHOOD & COMMUNITY PARTICIPATION	
607	Social, play, or leisure activities with friends in the neighbourhood and community (e.g., casual games, “hanging out,” going to public places like the market, or church)	1=Full participation<br>2=Somewhat limited<br>3=Very limited<br>4=Unable<br>5=Not applicable
608	Structured events and activities in the neighbourhood and community (e.g. football, church or mosque, community meetings, and family functions)	1=Full participation<br>2=Somewhat limited<br>3=Very limited<br>4=Unable<br>5=Not applicable
609	Moving around the neighbourhood and community (e.g., public buildings, taxi’s, market) [*Please consider your child’s primary way of moving around, NOT your use of transportation*]	1=Full participation<br>2=Somewhat limited<br>3=Very limited<br>4=Unable<br>5=Not applicable
610	Communicating with others in the neighbourhood and community	1=Full participation<br>2=Somewhat limited<br>3=Very limited<br>4=Unable<br>5=Not applicable
	SCHOOL PARTICIPATION	
611	Educational (academic) activities with other pupils in your child’s classroom at school	1=Full participation<br>2=Somewhat limited<br>3=Very limited<br>4=Unable<br>5=Not applicable
612	Social, play and recreational activities with other pupils at school (e.g., “hanging out,” sports, creative arts, lunchtime or recess)	1=Full participation<br>2=Somewhat limited<br>3=Very limited<br>4=Unable<br>5=Not applicable
613	Moving around at school (e.g., to get to and use bathroom, playground, lunch area, library or other rooms and things that are available to other pupils your child’s age)	1=Full participation<br>2=Somewhat limited<br>3=Very limited

		4=Unable<br>5=Not applicable
614	Using educational materials and equipment that are available to other pupils in your child's classroom/s or that have been modified for your child (e.g., books, chairs and desks)	1=Full participation<br>2=Somewhat limited<br>3=Very limited<br>4=Unable<br>5=Not applicable
615	Communicating with other pupils and adults at school	1=Full participation<br>2=Somewhat limited<br>3=Very limited<br>4=Unable<br>5=Not applicable
	DAILY LIVING ACTIVITIES	
616	Household activities (e.g., preparing meals, doing laundry, washing dishes)	1=Full participation<br>2=Somewhat limited<br>3=Very limited<br>4=Unable<br>5=Not applicable
617	Shopping and managing money (e.g., shopping at market, figuring out correct change)	1=Full participation<br>2=Somewhat limited<br>3=Very limited<br>4=Unable<br>5=Not applicable
618	Managing daily schedule (e.g. doing and completing daily activities on time; getting ready for school and doing homework)	1=Full participation<br>2=Somewhat limited<br>3=Very limited<br>4=Unable<br>5=Not applicable
619	Using transportation to get around in the community (e.g. to and from school, work, social or leisure activities) [*using taxi and body*]	1=Full participation<br>2=Somewhat limited<br>3=Very limited<br>4=Unable<br>5=Not applicable
620	School activities and responsibilities (e.g., completion of tasks, punctuality, attendance and getting along with teachers)	1=Full participation<br>2=Somewhat limited<br>3=Very limited<br>4=Unable<br>5=Not applicable

SECTION 700: PLA-D

BASELINE only

Now I would like to ask you some questions about your needs and expectations of the programme

Nr.		CODE
701	What are the three biggest issues in [NAME]'s day-to-day life that you/your child find difficult or need support with?	
702	What are the three biggest issues related to [NAME]'s health you/your child find difficult or need support with?	
703	What are your main goals for attending this group?	

END-LINE only
Now I would like to ask you some questions about your feelings about the programme?

Remind the participant of their goals from the baseline

Nr.		CODE
804	Do you feel that you have achieved or met your goals?	1=Not at all<br>2= Partially<br>3= Fully
805	Please explain	
806	How satisfied were you with the content of the group programme?	1= Very dissatisfied<br>2= Disatisfied<br>3= Neither satisfied nor dissatisfied<br>4= Satisfied<br>5= Very satisfied
807	How satisfied were you with the organization of the programme (e.g. transport, location, refreshments)?	1= Very dissatisfied<br>2= Disatisfied<br>3= Neither satisfied nor dissatisfied<br>4= Satisfied<br>5= Very satisfied
808	How satisfied were you with the facilitators of the programme?	1= Very dissatisfied<br>2= Disatisfied<br>3= Neither satisfied nor dissatisfied<br>4= Satisfied<br>5= Very satisfied

809	Can you suggest ways in which the programme could be improved?	

810	**RECORD THE TIME**	___ Hrs __Min	TIMEPART2END

Closing statement and thank the respondent
INTERVIEWER: This is the end of the interview
This completes the interview. We thank you for your time and answers. I have your contact details and may be in touch again.
