# Supplementary material for: Is it feasible to implement a community-based participatory group programme to address issues of access to healthcare for people with disabilities in Luuka district Uganda? A study protocol for a mixed-methods pilot study
Source: BMJ Open. 2023 Sep 28;13(9):e074217. doi: 10.1136/bmjopen-2023-074217 (PMC10546107; doi:10.1136/bmjopen-2023-074217)
Supplement: Supplementary data [file bmjopen-2023-074217supp003.pdf]

Focus Group Discussion for Participants of PLA-D

Pilot-testing a participatory approach to improve access to healthcare for people with disabilities in Uganda

Objective: To gather information to help further design a participatory approach for people with disabilities to improve health

These questions should be used to guide discussion but do not have to be used in the sequence listed below. The interviewer should follow up on any additional issues that may arise and seem important in relation to the issues above.

Introduction
- Greet them and thank them for their time
- Identify yourself by name and organisation.
- Read out the information sheet. Remind them of confidentiality and anonymity. Check if they have any questions. Remind them that they are free to decline to answer any of the questions or stop the interview at any time.
- Record their consent/assent in the relevant form OR record verbal consent.
- Start recording

Notes: the following details must be recorded in field notes

Participant Code	
Interview date and time	
Interview location or mode (phone, video)	
Interviewer	
Interviewee number	
Gender (number men/women)	
Age (number in broad groups)	
General observations (anything which might impact how the interview is conducted)	

As we have discussed, we are gathering information to help improve the design of the groups about disability that you recently participated in.

1. Share with me your experience participating in the PLA-D groups?

2. What do you think was the purpose of the groups?

3. What did you think about the way the groups were organised?

   Prompt – facilitation, regularity of meetings, location, group facilitator, frequency

4. Are there any sessions that were particularly interesting and important for you, and if so why?

5. What, if any, do you think were the main benefits of the groups?

6. What, if any, were the main problems with the groups?

Prompt – gender, age, different forms of disability, date, location and timing of meetings

7. If we were to plan the groups again, what could we do differently?

8. Is there anything else that you would like to say about the groups that we have not covered?
