# Supplementary material for: Is it feasible to implement a community-based participatory group programme to address issues of access to healthcare for people with disabilities in Luuka district Uganda? A study protocol for a mixed-methods pilot study
Source: BMJ Open. 2023 Sep 28;13(9):e074217. doi: 10.1136/bmjopen-2023-074217 (PMC10546107; doi:10.1136/bmjopen-2023-074217)
Supplement: Supplementary data [file bmjopen-2023-074217supp004.pdf]

Qualitative Interview guide for facilitators of PLA-D

Pilot-testing a participatory approach to improve access to healthcare for people with disabilities in Uganda

Objective: To gather information to help further design a participatory approach for people with disabilities to improve health

These questions should be used to guide discussion but do not have to be used in the sequence listed below. The interviewer should follow up on any additional issues that may arise and seem important in relation to the issues above.

Introduction

- Greet them and thank them for their time
- Identify yourself by name and organisation.
- Read out the information sheet. Remind them of confidentiality and anonymity. Check if they have any questions. Remind them that they are free to decline to answer any of the questions or stop the interview at any time.
- Record their consent/assent in the relevant form OR record verbal consent.
- Start recording

Notes: the following details must be recorded in field notes

Participant Code	
Interview date and time	
Interview location or mode (phone, video, in person)	
Interviewer	
Gender	
Age	
Profession	
General observations (anything which might impact how the interview is conducted)	

As we have discussed, we are gathering information to help improve the design of the groups about disability that you recently facilitated.

1. Share with me your experience being a group facilitator.
2. Can you tell me why you decided to become a group facilitator?

3. What were your initial thoughts of the group and being a group facilitator?

4. What do you think was the purpose of the groups?

5. How was the group attendance at the group? Why do you think people did not come?

6. Can you tell me more about the people in the group? Were there men and women? What about different ages and types of impairment? Were there people with disabilities and caregivers? Who else came?

7. What did you think about the way the groups were organised?

   Prompt – facilitation, regularity of meetings, location,

8. What do you think about the type and level of support that you received as a group facilitator?

9. Are there any sessions that were particularly important or interesting for you, and if so why?

10. What, if any, were the main benefits you perceived of the groups?

11. What actions were taken through or by the groups? What do you think were their effects?

12. Can you describe any changes that you have perceived in your life because of the groups? And in the lives of the group members?

13. What, if any, were the main problems with the groups?

14. Can you think of what any suggestions or feedback would be to someone who as thinking of establishing PLA-D groups?

15. If we were to plan the groups again, what could we do differently?

16. Is there anything else that you would like to say about the groups that we have not covered?
