# Supplementary material for: Is it feasible to implement a community-based participatory group programme to address issues of access to healthcare for people with disabilities in Luuka district Uganda? A study protocol for a mixed-methods pilot study
Source: BMJ Open. 2023 Sep 28;13(9):e074217. doi: 10.1136/bmjopen-2023-074217 (PMC10546107; doi:10.1136/bmjopen-2023-074217)
Supplement: Supplementary data [file bmjopen-2023-074217supp005.pdf]

MRC/UVRI and LSHTM Uganda Research Unit

Qualitative Interview guide for people who did not participate in PLA-D

Pilot-testing a participatory approach to improve access to healthcare for people with disabilities in Uganda

Objective: To gather information to help further design a participatory approach for people with disabilities to improve health

These questions should be used to guide discussion but do not have to be used in the sequence listed below. The interviewer should follow up on any additional issues that may arise and seem important in relation to the issues above.

Introduction

- Greet them and thank them for their time
- Identify yourself by name and organisation.
- Read out the information sheet. Remind them of confidentiality and anonymity. Check if they have any questions. Remind them that they are free to decline to answer any of the questions or stop the interview at any time.
- Record their consent/assent in the relevant form OR record verbal consent.
- Start recording

Notes: the following details must be recorded in field notes

Participant Code	
Interview date and time	
Interview location or mode (phone, video, in person)	
Interviewer	
Gender	
Age	
Profession	
General observations (anything which might impact how the interview is conducted)	

As we have discussed, we are gathering information to help improve the design of the groups about disability.

1. Can you tell me how you first heard about the group?
   - Can you tell me how many times you were invited? How did you receive this invitation?
   - Did you understand that you could join the group at any point? What could have helped to have made this more clear?

2. What do you think was the purpose of the group?

3. Some people in the groups started coming and then stopped, and others decided not to come at all. Can you tell me why you think some people might have decided to stop, or not to join at all? Can you tell me why you decided not to join the group?

Prompt: information, location, timing, frequency, eligibility

4. How much is your health, or difficulties accessing healthcare, an important issue in your life? Please explain your answer.
    - If yes – did you understand that this topic was the focus of the groups?

5. Can you tell me about other groups in the community that you have joined, whether of people with disabilities or not? What was the reason why you joined that group?

6. If we were to plan the groups again, what could we do differently to encourage you to join?

7. Can you tell me what might help others, or yourself, to attend these groups?
    - What kind of information might be useful?
    - Where could they be located?
    - What advice could you give the organisers of these groups on when the groups were held and how often?
    - How could others, or yourself, be made to feel more welcome and that they could be part of the groups?

8. Is there anything else that you would like to say about the group that we have not covered?
