# Supplementary material for: Is it feasible to implement a community-based participatory group programme to address issues of access to healthcare for people with disabilities in Luuka district Uganda? A study protocol for a mixed-methods pilot study
Source: BMJ Open. 2023 Sep 28;13(9):e074217. doi: 10.1136/bmjopen-2023-074217 (PMC10546107; doi:10.1136/bmjopen-2023-074217)
Supplement: Supplementary data [file bmjopen-2023-074217supp006.pdf]

MRC/UVRI and LSHTM Uganda Research Unit

Qualitative Interview guide for people who have participated in PLA-D

Pilot-testing a participatory approach to improve access to healthcare for people with disabilities in Uganda

Objective: To gather information to help further design a participatory approach for people with disabilities to improve health

These questions should be used to guide discussion but do not have to be used in the sequence listed below. The interviewer should follow up on any additional issues that may arise and seem important in relation to the issues above.

Introduction

- Greet them and thank them for their time
- Identify yourself by name and organisation.
- Read out the information sheet. Remind them of confidentiality and anonymity. Check if they have any questions. Remind them that they are free to decline to answer any of the questions or stop the interview at any time.
- Record their consent/assent in the relevant form OR record verbal consent.
- Start recording

Notes: the following details must be recorded in field notes

Participant Code	
Interview date and time	
Interview location or mode (phone, video, in person)	
Interviewer	
Gender	
Age	
Profession	
General observations (anything which might impact how the interview is conducted)	

As we have discussed, we are gathering information to help improve the design of the groups about disability that you recently participated in.

1. Share with me your experience being a member of the group.
2. Can you tell me why you decided to join the group?

MRC/UVRI and LSHTM Uganda Research Unit

3. How many of the group sessions did you attend?
   Probe – if rarely – why?
   Probe – if frequently – why?

4. What do you think was the purpose of the group?

5. What did you think about the way your group was organised?

   Prompt – facilitation, regularity of meetings, location,

6. Are there any sessions that were particularly interesting or useful for you, and if so why?

7. What, if any, were the main benefits you perceived of the groups?

8. What actions were taken through or by your group? What do you think were the effect of your group was?

9. How/ in what ways were you supported by your group for example to attend, to participate?

10. Can you describe any changes that you have perceived in your life because of your group?

11. What interesting or important things have you learnt through participation in your group?

12. What, if any, were the main problems with your group?

13. Can you think of what any suggestions or feedback would be to someone who as thinking of establishing PLA-D groups?

14. If we were to plan the groups again, what could we do differently?

15. Is there anything else that you would like to say about your group that we have not covered?
