# Supplementary material for: Is it feasible to implement a community-based participatory group programme to address issues of access to healthcare for people with disabilities in Luuka district Uganda? A study protocol for a mixed-methods pilot study
Source: BMJ Open. 2023 Sep 28;13(9):e074217. doi: 10.1136/bmjopen-2023-074217 (PMC10546107; doi:10.1136/bmjopen-2023-074217)
Supplement: Supplementary data [file bmjopen-2023-074217supp007.pdf]

MRC/UVRI and LSHTM Uganda Research Unit

Observation of PLA-D group

The Missing Billion: Pilot-testing PLA-D groups to improve access to healthcare for people with disabilities in Uganda

Objective: To gather information to help further design a PLA-D group

Introduction

- Greet them and thank them for their time
- Identify yourself by name and organisation.
- Read out the information sheet. Remind them of confidentiality and anonymity. Check if they have any questions.
- Record their consent/assent in the relevant form OR record verbal consent.

Notes: the following details must be recorded in field notes

Group Code	
Observation date and time	
Observation location	
Observer name	
Participant gender (number men/women)	
General observations (anything which might impact how the interview is conducted)	

Observe the session and record your observations on the following page:

MRC/UVRI and LSHTM Uganda Research Unit

Facilitation group checklist	Activity demonstrated	Observed		Comment
		Yes	No	
Logistics	The group was held at the date, time and location that was expected			
	Number of facilitators who attended was the same as expected			
	Number of participants who attended were the same as expected			
	Group was conducted and ran according to schedule			
Environment created by facilitator	Room/location set up was in a circular pattern with all participants included			
	Room/location was accessible			
	The plan for the group session was discussed			
	Facilitators endeavoured to engage all the participants.			
	Facilitators used inclusive/accessible communication styles			
Response of participants	All participants contributed during the group, e.g. speaking to each other, speaking aloud in the group			
	Participants were distracted by competing interests, phone calls			
	There was a clear outcome to the session (e.g. barrier identified, plan agreed)			
	Participants appeared positive and engaged about the group			
