# Supplementary material for: Is it feasible to implement a community-based participatory group programme to address issues of access to healthcare for people with disabilities in Luuka district Uganda? A study protocol for a mixed-methods pilot study
Source: BMJ Open. 2023 Sep 28;13(9):e074217. doi: 10.1136/bmjopen-2023-074217 (PMC10546107; doi:10.1136/bmjopen-2023-074217)
Supplement: Author's manuscript [file bmjopen-2023-074217.draft_revisions.pdf]

BMJ Open is committed to open peer review. As part of this commitment we make the peer review history of every article we publish publicly available.

When an article is published we post the peer reviewers' comments and the authors' responses online. We also post the versions of the paper that were used during peer review. These are the versions that the peer review comments apply to.

The versions of the paper that follow are the versions that were submitted during the peer review process. They are not the versions of record or the final published versions. They should not be cited or distributed as the published version of this manuscript.

BMJ Open is an open access journal and the full, final, typeset and author-corrected version of record of the manuscript is available on our site with no access controls, subscription charges or pay-per-view fees (http://bmjopen.bmj.com).

If you have any questions on BMJ Open's open peer review process please email info.bmjopen@bmj.com

BMJ Open

A community-based participatory group programme to improve access to healthcare for people with disabilities in Uganda: a pilot study protocol

Journal:	*BMJ Open*
Manuscript ID	bmjopen-2023-074217
Article Type:	Protocol
Date Submitted by the Author:	30-Mar-2023
Complete List of Authors:	Kuper, Hannah; London School of Hygiene & Tropical Medicine, Department of Population Health<br>Ssemata, Andrew Sentoogo; MRC/UVRI and LSHTM Uganda Research Unit<br>Smythe, Tracey; London School of Hygiene and Tropical Medicine, Clinical Research<br>Drazdzewska, Joanna; Women and Children First<br>Waiswa, Peter; Makerere University<br>Kagurusi, Patrick; Amref Health Africa in Uganda<br>Rosato, Mikey; Women and Children First (UK)<br>Mbazzi, Femke Bannink; London School of Hygiene & Tropical Medicine, Department of Population Health; UVRI and LSHTM Uganda Research Unit
Keywords:	MENTAL HEALTH, Health Services Accessibility, Developmental neurology & neurodisability < PAEDIATRICS

SCHOLARONE™
Manuscripts

A community-based participatory group programme to improve access to healthcare for people with disabilities in Uganda: a pilot study protocol

Hannah Kuper[1], Andrew Sentoogo Ssemata[2,3], Tracey Smythe[1,4], Joanna Drazdzewska[5], Peter Waiswa[6], Patrick Kagurusi[7], Mikey Rosato[5], Femke Bannink Mbazzi[1,2]

[1]International Centre for Evidence in Disability, Department of Population Health, London School of Hygiene & Tropical Medicine, London, UK

[2] MRC/UVRI and LSHTM Uganda Research Unit, Entebbe, Uganda.

[3] Department of Global Health and Development, London School of Hygiene & Tropical Medicine, London, UK

[4] Division of Physiotherapy, Department of Health and Rehabilitation Sciences, Stellenbosch University, Cape Town, South Africa

[5] Women and Children First (UK), London, UK

[6] School of Public Health, Makerere University, Kampala, Uganda

[7] Amref Health Africa in Uganda, Kampala, Uganda

Author for correspondence: Hannah Kuper, International Centre for Evidence in Disability, Department of Population Health, London School of Hygiene & Tropical Medicine, Keppel Street, London WC1E 7HT, UK. Email: Hannah.kuper@lshtm.ac.uk

KEYWORDS
Disability; community-based participatory learning and action; pilot; evaluation; Uganda; Healthcare access

For peer review only - http://bmjopen.bmj.com/site/about/guidelines.xhtml

ABSTRACT

Introduction

On average, people with disabilities experience worse health and face many difficulties in accessing healthcare. Yet, evidence on how to overcome these barriers is lacking. Participatory approaches are gaining prominence as they can generate low-cost, appropriate and scalable solutions. This study protocol is for the pilot-testing of the co-created Participatory Learning and Action for Disability (PLA-D) groups to assess feasibility.

Methods and analysis

We will pilot-test PLA-D in five groups in Luuka district, Uganda during 2023. Each group will include approximately 20 members (people with disabilities, family members, carers) who will meet every 2-3 weeks over a 9-11 month period. The groups, guided by a trained facilitator, will identify issues about health and healthcare access and plan and implement locally-generated solutions (e.g. raising awareness of rights, advocacy and lobbying, establishing health savings and financing schemes). We will collect diverse sources of data to assess feasibility: 1) in-depth interviews and focus group discussion with group participants, non-participants, and group facilitators, 2) monitoring of group activities; 3) direct observation of groups, 4) quantitative survey of group participants at baseline and endline. Data analyses will be undertaken to assess feasibility in terms of: acceptability, demand, implementation and practicality. Limited efficacy will be assessed to develop and refine evaluation tools in preparation for a future trial.

Ethics and dissemination

Ethical approval for the study has been received by the London School of Hygiene & Tropical Medicine, and the Uganda Virus Research Institute ethics committees. Informed consent will be obtained from all study participants, making adaptations for people with disabilities as necessary. We will reach different groups for our dissemination activities, including 1) People with disabilities (e.g. community meetings); 2) Policy and programme stakeholders in Uganda and international (e.g. individual meetings, evidence briefs); 3) Academics (journal articles, conference/seminar presentations).

Strengths and limitations of this study

- PLA-D will be the first community-based facilitator-led participatory learning and action group programme designed to improve overall access to healthcare for people with disabilities. If feasible, it could be scaled to other settings.
- Feasibility of the programme will be assessed using mixed-method approaches, with participation from people with disabilities.
- The feasibility assessment will inform the preparation for a future trial of the programme.
- A limitation is that the pilot-study is only in one setting and will allow assessment of some domains of the Bowen feasibility framework (acceptability, demand, implementation, practicality, limited efficacy) but not others (adaptation, integration, expansion).
- The limited number of participants may make it difficult to draw inferences for feasibility for different sub-groups, such as people with different impairment types.

For peer review only

INTRODUCTION

There are at least 1.3 billion people with disabilities globally, more than 80% of whom live in Low and Middle-Income Countries (LMICs). [1 2] People with disabilities include those who have long-term physical, mental, intellectual or sensory impairments which, in interaction with various barriers, may hinder their full and effective participation in society on an equal basis with others.[2 3] People with disabilities face a wide range of discrimination and negative attitudes. [2] They are consequently are more likely to be poor, and excluded from education, employment, and societal participation.[2] Another key challenge facing many people with disabilities is exclusion from healthcare and difficulties achieving good health, [1 2 4-7 5] which is the focus of the current study.

On average, people with disabilities have worse health than others in the population.[1 2 4-6] By definition, people with disabilities have an underlying health condition and impairment (e.g. diabetes, physical impairment), which are linked to other health risks (e.g. stroke, pressure sores). They are also on average older, poorer and more marginalised, and have a higher prevalence of a range of risk factors (e.g. violence, physical inactivity, diabetes, hypertension). [1 2 6] People with disabilities therefore, on average, have greater general healthcare needs than others in the population. [1 4] They will also require regular healthcare services like anyone else in the population (e.g. vaccinations) and potentially also specialised healthcare services (e.g. physiotherapy). However, they frequently experience widespread barriers to accessing healthcare, including lack of accessible transport and facilities, poor skills of healthcare providers around disability, and high costs.[1 7] Consequently, people with disabilities are 41-57% more likely to have unmet healthcare needs, according to the WHO's World Report on Disability. [2] Quality and affordability of healthcare services is also often worse for people with disabilities; They are twice as likely to find healthcare providers skills and facilities inadequate, three times more likely to be denied healthcare, and four times more likely to be treated badly in the healthcare system. [2] The WHO report also found that half of people with disabilities cannot afford healthcare, and they are 50% more likely to suffer catastrophic health expenditure. [2] As a result of all these factors, people with disabilities on average have worse health and higher mortality rates than others in the population, including in the African context. [2 8-11] These patterns can also be observed in Uganda, the context for the current study, where the 2014 census estimated that 12.5% of the population have disabilities.[12] Studies have shown that people with disabilities in Uganda are more likely to experience poverty and exclusion, poor health, and difficulties accessing services and information.[12-17]

Improving access to healthcare for people with disabilities is therefore an important priority for a number of reasons. We will fail to achieve health targets and development goals without an explicit focus on disability, including Sustainable Development Goal (SDG) 3 on "Good health for all" and Universal Health Coverage (UHC).[18] Other SDGs, including those related to education and employment, will also not be met if people with disabilities continue to have poor health. Exclusion from healthcare is a violation of rights of people with disabilities, as set out in the United Nations Convention on the Rights of Persons with Disabilities (UNCRPD) and in the laws of most countries, including Uganda.[18 19] Good healthcare also matters to people with disabilities and their families as it affects their ability to survive and enjoy good health. Improving healthcare access for people with disabilities also has the potential to be cost-saving, [1] as it may prevent unnecessary cost for the health system and improve health services for all through universal design.

For peer review only - http://bmjopen.bmj.com/site/about/guidelines.xhtml

Rationale for development of a participatory intervention to improve access to healthcare for people with disabilities

Two systematic reviews on access to general and specialist healthcare for people with disabilities in LMICs found few examples of interventions to address the widespread gaps observed. [20 21] An Evidence and Gap Map on disability-inclusive development also failed to identify good examples of disability-inclusive health interventions. [22] The WHO has made specific recommendations for the promotion of disability-inclusive health,[1 2] including 1) removing physical barriers to health facilities, information and equipment, 2) making healthcare affordable, 3) training all healthcare workers in disability issues including rights, 4) investing in specific services such as rehabilitation. These "top-down" interventions are important, but unlikely to be sufficient since barriers will vary locally.[7] Participation of people with disabilities is therefore critically important for developing new locally- appropriate solutions – particularly in light of the "Nothing about us, without us" ethos of the disability movement. Participatory approaches are also recommended by the new WHO report on health equity for persons with disabilities.[1]

Participatory approaches are gaining attention from the global health community as they can empower communities to work together to identify local issues and together generate low-cost, appropriate and scalable solutions. A leading example is the Participatory Learning and Action (PLA) approach, which was designed to reduce maternal and neonatal mortality. [23] Through PLA, women's groups are convened and are facilitated to identify underlying barriers to care and care-seeking and then develop, implement and evaluate local solutions to address these issues (Figure 1). Examples of solutions implemented include the creation of emergency crisis funds to pay for medical care for those in poverty, arranging a bicycle ambulance to bring people to hospital, and lobbying local health authorities for more staff. PLA supports care-seeking and home-based care practices through these pathways, and thereby improves health and wellbeing and reduces mortality in women and new-borns. A meta-analysis of 7 RCTs from 4 countries found that PLA was associated with a 22% reduction in maternal mortality and 20% reduction in neonatal mortality. [23] Sub-group analysis of 4 RCTs where at least 30% of pregnant women participated in groups showed even greater reductions in both maternal mortality (49%) and neonatal mortality (33%).[23] Evidence also shows that the PLA methodology is sustainable,[24] scalable,[25] and equitable.[26] It is recommended by WHO as a strategy for improving maternal and new-born health and reducing mortality, particularly in rural settings where mortality rates are high and access to services is low. [27] PLA has been successfully implemented in 15 countries, including in several settings Uganda.

To date, PLA has not been used explicitly for people with disabilities. However, evidence from Nepal showed that there was no difference in group attendance between women with and without disabilities. [28] A non-PLA participatory approach has been used in the Philippines to try to improve access to sexual and reproductive healthcare for people with disabilities through establishment of community groups - W-DARE project (Women with Disability taking Action on REproductive and sexual health. [29] Five groups of women with disabilities (established by impairment type) and one group of parents of children with disabilities met fortnightly for ten meetings to identify and discuss key sexual and reproductive healthcare access issues. A qualitative evaluation highlighted positive benefits of the programme, including increased knowledge and confidence of the women to seek services. [30] Other participatory approaches to promote disability inclusive-health are lacking.

We hypothesise that PLA for people with disabilities (PLA-D) would improve healthcare access, health and wellbeing and reduce mortality of people with disabilities in Uganda. The PLA-D approach is expected to work from the bottom-up as people with disabilities identify and tackle locally-relevant barriers. The potential drivers of action are that group meetings

For peer review only - http://bmjopen.bmj.com/site/about/guidelines.xhtml

allow people with disabilities to meet and identify local issues, and develop and implement solutions that are self-resourced. Existing programmes show that solutions implement address a range of issues, such as lack of awareness (e.g. education on health issues and rights), lack of decision-making authority on health (e.g. lobby families to fund healthcare of person with disabilities) or poverty (e.g. livelihood programmes). The intervention will be supported by health system strengthening activities to improve healthcare access and quality by improving the knowledge, attitudes and skills of healthcare workers and helping identify issues around accessibility of facilities. Furthermore, groups also frequently undertake advocacy activities, for instance, to encourage other members in the community to become involved and take action (e.g. support transport to healthcare facilities). Groups can also lobby decision-makers to encourage policy and programme change and raise awareness on disability. Through these actions, people with disabilities are anticipated to experience better access to healthcare, improved health and wellbeing, and ultimately reduced mortality. The current protocol focusses on the group meetings (PLA-D). We have been guided by the MRC framework in the development of the PLA-D approach, and proposed feasibility assessment, and in the future plan to undertake an evaluation of the intervention.[31]

Adaptation of PLA for people with disabilities
In 2022, we undertook qualitative research with people with disabilities with a range of impairment types (n=27), healthcare workers (n=15) and other stakeholders, including carers (n=11), to describe barriers and facilitators to accessing healthcare by people with disabilities in Uganda and assess the demand for PLA-D. We also reviewed the literature to identify good practice for improving access to healthcare for people with disabilities, including existing systematic reviews,[20-22] key source documents,[2 6 32 33] and a good practice compendium.[34] This activity was supplemented through interviews with key stakeholders, including PLA experts, Organizations of Persons with Disabilities (OPDs), inclusive health experts.

We held a 4-day design workshop in October 2022 to co-create the adapted PLA approach to be accessible, relevant and appropriate for people with disabilities in Uganda (PLA-D), facilitated by Women and Children First. Participants included the research team (n=7), PLA experts (including Amref and Makerere University n=5), health system stakeholders (e.g. District Health Officers n=2), district disability focal person from the community development office (n=1), people with disabilities and representatives from National OPDs (n=8). The group worked together to agree how to adapt the logistics of the intervention delivery (e.g. identifying who the facilitators should be, where groups should be convened, addressing accessibility) and identified appropriate local implementation and OPD partners (Figure 2). The PLA content was then adapted with reference to recommendations from the PLA evidence base and literature, including the facilitator manual, materials (e.g. pictures, cards) and session plans. We updated the Theory of Change for PLA to reflect how the intervention is anticipated to have the expected impact (Figure 3).

This protocol is for a study to pilot-test the co-created Participatory Learning and Action for Disability (PLA-D) groups in order to assess their feasibility, and to inform the design of a future cluster randomised controlled trial.

For peer review only - http://bmjopen.bmj.com/site/about/guidelines.xhtml

METHODS AND ANALYSIS

Overview

A mixed-methods pilot-study will be undertaken in 2023 to assess the feasibility of the PLA-D groups and inform the future development of a cluster randomised controlled trial. The research will be undertaken in Luuka district, Eastern Uganda, as a partnership between researchers from the London School of Hygiene & Tropical Medicine, MRC/ UVRI & LSHTM Uganda Research Unit ("Uganda Research Unit"), Makerere University, and the NGOs Women and Children First, UK and Amref Health Africa in Uganda.

Pilot of the content and implementation of PLA-D groups

We will establish five pilot PLA-D groups in one sub-country in Luuka district. In collaboration with the District Health Officers, we will select the communities to represent a variety of contexts (e.g. very rural or semi-urban, remote or less remote areas). We will work with local community members (e.g. Community health worker [CHWs] or Community Health Extension Workers [CHEWs]) to identify people with disabilities in the community. People with disabilities will then be given information about the purpose and structure of the PLA-D groups and invited to attend. Membership will also be open to family members/ caregivers of people with disabilities, although emphasis will be given on the participation of people with disabilities. We anticipate that each group will include approximately 20 people, potentially at least half of whom will be people with disabilities.

Groups will meet every 2-3 weeks over a 9 month period, and will go through the PLA cycle (Figure 1). The groups will together choose the location of the meetings, ideally selected to promote accessibility, as well as the timing. The PLA cycle includes sessions on identifying issues in accessing healthcare, developing and implementing solutions (e.g. savings schemes, growing nutritious food), and self-evaluating their impact. Other community members will be reached through regular community members. The groups will be facilitated by a trained facilitator (person with disabilities from the local community and/or a CHW). They will be trained by the NGO Women and Children First, with support from the NGO Amref and the researchers at the Uganda Research Unit. The facilitators will be supported by trained supervisors and managers, which will be coordinated by the NGO Amref.

Feasibility assessment

We will use the Bowen framework to consider the feasibility of the PLA-D groups.[35] Our main focus will be on the first four components of the framework: acceptability, demand, implementation, and practicality. We will not consider the components of adaptation, integration and expansion for this pilot study, as they relate to issues of scaling existing programmes. Limited-efficacy testing will be undertaken to develop and refine evaluation tools of the groups to inform a future cluster randomised controlled trial.

There are four main sources of data collection (supplement file).

1) Programme monitoring

Attendance of sessions and frequency of sessions will be monitored through a simple register which will be collected by the facilitators.

2) Pre-post quantitative survey

A survey will be completed by adult PLA-D group participants with disabilities at the programme first and last session (either directly, or via a proxy). These will be developed in

For peer review only - http://bmjopen.bmj.com/site/about/guidelines.xhtml

English and translated into Luganda and Lusoga (administered in the language of choice of the respondent) and will include:

- Socio-demographic characteristics of the participant
- Disability assessment (Washington Group Enhanced Short Set)
- Quality of Life (WHO-BREF)
- Health condition and nutrition
- Health care utilization
- Participation
- Attitudes
- Main goals for the intervention*
- Review of goals achieved**
- Satisfaction with group programme**

*Only asked at baseline; **only asked at end-line

Questionnaires are based upon existing tools (WHOQOL BREF, WOPS questionnaire from cohort in Uganda, WHO Model Disability Survey, and SINTEF survey), which have all been widely used. Any potentially controversial questions have been removed (i.e. SINTEF questions on violence). A separate questionnaire has been developed for children aged 5-17 years, to be completed by the parent/carer as a proxy. Questionnaire data will not be collected for children <5 years.

All questionnaires will be interviewer- administered in order to maintain consistency and uniformity in interpretation of the questions. Interviews will be undertaken by researchers from the Uganda Research Unit. All questionnaires will be translated in Lusoga and Luganda, the most common local languages in Luuka district. Proxy respondents will be used for adults with difficulties understanding or communicating, and with children aged <18 years. Children aged 8-17 will be asked to give assent to the questionnaire being completed on their behalf.

3) Semi-structured interviews and Focus Group Discussions

Qualitative data will be collected from a range of groups, led by researchers from Uganda Research Unit.

Semi-structured in-depth interviews (IDIs) will be conducted with the group facilitators after each session. The researchers will conduct phone interviews with the group facilitator after each session to review the progress of the session, identify areas that went well, challenges that arose and areas for improvement. This information will be used to allow fast-track learning so that future sessions by that or other groups can be adapted in line with recommendations (e.g. tips for promoting inclusion, location of meetings, etc).

Additionally, semi-structured IDIs and focus group discussions (FGDs) will be undertaken with group participants and facilitators after the final session. The IDI will be undertaken in the language of choice of the respondent (English or Luganda or Lusoga) with a focus on: 1) satisfaction with the groups, 2) perceived challenges, facilitators and barriers to successful group implementation, 3) group capacity, 4) areas for improvement, and 5) perceived positive and negative impacts. Interviews will conducted face-to-face (wherever possible) or remotely. Proxy respondents will be used for adults with difficulties understanding or communicating, and with children aged <18 years.

The IDIs and FGDs will include:

- FGDs with PLA-D participants of the five groups excluding those who participated in the IDIs (6-8 participants per group, 30-40 in total).

For peer review only - http://bmjopen.bmj.com/site/about/guidelines.xhtml

- IDIs with 10-15 PLA-D group participants (2-3 participants per group). Participants will be selected to reflect a range of type and severity of disability, gender and socio-economic profile. They will include people with disabilities and caregivers of persons with disabilities and PLA-D members without disabilities. These interviews will support triangulation of data from the FGDs and further inquiry of areas for further exploration raised during the FDGs.

- IDI with 10-15 people eligible to be PLA-D group participants, but who attended 3 or fewer sessions (including no sessions). Participants will be selected to reflect a range of type and severity of disability, gender and socio-economic profile. They will include people with disabilities and caregivers of persons with disabilities.

- IDI with each of the group facilitators (total = 5).

Topic guides are available in the supplement.

We anticipate that each interview will take approximately 45-60 minutes, and the Focus Group Discussion 60-90 minutes. Carer/proxy interviews will be conducted for people with severe difficulties understanding or communicating even with available adaptations (e.g. people with hearing loss, illiterate and with no knowledge of sign language; people with severe intellectual/cognitive impairments). Inclusion of people with disabilities will be supported through sign language interpretation as needed, accessible interview sites and transport, and researchers skilled at communicating with people with cognitive impairments. Carer/proxy interviews will also be used for children with disabilities below the age of 18 years.

4) <u>Direct observation of groups</u>

Direct observation of sessions will be undertaken by the local researchers using a checklist guide (Supplement - checklist). The checklist will contain information about the participatory approaches used by facilitators, the level of interest and engagement of participants and notes of any aspects of the group meeting which went well or did not go well.

Data management and storage

The interviewers will audio record the interviews/focus group discussion. Interviews will be transcribed and translated to English (for those conducted in Luganda or Lusoga). Anonymised transcripts with pseudonyms will be used during the analysis. Quantitative survey data will be anonymised using participant codes and entered into a REDCap database for analysis. All data will be kept as per General Data protection regulation requirements.

Data will be kept strictly confidential (e.g. anonymisation of data to remove personal identifiers, upload/download of data through secure networks and password protecting files). Names of participants will not be given on any documentation and no individuals will be identified in any publication.

Analysis plan

We will use the data to evaluate the feasibility of PLA-D implementation focusing on four of the Bowen components of feasibility (Table 1).[35] A thematic approach will be used to analyse qualitative data. The data will be coded using NVivo 12, specialist software for qualitative data analysis, and the data will be analysed to develop a fuller framework of themes and subthemes. Particular attention will be given to the feasibility of implementation for people across the full range of impairment types, and whether it addresses the needs of both adults and children.

For peer review only

Quantitative data will be analysed using STATA to provide indicative limited efficacy testing, in terms of change in knowledge, attitudes and behaviours, self-rated health, and health-seeking behaviour. Indicators for inclusion in the future randomised controlled trial will be considered. Indication will also be given of potential effect sizes for the planned RCT.

Patient and public involvement

The development and pilot-testing of PLA-D will be in partnership with people with disabilities. During development phase this included: sharing plans with OPDs and people with disabilities; interviewing people with disabilities on their perceived healthcare needs and suggestions for group activities; including people with disabilities in the advisory group and in the co-design workshop. People with disabilities will also have strong involvement in the implementation and pilot-testing of PLA-D, as: group members; group facilitators (wherever possible); research study participants; researchers/data collectors (wherever possible); advisory group members.

ETHICS AND DISSEMINATION

Ethics and consent

Ethical approval for the study has been received from the London School of Hygiene & Tropical Medicine ,the Uganda Virus Research Institute ethics committee, and the Ugandan National Council for Science and Technology.

Consent to participate in the study will be sought from each participant only after a full explanation has been given, an information leaflet offered and time allowed for consideration. Signed participant consent will be obtained (including thumb print if necessary). The right of the participant to refuse to participate without giving reasons will be respected. All participants are free to withdraw at any time from the study without giving reasons and without prejudicing further treatment or PLA-D group participation. For any participant with communication or intellectual impairments, we will seek consent from their caregiver. Carer/parent interviews will also be used for children with disabilities below the age of 18. We will seek verbal assent from children aged 8-17 years for a questionnaire to be completed on their behalf.

There is a chance that participants may become distressed or upset during the interviews when discussing stigma and discrimination. To minimise the risk of distress during interviews/questionnaires/focus group discussions: 1) the right of the participant to refuse to answer questions or withdraw from the study at any point will be emphasised 2) Interviewers will be trained to listen non-judgementally, provide short breaks, be aware of signs of distress, fatigue or anxiety and to address concerns appropriately and signpost to available support mechanisms.

Referrals and reimbursement

Group facilitators will make PLA-D group participants aware of local health services available and will encourage them to attend for services if they have an expressed healthcare need. If disclosures are made in the group of safety concerns (e.g. experience of violence) then the group facilitator will liaise with the local researcher for course of action.

We will provide reimbursement of 25,000 Ugandan Shillings (approximately £5.60) for participants in interviews, questionnaires, or focus group discussions to compensate them for their time, and in compliance with Ugandan guidance. Refreshments will be provided at group meetings.

For peer review only - http://bmjopen.bmj.com/site/about/guidelines.xhtml

Dissemination plan

Research is most worthwhile if it can be used to improve policy and practice. We will develop tailored strategies for engagement and communication with our key audience to facilitate influence of policy and practice, using our strong national and international networks, including:

- Community members and people with disabilities: hosting dissemination meetings in the community and with OPDs.

- Policy and programme stakeholders in Uganda (e.g. health systems actors, OPDs and NGOs): Individual meetings with key organizations, production of policy and evidence briefs, relevant to Uganda.

- International policy and programme stakeholders: Production of policy and evidence briefs, including through the Missing Billion Initiative, meetings with key stakeholders, contribution to WHO World Report on Disability and Health.

- Academics: Peer-reviewed journal articles, presentations at conferences/seminars/webinars, and use of material in teaching, including LSHTM's online teaching and online research seminars (e.g. LSHTM, Uganda Research Unit).

For peer review only

REFERENCES

1. WHO. Global report on health equity for persons with disabilities. Geneva: World Health Organzation, 2022.
2. WHO. World Report on Disability. Geneva: World Health Organisation 2011.
3. UN. Convention on the rights of persons with disabilities. New York: United Nations, 2006.
4. Kuper H, Heydt P. The Missing Billion. London: LSHTM, 2019.
5. Missing Billion Initiative and Clinton Health Access Inititiative. Reimagining health systems that expect, accept and connect 1 billion people with disabilities. Missing Billion Initiative and Clinton Health Access Inititiative, 2022.
6. UNDESA. UN Flagship Report on Disability and Development: UNDESA, 2018.
7. Hashemi G, Wickenden M, Bright T, et al. Barriers to accessing primary healthcare services for people with disabilities in low and middle-income countries, a Meta-synthesis of qualitative studies. *Disabil Rehabil* 2020:1-14. doi: 10.1080/09638288.2020.1817984 [published Online First: 2020/09/22]
8. Prynn JE, Dube A, Mkandawire J, et al. Self-reported disability in relation to mortality in rural Malawi: a longitudinal study of over 16 000 adults. *BMJ Open* 2020;10(8):e034802. doi: 10.1136/bmjopen-2019-034802 [published Online First: 2020/08/30]
9. Kuper H, Mathenge W, Macleod D, et al. Mortality during 6 years of follow-up in relation to visual impairment and eye disease: results from a population-based cohort study of people aged 50 years and above in Nakuru, Kenya. *BMJ Open* 2019;9(6):e029700. doi: 10.1136/bmjopen-2019-029700 [published Online First: 2019/06/12]
10. Gómez-Olivé FX, Thorogood M, Bocquier P, et al. Social conditions and disability related to the mortality of older people in rural South Africa. *Int J Epidemiol* 2014;43(5):1531-41. doi: 10.1093/ije/dyu093 [published Online First: 2014/05/20]
11. Abuga JA, Kariuki SM, Kinyanjui SM, et al. Premature mortality in children aged 6-9 years with neurological impairments in rural Kenya: a cohort study. *Lancet Glob Health* 2019;7(12):e1728-e35. doi: 10.1016/s2214-109x(19)30425-5 [published Online First: 2019/10/28]
12. Uganda Bureau of Statistics. The National Population and Housing Census 2014 – National Analytical Report on persons with disabilities. Kampala, Uganda: Uganda Bureau of Statistics, 2019.
13. Abimanyi-Ochom J, Mannan H, Groce NE, et al. HIV/AIDS knowledge, attitudes and behaviour of persons with and without disabilities from the Uganda Demographic and Health Survey 2011: Differential access to HIV/AIDS information and services. *PLoS One* 2017;12(4):e0174877. doi: 10.1371/journal.pone.0174877 [published Online First: 2017/04/14]
14. Wandera SO, Golaz V, Kwagala B, et al. Factors associated with self-reported ill health among older Ugandans: a cross sectional study. *Arch Gerontol Geriatr* 2015;61(2):231-9. doi: 10.1016/j.archger.2015.05.006 [published Online First: 2015/06/06]
15. Nakamanya S, Siu GE, Lassman R, et al. Maternal experiences of caring for an infant with neurological impairment after neonatal encephalopathy in Uganda: a qualitative study. *Disabil Rehabil* 2015;37(16):1470-6. doi: 10.3109/09638288.2014.972582
16. Scholten F, Mugisha J, Seeley J, et al. Health and functional status among older people with HIV/AIDS in Uganda. *BMC Public Health* 2011;11:886.
17. Nyirenda M, Newell M-L, Mugisha J, et al. Health, wellbeing, and disability among older people infected or affected by HIV in Uganda and South Africa. *Global health action* 2013;6:19201.

For peer review only

18. Kuper H, Hanefeld J. Debate: can we achieve universal health coverage without a focus on disability? *BMC Health Serv Res* 2018;18(1):738. doi: 10.1186/s12913-018-3547-2 [published Online First: 2018/09/28]
19. Government of Uganda. Uganda Persons with Disabilities Act, 2020, 2020.
20. Bright T, Kuper H. A Systematic Review of Access to General Healthcare Services for People with Disabilities in Low and Middle Income Countries. *Int J Environ Res Public Health* 2018;15(9) doi: 10.3390/ijerph15091879 [published Online First: 2018/09/12]
21. Bright T, Wallace S, Kuper H. A Systematic Review of Access to Rehabilitation for People with Disabilities in Low- and Middle-Income Countries. *Int J Environ Res Public Health* 2018;15(10) doi: 10.3390/ijerph15102165 [published Online First: 2018/10/04]
22. Saran A, White H, Kuper H. Evidence and gap map of studies assessing the effectiveness of interventions for people with disabilities in low-and middle-income countries. *Campbell Systematic Reviews* 2020;16(1)
23. Prost A, Colbourn T, Seward N, et al. Women's groups practising participatory learning and action to improve maternal and newborn health in low-resource settings: a systematic review and meta-analysis. *Lancet* 2013;381(9879):1736-46. doi: 10.1016/s0140-6736(13)60685-6 [published Online First: 2013/05/21]
24. Sondaal AEC, Tumbahangphe KM, Neupane R, et al. Sustainability of community-based women's groups: reflections from a participatory intervention for newborn and maternal health in Nepal. *Community Dev J* 2019;54(4):731-49. doi: 10.1093/cdj/bsy017 [published Online First: 2019/12/31]
25. Nair N, Tripathy PK, Gope R, et al. Effectiveness of participatory women's groups scaled up by the public health system to improve birth outcomes in Jharkhand, eastern India: a pragmatic cluster non-randomised controlled trial. *BMJ Glob Health* 2021;6(11) doi: 10.1136/bmjgh-2021-005066
26. Morrison J, Osrin D, Alcock G, et al. Exploring the equity impact of a maternal and newborn health intervention: a qualitative study of participatory women's groups in rural South Asia and Africa. *Int J Equity Health* 2019;18(1):55. doi: 10.1186/s12939-019-0957-7 [published Online First: 2019/04/12]
27. WHO. Every Newborn Action Plan. Geneva: WHO, 2014.
28. Morrison J, Colbourn T, Budhathoki B, et al. Disabled women's attendance at community women's groups in rural Nepal. *Health Promot Int* 2017;32(3):464-74. doi: 10.1093/heapro/dav099 [published Online First: 2015/11/01]
29. Vaughan C, Zayas J, Devine A, et al. W-DARE: a three-year program of participatory action research to improve the sexual and reproductive health of women with disabilities in the Philippines. *BMC Public Health* 2015;15:984. doi: 10.1186/s12889-015-2308-y [published Online First: 20150929]
30. Devine A, Ignacio R, Prenter K, et al. "Freedom to go where I want": improving access to sexual and reproductive health for women with disabilities in the Philippines. *Reprod Health Matters* 2017;25(50):55-65. doi: 10.1080/09688080.2017.1319732 [published Online First: 20170511]
31. MRC. Developing and evaluating complex interventions. London: MRC, 2006.
32. WHO. Policy brief on disability-inclusive health systems. Copenhagan, Denmark: WHO EURO, 2021.
33. UNDSPD U. Toolkit on disability for Africa; inclusive health services for persons with disabilities, 2016.
34. Billion M. Good Practice Compendium 2022 [Available from: https://www.themissingbillion.org/good-practices accessed 08/11 2022.
35. Bowen DJ, Kreuter M, Spring B, et al. How we design feasibility studies. *Am J Prev Med* 2009;36(5):452-7. doi: 10.1016/j.amepre.2009.02.002 [published Online First: 2009/04/14]

For peer review only - http://bmjopen.bmj.com/site/about/guidelines.xhtml

AUTHORS CONTRIBUTIONS

The study concept and design was conceived by HK, TS, FBM and PW. JD, MR, PK and PW informed the development of PLA-D. FBM and ASS will lead on the data collection. Analysis will be performed by HK, FBM and ASS. HK prepared the first draft of the manuscript. All authors provided edits and critiqued the manuscript for intellectual content.

FUNDING STATEMENT

This work is supported by NIHR grant number NIHR301621. Conceptualization of the programme was supported by the PENDA grant from the Foreign, Commonwealth and Development Office.

COMPETING INTERESTS

None declared

For peer review only

Figure 1: PLA cycle

For peer review only

Figure 2: Decisions reached on design of PLA-D

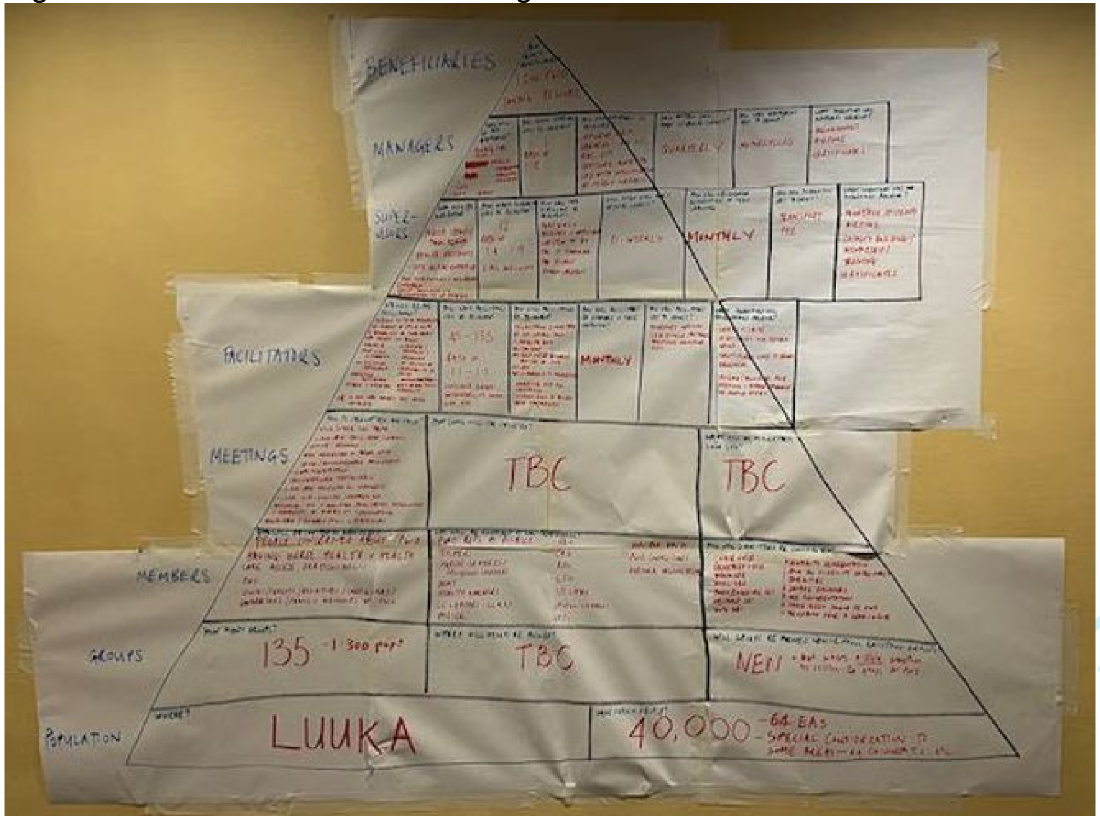

view only

Figure 3: PLA-D Theory of Change

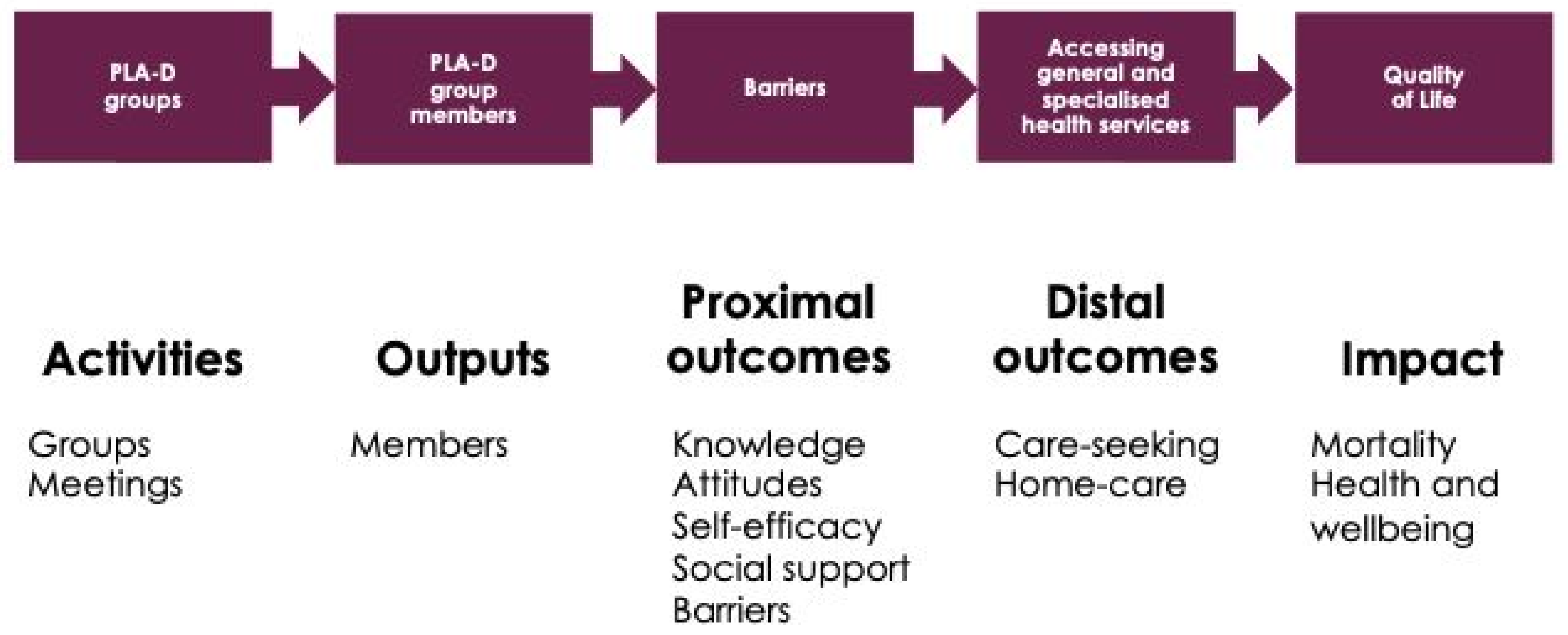

only

Table 1: Feasibility assessment of PLA-D: domains explored and data to be used [35]

Bowen domain	To what extent…..	Example outcomes	Data used
Acceptability	… is PLA-D judged as suitable, satisfying or attractive to program recipients (people with disabilities and other potential PLA-D members)	Reported satisfaction; perceived appropriateness; intent to continue use	Direct observation; Qualitative data; Questionnaire
Demand	… is PLA-D likely to be used?	PLA-D group attendance; Perceived demand; expressed interest	Monitoring data; Qualitative data
Implementation	… can PLA-D be successfully delivered to intended participants?	Delivery of meetings; success or failure of PLA-D implementation; facilitators and barriers to implementation	Qualitative data; Direct observation
Practicality	… can PLA-D be carried out with intended participants using existing means, resources and circumstances	Solutions implemented; Positive/negative impacts on participants; Cost analysis	Monitoring data; Qualitative data

For peer review only

Figure 1: The PLA-D group cycle has 11 meetings across 4 phases

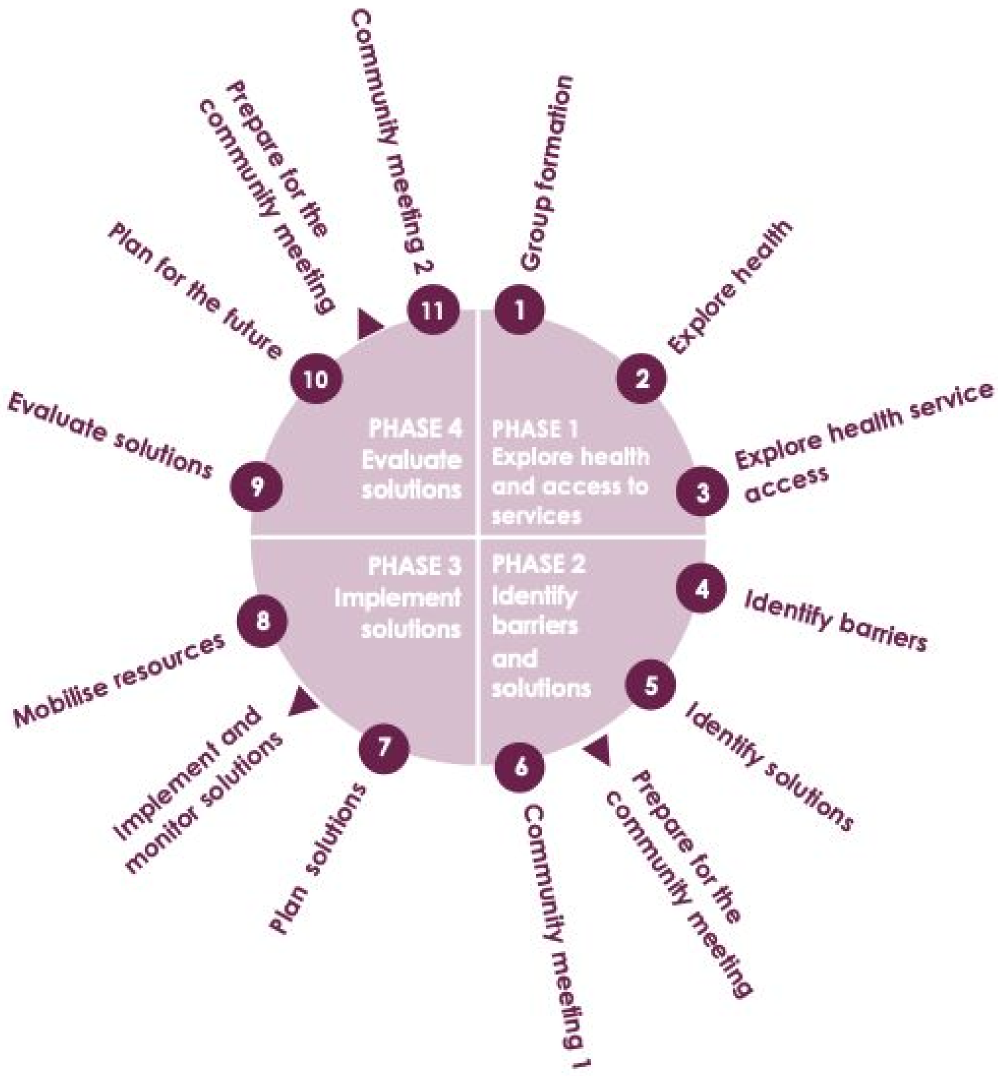

only

Figure 2: Decisions reached on design of PLA-D

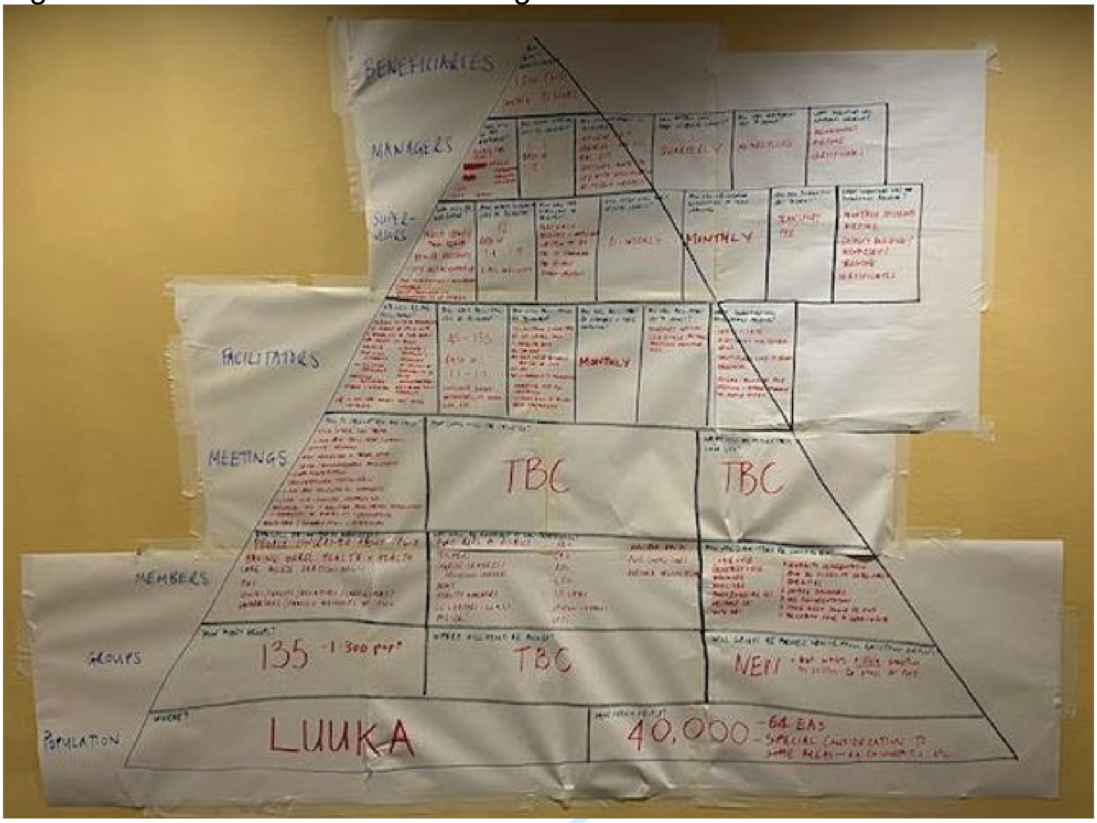

For peer review only

Figure 3: PLA-D Theory of Change

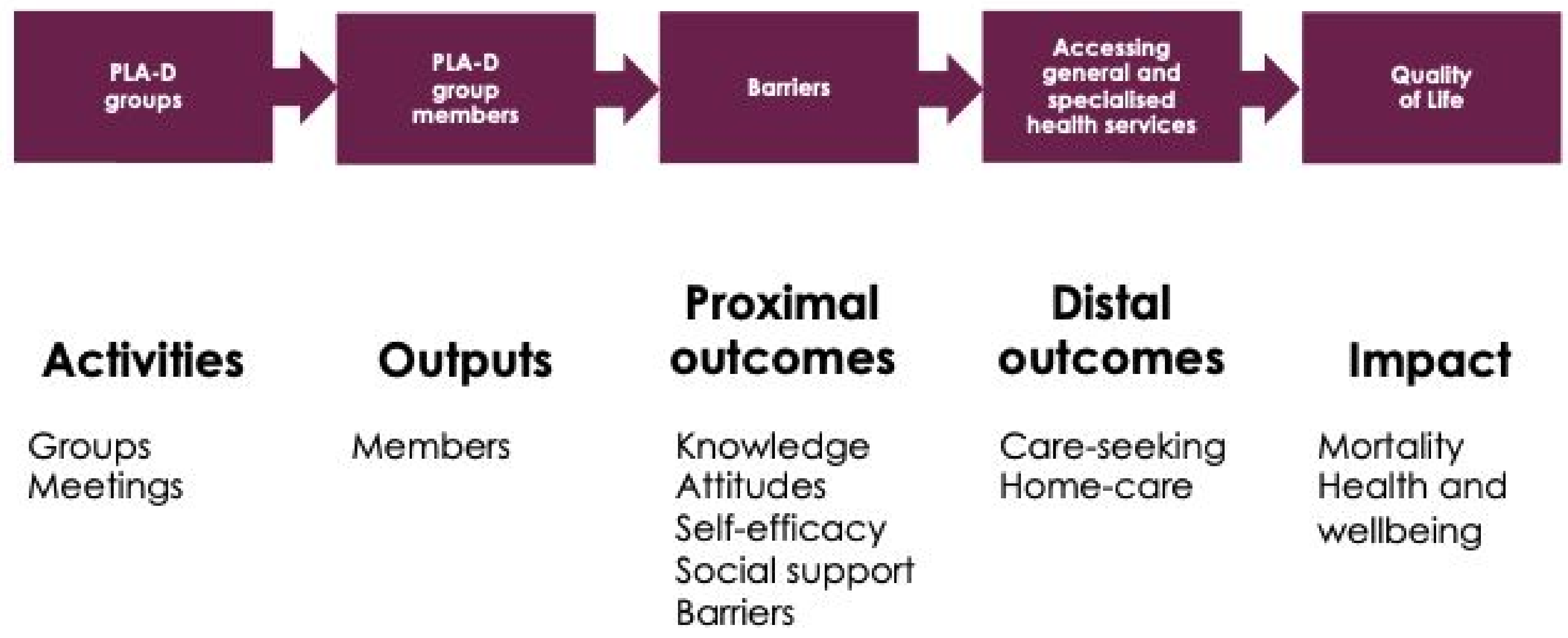

1

MRC/UVRI & LSHTM Uganda Research Unit **and LSHTM**

Questionnaire: Child version (aged 5-17)

Table of contents

Section 100: Respondent and household characteristics
Section 200: Washington Group Short Set Enhanced
Section 300: Quality of life
Section 400: Health condition and nutrition
Section 500: Health care utilization
Section 600: Participation
Section 700: Attitudes
Section 800: PLA-D specific questions

Nr.		
001	LOCATION ID NUMBER	______________________
002	**ID NUMBER**	______________________
003	**INTERVIEW TYPE**	1=Person with disabilities (direct)<br>2=Proxy for person with disabilities (unable to communicate/understand)<br>2=Proxy for person with disabilities (child<18)<br>______________________
004	**Interviewer 1 initials and code**	
005	**Date of interview**	__ __ 2023<br>(DAY MONTH)
006	**Time start interview**	__ hrs __ min
007	**Time end interview**	__ hrs __ min

For peer review only - http://bmjopen.bmj.com/site/about/guidelines.xhtml

SECTION 100 - RESPONDENT AND HOUSEHOLD CHARACTERISTICS (WOPS)

I am going to start by asking you some questions about your child and his/her living situation.

101	SEX	1= Male<br>2= Female
102	How old is [NAME] now?	Years……..
103	In what year was [NAME] born?<br>**IF KNOWN INCLUDE DATE OF BIRTH; CORRECT INCONSISTENCIES WITH AGE**	Year of birth….
		Date of birth<br>…../……/……….
104	Has [NAME] ever attended school or any early childhood education programme	1=Yes<br>2=No
105	What is the highest grade of school [NAME] has ever attended?	1=Early childhood education<br>2=Primary<br>3=Lower secondary<br>4=Upper secondary<br>5=Higher
106	At any time during the current school year did [NAME] attend school of any early childhood education programme?	1=Yes<br>2=No
107	During this current school year, what grade of school is [NAME] attending?	1=Early childhood education<br>2=Primary<br>3=Lower secondary<br>4=Upper secondary<br>5=Higher
108	What is the occupation of your head of household? Any other?<br>**TICK ALL THAT APPLY**	House work
		Farming
		Fishing
		Labouring (e.g. clearing streets, weeding)
		Tailoring
		Teacher / nurse
		Government administration
		Shop attendant
		other,
109	What is head of	1= No formal education

For peer review only - http://bmjopen.bmj.com/site/about/guidelines.xhtml

3

	household's highest level of education?	2= Less than primary (incomplete) school<br>3= Completed primary school<br>4= complete O level<br>5= Completed A level<br>6= Higher education than secondary school (diploma course or BTVET)<br>7= Adult education only<br>8= College/ University or more<br>88= Don't know
110	What is the total number of people who live in your household?	☐☐<br>Persons

LIVING CONDITIONS

111	What is the predominant material used for roofing?	1= Thatch<br>2= Tile<br>3= Iron sheets<br>4= Mixed iron/tile<br>5= Other
112	What is the state of the roof?	1= Very Good<br>1= Good<br>2= Moderate<br>3= Bad<br>4= Very bad
113	What are the walls of the house made of?	1= Burned brick<br>2= Unburned brick<br>3= Mud
114	What is the house floor made of?	1= Mud<br>2= Cement<br>3= Tiles
115	Who owns the house?	1= Self<br>2= Spouse<br>3= Respondent's children<br>4= Someone else in the household<br>5= Renting (landlord)<br>87= Other

For peer review only

4

SECTION 200: Washington Group Child Functioning Set

Commented [HK1]: REPLACE WITH CFM

I would like to ask you some questions about difficulties [NAME] may have

201	Does [NAME] wear glasses or contact lenses?	1=Yes<br>2=No	No – skip to 203
202	When wearing his/her glasses, does [NAME] have difficulty seeing? Would you say… [read response categories]	1=No difficulty<br>2=Some difficulty<br>3= A lot of difficulty<br>4= Cannot do	
203	Does [NAME] have difficulty seeing? Would you say… [read response categories]	1=No difficulty<br>2=Some difficulty<br>3= A lot of difficulty<br>4= Cannot do	
204	Does [NAME] use a hearing aid?	1=Yes<br>2=No	No – skip to 206
205	When using his/her hearing aid, does [NAME] have difficulty hearing sounds like people's voices or music? Would you say… [read response categories]	1=No difficulty<br>2=Some difficulty<br>3= A lot of difficulty<br>4= Cannot do	
206	Does [NAME] have difficulty hearing sounds like people's voices or music? Would you say… [read response categories]	1=No difficulty<br>2=Some difficulty<br>3= A lot of difficulty<br>4= Cannot do	
207	Does [NAME] use any equipment or receive assistance for walking?	1=Yes<br>2=No	No – skip to 212
208	Without his/her equipment or assistance, does [NAME] have difficulty walking 100 yards/meters on level ground? That would be about the length of 1 football field. Would you say… [read response categories]	1=No difficulty<br>2=Some difficulty<br>3= A lot of difficulty<br>4= Cannot do	
209	Without (his/her) equipment or assistance, does [NAME] have difficulty walking 500 meters/yards on level ground?<br>That would be about the length of 5 football fields.	1=No difficulty<br>2=Some difficulty<br>3= A lot of difficulty<br>4= Cannot do	
210	With (his/her) equipment or assistance, does [NAME] have difficulty walking 100 meters/yards on level ground? That would be about the length of 1 football field.	1=No difficulty<br>2=Some difficulty<br>3= A lot of difficulty<br>4= Cannot do	
211	With (his/her) equipment or assistance, does [NAME] have difficulty walking 500 meters/yards on level ground?<br>That would be about the length of 5 football fields.	1=No difficulty<br>2=Some difficulty<br>3= A lot of difficulty<br>4= Cannot do	
212	Compared with children of the same age, does [NAME] have difficulty walking 100 meters/yards	1=No difficulty<br>2=Some difficulty	

For peer review only

5

	on level ground?<br>That would be about the length of 1 football field.	3= A lot of difficulty<br>4= Cannot do	
213	Compared with children of the same age, does [NAME] have difficulty walking 500 meters/yards on level ground? That would be about the length of 5 football fields.	1=No difficulty<br>2=Some difficulty<br>3= A lot of difficulty<br>4= Cannot do	
214	Does [NAME] have difficulty with self-care such as feeding or dressing (himself/herself)?	1=No difficulty<br>2=Some difficulty<br>3= A lot of difficulty<br>4= Cannot do	
215	When **[NAME]** speaks, does (he/she) have difficulty being understood by people inside of this household?	1=No difficulty<br>2=Some difficulty<br>3= A lot of difficulty<br>4= Cannot do	
216	When [NAME] speaks, does (he/she) have difficulty being understood by people outside of this household?	1=No difficulty<br>2=Some difficulty<br>3= A lot of difficulty<br>4= Cannot do	
217	Compared with children of the same age, does [NAME] have difficulty learning things?	1=No difficulty<br>2=Some difficulty<br>3= A lot of difficulty<br>4= Cannot do	
218	Compared with children of the same age, does [NAME] have difficulty remembering things?	1=No difficulty<br>2=Some difficulty<br>3= A lot of difficulty<br>4= Cannot do	
219	Does [NAME] have difficulty concentrating on an activity that (he/she) enjoys doing?	1=No difficulty<br>2=Some difficulty<br>3= A lot of difficulty<br>4= Cannot do	
220	Does [NAME] have difficulty accepting changes in (his/her) routine?	1=No difficulty<br>2=Some difficulty<br>3= A lot of difficulty<br>4= Cannot do	
221	Compared with children of the same age, does [NAME] have difficulty controlling (his/her) behaviour?	1=No difficulty<br>2=Some difficulty<br>3= A lot of difficulty<br>4= Cannot do	
222	Does [NAME] have difficulty making friends?	1=No difficulty<br>2=Some difficulty<br>3= A lot of difficulty<br>4= Cannot do	
	The next questions have different options for answers. I am going to read these to you after each question.		

For peer review only

6

223	I would like to know how often [NAME] seems very anxious, nervous or worried.<br><br>Would you say: daily, weekly, monthly, a few times a year or never?	1=Daily<br>2=Weekly<br>3=Monthly<br>4=A few times a year<br>5=Never	
224	I would also like to know how often [NAME] seems very sad or depressed.<br><br>Would you say: daily, weekly, monthly, a few times a year or never?	1=Daily<br>2=Weekly<br>3=Monthly<br>4=A few times a year<br>5=Never	

6

For peer review only

7

SECTION 300 QUALITY OF LIFE (KIDSCREEN-PARENT)

301	In general, how would you say your child's health is?	1=Poor<br>2=Fair<br>3=Good<br>4=Very good<br>5=Excellent
	Over the past 2 weeks….	
302	Has your child felt fit and well?	1=Not at all<br>2=Slightly<br>3=Moderately<br>4=Very<br>5=Extremely
303	Has your child felt full of energy?	1=Never<br>2=Seldom<br>3=Quite often<br>4=Very often<br>5=Always
304	Has your child felt sad?	1=Never<br>2=Seldom<br>3=Quite often<br>4=Very often<br>5=Always
305	Has your child felt lonely?	1=Never<br>2=Seldom<br>3=Quite often<br>4=Very often<br>5=Always
306	Has your child had enough time for him/herself?	1=Never<br>2=Seldom<br>3=Quite often<br>4=Very often<br>5=Always
307	Has your child been able to do the things he/she wants to do in his/her free time?	1=Never<br>2=Seldom<br>3=Quite often<br>4=Very often<br>5=Always

For peer review only - http://bmjopen.bmj.com/site/about/guidelines.xhtml

8

308	Has your child felt that his/her parent(s) treated him/her fairly?	1=Never<br>2=Seldom<br>3=Quite often<br>4=Very often<br>5=Always
309	Has your child had fun with his/her friends?	1=Never<br>2=Seldom<br>3=Quite often<br>4=Very often<br>5=Always
310	Has your child been able to pay attention?	1=Never<br>2=Seldom<br>3=Quite often<br>4=Very often<br>5=Always
311	Has your child got on well at school?	1=Not at all<br>2=Slightly<br>3=Moderately<br>4=Very<br>5=Extremely

For peer review only

SECTION 400: HEALTH CONDITIONS AND NUTRITION
I am now going to ask some questions about your child's health

Nutrition (WOPS)

402	How many servings of fruit does [NAME] eat on a typical day?	Servings
403	How many servings of vegetables does [NAME]eat on a typical day?	Servings
404	In the last 12 months, did [NAME] ever hungry, but didn't eat because you couldn't afford enough food?	1 = Yes<br>2 = No
405	In the last 12 months, how often did [NAME] eat less than you felt [NAME] should because there wasn't enough food?	1= every month<br>2= almost every month<br>3= some months. But not every month<br>4= only in 1 or 2 months<br>5= Never

HEALTH STATUS (Model disability survey)

	**Has [NAME] been told by a doctor or health professional that he/she has…**		**If yes, has [NAME] been given medicine or treatment in the last 6-12 months?**
**406**	Vision loss	1 = Yes<br>2 = No	
**407**	Hearing loss	1 = Yes<br>2 = No	
**408**	High blood pressure	1 = Yes<br>2 = No	
**409**	Diabetes	1 = Yes<br>2 = No	
**410**	Arthritis	1 = Yes<br>2 = No	
**411**	Heart disease, coronary disease, heart attack	1 = Yes<br>2 = No	
**412**	Chronic bronchitis or emphysema	1 = Yes<br>2 = No	
**413**	Asthma, allergic, respiratory disease	1 = Yes<br>2 = No	
**414**	Back pain or disc problems	1 = Yes<br>2 = No	
**415**	Migraine (recurrent headaches)	1 = Yes	

For peer review only

For peer review only - http://bmjopen.bmj.com/site/about/guidelines.xhtml

10

		2 = No	
**416**	Stroke	1 = Yes<br>2 = No	
**417**	Depression or anxiety	1 = Yes<br>2 = No	
**418**	Gastritis or ulcer	1 = Yes<br>2 = No	
**419**	Tumour or cancer	1 = Yes<br>2 = No	
**420**	Trauma	1 = Yes<br>2 = No	
**421**	Dementia	1 = Yes<br>2 = No	
**422**	Kidney disease	1 = Yes<br>2 = No	
**423**	Skin disease	1 = Yes<br>2 = No	
**424**	Tuberculosis	1 = Yes<br>2 = No	
**425**	Mental (psychiatric) or behavioural disorders	1 = Yes<br>2 = No	

For peer review only

Section 500: Health care utilization

I would now like to know about your recent experiences with obtaining health care for your child from health care workers, hospitals, clinics and the health care system. I want to know if your child needed health care recently, and if so, why you needed health care provider you received care from.

501	In the last 2 years did your child need health care?	1=Yes<br>2=No
502	The last time your child needed health care, did you get health care?<br>**If "yes" Skip to 504**	1= Yes<br>2= No
**Interviewer: if respondent did not get health care, Q 502= "NO", ask Q 503A, 503a,503b and 503l then Skip to question 511**		
503A	**Interviewer: Respondent can select ONLY one main reason for visit. Use showcard**<br>**If respondent is having difficulties answering, you can read the categories**<br>What was the main reason your child needed care, even if your child did not get care?	
	1= Communicable diseases, infections, malaria, infection TB, HIV	
	2= Nutritional deficiencies	
	3= Acute conditions, (diarrhea, flu, headaches, fever, cough and others)	
	4= Injury (if FALLS record 4 and fill in FALLS in 403a (SPECIFY)	
	5= Surgery	
	6= Sleep problem	
	7= Occupational /work related condition/injury	
	8= Chronic pain in joints/arthritis (joints, back, neck)	
	9= Diabetes or related complications	
	10= Problems with heart including unexplained pain in chest	
	11= Problems with mouth, teeth, swallowing	
	12= Problems with breathing	
	13= High blood pressure, hypertension	

For peer review only - http://bmjopen.bmj.com/site/about/guidelines.xhtml

	14= Stroke/ sudden paralysis of one side of body	
	15= Generalized pain(stomach, muscle or other nonspecific pain)	
	16= Depression, anxiety	
	17= Cancer	
	87= Other	
503a	Other main reason SPECIFY	___________________________
503b	Which reason(s) best explains why your child did not get health care?<br><br>**Record all the numbers that respondent mentions spontaneously.**	
	1= could not afford the cost of the visit	
	2= no transport available	
	3= could not afford the cost of the transport	
	4= your child was previously badly treated	
	5= could not make time off work or had other commitments	
	6= the health care provider's drugs or equipment were inadequate	
	7= the health care provider's skills were inadequate	
	8= you did not know where to go	
	9= you tried but were denied health care	
	10= you thought your child was not sick enough	
	87= other	
503I	Other best reason why not get health care SPECIFY	______________________________<br>______________________________
504	Thinking about health care your child needed since last visit / in the last 2 years, where did your child go most often when he/she felt sick or needed to consult someone about health?	1= Private doctor's office<br>2= Private clinic or health centre<br>3= Government / public clinic or health centre<br>4= Government / public hospital<br>5= Charity or church run clinic

	**Interviewer: <u>Only one answer</u> allowed**	(name specific ones)
		6= Charity or church run hospital (name specific ones)
		7= Traditional healers / herbalist /shrine
		8= pharmacy
		9= Pastor or religious healer
		87= other
504a	How many visits do your child make to a health facility in a year?	Number ___________________
505	Did you have to pay fees for consultation and/or drugs?**If no skip to 507**	1= Yes<br>2= No
506	Who paid for the last visit?	Self (i.e. parent being interviewed)
		Spouse
		Son/daughter
		Other relative
		Other not relative
		Insurance
		was free

Interviewer ask Q 507, 508,509, and 510 ONLY when respondent use health services check Q 504
If not using health service skip to 511

For peer review only

14

507	When you visit the health facility, how long do you usually have to wait before it is your child's turn to be seen by a nurse or doctor?	1= Not long<br>2= Quite long<br>3= Long<br>4= Very long
508	When you visit the health facility, do the health professionals usually give you the time to explain to them what your child's health problem is?	1= Always<br>2= Most of the time<br>3= Sometimes<br>4= Not usually<br>5= Never
509	When you visit the health facility, do the health professionals usually take the time to explain your child's health problem and treatment in a way that you understand?	1= Always<br>2= Most of the time<br>3= Sometimes<br>4= Not usually<br>5= Never
509a	When you visit the health facility, do the health professionals usually take time to involve you/your child in making decisions for your treatment?[1]	1= Always<br>2= Most of the time<br>3= Sometimes<br>4= Not usually<br>5= Never
510	When you visit the health facility, is your child treated respectfully?	1= Always<br>2= Most of the time<br>3= Sometimes<br>4= Not usually<br>5= Never
511	Overall, are you satisfied with the services?	1= Very satisfied<br>2= Satisfied<br>3= Neither satisfied nor dissatisfied<br>4= Dissatisfied<br>5= Very dissatisfied
512	What are the reason(s) that people will go to the traditional healer for treatment?<br>**TICK ALL THAT APPLY**	Closer distance
		Traditional healer are cheaper
		Traditional healer allow you to pay in kind/ with goods
		Traditional healer will wait for payment
		Traditional healer is more knowledgeable about this

[1] Question added from Model Disability Survey

For peer review only - http://bmjopen.bmj.com/site/about/guidelines.xhtml

		problem
		Traditional healers give better treatment
		Decision made by caregiver or family
512a	Have you visited a traditional healer for treatment for your child?<br>**If no skip to 513**	1= yes<br>2= no
512b	During the last 12 months/one year, how often have you visited a traditional healer for treatment for your child?	1 = Once or twice<br>2 = Three to six times<br>3 = More than six times<br>8 = Don't know

Health centre/clinic, hospital stays

513	In the last two years has your child stayed an overnight in a health centre/ health clinic/hospital?<br>**IF NO SKIP TO 5600**	1= Yes<br>2= No
514	How many times has your child been hospitalized in the last 2 years?	Number of admissions<br>_____________
515	What type of hospital was it the last time your child was hospitalized? Public, private or church run?	1 = Public hospital<br>2 = Private hospital<br>3 = Charity or church run hospital<br>87 = Other Specify

For peer review only - http://bmjopen.bmj.com/site/about/guidelines.xhtml

16

**516** .	**RECORD REASON HOSPITALISATION**<br>**ONLY ONE REASON**<br>Which reason best describes why your child was last hospitalized?<br><br>1= Communicable diseases, infections, malaria, infection TB, HIV<br>2= Nutritional deficiencies<br>3= Acute conditions, (diarrhea, flu, headaches, fever, cough and others)<br>4= Injury (if FALLS record 4 and fill in FALLS in 403a (SPECIFY)<br>5= Surgery<br>6= Sleep problem<br>7= Occupational /work related condition/injury<br>8= Chronic pain in joints/arthritis (joints, back, neck)<br>9= Diabetes or related complications<br>10= Problems with heart including unexplained pain in chest<br>11= Problems with mouth, teeth, swallowing<br>12= Problems with breathing<br>13= High blood pressure, hypertension<br>14= Stroke/ sudden paralysis of one side of body<br>15= Generalized pain(stomach, muscle or other nonspecific pain)<br>16= Depression, anxiety<br>17= Cancer<br>87= Other	
516a	Other best reason child was hospitalized	____________
517	Who paid for this hospitalization?	Self<br>Spouse<br>Son/daughter<br>Other relative<br>Insurance<br>was free<br>Other
517a	Other paid hospitalization specify	
518	What was the outcome or result of your child's stay in the health centre/ health clinic/hospital? Did his/her condition…	1= Get much better<br>2= Get better<br>3= No change<br>4= Get worse<br>5= Get much worse
519	Was this the outcome or results you had expected?	1= yes<br>2= no

For peer review only - http://bmjopen.bmj.com/site/about/guidelines.xhtml

SECTION 600: Participation (CASP)

We are interested in finding out about the activities that your child participates in at home, school and in the community. You will be asked about your child's current level of participation with activities as compared to others their age. For each item, choose one of the following:

Full participation, your child participates in the activities the same as or more than others his/her age. [*With or without assistive devices or equipment*]

Somewhat limited, your child participates in the activities somewhat less than others his/her age. [*He/she may also need occasional supervision or assistance*]

Very limited, your child participates in the activities much less than others his/her age. [*He/she may also need a lot of supervision or assistance*]

Unable, your child cannot participate in the activities, although others his/her age do participate.

Not applicable, others your child's age would not be expected to participate in the activities.

	Compared to other children of your child's age, what is your child's current level of participation in the following activities<br><br>HOME PARTICIPATION	
601	Social, play or leisure activities with family members at home (e.g., games, hobbies, "hanging out")	1=Full participation<br>2=Somewhat limited<br>3=Very limited<br>4=Unable<br>5=Not applicable
602	Social, play or leisure activities with friends at home	1=Full participation<br>2=Somewhat limited<br>3=Very limited<br>4=Unable<br>5=Not applicable
603	Family chores, responsibilities and decisions at home (e.g., involvement in household chores and decisions about plans)**)**	1=Full participation<br>2=Somewhat limited<br>3=Very limited<br>4=Unable<br>5=Not applicable
604	Self-care activities (e.g. eating, dressing, bathing, combing or brushing hair, using the toilet)	1=Full participation<br>2=Somewhat limited<br>3=Very limited<br>4=Unable

For peer review only

		5=Not applicable
605	Moving about in and around the home	1=Full participation<br>2=Somewhat limited<br>3=Very limited<br>4=Unable<br>5=Not applicable
606	Communicating with others at home	1=Full participation<br>2=Somewhat limited<br>3=Very limited<br>4=Unable<br>5=Not applicable
	NEIGHBOURHOOD & COMMUNITY PARTICIPATION	
607	Social, play, or leisure activities with friends in the neighbourhood and community (e.g., casual games, “hanging out,” going to public places like the market, or church)	1=Full participation<br>2=Somewhat limited<br>3=Very limited<br>4=Unable<br>5=Not applicable
608	Structured events and activities in the neighbourhood and community (e.g. football, church or mosque, community meetings, and family functions)	1=Full participation<br>2=Somewhat limited<br>3=Very limited<br>4=Unable<br>5=Not applicable
609	Moving around the neighbourhood and community (e.g., public buildings, taxi’s, market) [*Please consider your child’s primary way of moving around, NOT your use of transportation*]	1=Full participation<br>2=Somewhat limited<br>3=Very limited<br>4=Unable<br>5=Not applicable
610	Communicating with others in the neighbourhood and community	1=Full participation<br>2=Somewhat limited<br>3=Very limited<br>4=Unable<br>5=Not applicable
	SCHOOL PARTICIPATION	
611	Educational (academic) activities with other pupils in your child’s classroom at school	1=Full participation<br>2=Somewhat limited<br>3=Very limited<br>4=Unable<br>5=Not applicable
612	Social, play and recreational activities with other pupils at school (e.g., “hanging out,” sports, creative arts, lunchtime or recess)	1=Full participation<br>2=Somewhat limited<br>3=Very limited<br>4=Unable<br>5=Not applicable
613	Moving around at school (e.g., to get to and use bathroom, playground, lunch area, library or other rooms and things that are available to other pupils your child’s age)	1=Full participation<br>2=Somewhat limited<br>3=Very limited

For peer review only

		4=Unable<br>5=Not applicable
614	Using educational materials and equipment that are available to other pupils in your child's classroom/s or that have been modified for your child (e.g., books, chairs and desks)	1=Full participation<br>2=Somewhat limited<br>3=Very limited<br>4=Unable<br>5=Not applicable
615	Communicating with other pupils and adults at school	1=Full participation<br>2=Somewhat limited<br>3=Very limited<br>4=Unable<br>5=Not applicable
	DAILY LIVING ACTIVITIES	
616	Household activities (e.g., preparing meals, doing laundry, washing dishes)	1=Full participation<br>2=Somewhat limited<br>3=Very limited<br>4=Unable<br>5=Not applicable
617	Shopping and managing money (e.g., shopping at market, figuring out correct change)	1=Full participation<br>2=Somewhat limited<br>3=Very limited<br>4=Unable<br>5=Not applicable
618	Managing daily schedule (e.g. doing and completing daily activities on time; getting ready for school and doing homework)	1=Full participation<br>2=Somewhat limited<br>3=Very limited<br>4=Unable<br>5=Not applicable
619	Using transportation to get around in the community (e.g. to and from school, work, social or leisure activities) [*using taxi and body*]	1=Full participation<br>2=Somewhat limited<br>3=Very limited<br>4=Unable<br>5=Not applicable
620	School activities and responsibilities (e.g., completion of tasks, punctuality, attendance and getting along with teachers)	1=Full participation<br>2=Somewhat limited<br>3=Very limited<br>4=Unable<br>5=Not applicable

For peer review only - http://bmjopen.bmj.com/site/about/guidelines.xhtml

SECTION 700: PLA-D

BASELINE only

Now I would like to ask you some questions about your needs and expectations of the programme

Nr.		CODE
701	What are the three biggest issues in [NAME]'s day-to-day life that you/your child find difficult or need support with?	
702	What are the three biggest issues related to [NAME]'s health you/your child find difficult or need support with?	
703	What are your main goals for attending this group?	

END-LINE only

Now I would like to ask you some questions about your feelings about the programme?

Remind the participant of their goals from the baseline

Nr.		CODE
804	Do you feel that you have achieved or met your goals?	1=Not at all<br>2= Partially<br>3= Fully
805	Please explain	
806	How satisfied were you with the content of the group programme?	1= Very dissatisfied<br>2= Disatisfied<br>3= Neither satisfied nor dissatisfied<br>4= Satisfied<br>5= Very satisfied
807	How satisfied were you with the organization of the programme (e.g. transport, location, refreshments)?	1= Very dissatisfied<br>2= Disatisfied<br>3= Neither satisfied nor dissatisfied<br>4= Satisfied<br>5= Very satisfied
808	How satisfied were you with the facilitators of the programme?	1= Very dissatisfied<br>2= Disatisfied<br>3= Neither satisfied nor dissatisfied<br>4= Satisfied<br>5= Very satisfied

For peer review only

For peer review only - http://bmjopen.bmj.com/site/about/guidelines.xhtml

809	Can you suggest ways in which the programme could be improved?	

810	**RECORD THE TIME**	___ Hrs __Min	TIMEPART2END

Closing statement and thank the respondent
INTERVIEWER: This is the end of the interview
This completes the interview. We thank you for your time and answers. I have your contact details and may be in touch again.

For peer review only

MRC/UVRI & LSHTM Uganda Research Unit **and LSHTM**

Questionnaire: Adult version

Table of contents

Section 100: Respondent and household characteristics
Section 200: Washington Group Short Set Enhanced
Section 300: Quality of life
Section 400: Health condition and nutrition
Section 500: Health care utilization
Section 600: Participation
Section 700: Attitudes
Section 800: PLA-D specific questions

Nr.		
001	LOCATION ID NUMBER	______________________
002	**ID NUMBER**	______________________
003	**INTERVIEW TYPE**	1=Person with disabilities (direct)<br>2=Proxy for person with disabilities (unable to communicate/understand)<br>2=Proxy for person with disabilities (child<18)<br>______________________
004	**Interviewer 1 initials and code**	
005	**Date of interview**	__ __ 2023<br>(DAY MONTH)
006	**Time start interview**	__ hrs __ min
007	**Time end interview**	__ hrs __ min

For peer review only - http://bmjopen.bmj.com/site/about/guidelines.xhtml

SECTION 100 - RESPONDENT AND HOUSEHOLD CHARACTERISTICS (WOPS)

I am going to start by asking you some questions about yourself and your living situation.

101	SEX	1= Male<br>2= Female
102	How old are you now?	Years……..
103	In what year were you born?<br>**IF KNOWN INCLUDE DATE OF BIRTH; CORRECT INCONSISTENCIES WITH AGE**	Year of birth….<br><br>Date of birth<br>…../……/……….
104	What is current marital status?	1= Single/ Never married<br>2= Married / Cohabitating<br>3= Divorced / Separated<br>4= Widowed
105	What is / was your occupation?<br>Any other?<br>**TICK ALL THAT APPLY**	House work
		Farming
		Fishing
		Labouring (e.g. clearing streets, weeding)
		Tailoring
		Teacher / nurse
		Government administration
		Shop attendant
		other,
105a	Other spouse occupation SPECIFY	____________________
106	What is / was your spouse's occupation? Any other?<br>**TICK ALL THAT APPLY**	House work
		Farming
		Fishing
		Labouring(e.g. clearing streets, weeding etc)
		Tailoring
		Teacher / nurse
		Government administration
		Shop attendant
		Other
106a	Other respondent occupation SPECIFY	____________________
107	Are you still working?	1= Yes, full-time<br>1a= Yes, part-time<br>1b= Yes, self-employed

For peer review only - http://bmjopen.bmj.com/site/about/guidelines.xhtml

		1c= No, retired<br>2= No
108	What is your highest level of education?	1= No formal education<br>2= Less than primary (incomplete) school<br>3= Completed primary school<br>4= complete O level<br>5= Completed A level<br>6= Higher education than secondary school (diploma course or BTVET)<br>7= Adult education only<br>8= College/ University or more<br>88= Don't know
109	What is the total number of people who live in your household?	__ __ Persons

LIVING CONDITIONS

110	What is the predominant material used for roofing?	1= Thatch<br>2= Tile<br>3= Iron sheets<br>4= Mixed iron/tile<br>5= Other
111	What is the state of the roof?	1= Very Good<br>1= Good<br>2= Moderate<br>3= Bad<br>4= Very bad
112	What are the walls of the house made of?	1= Burned brick<br>2= Unburned brick<br>3= Mud
113	What is the house floor made of?	1= Mud<br>2= Cement<br>3= Tiles
114	Who owns the house?	1= Self<br>2= Spouse<br>3= Respondent's children<br>4= Someone else in the household<br>5= Renting (landlord)<br>87= Other

For peer review only - http://bmjopen.bmj.com/site/about/guidelines.xhtml

SECTION 200: Washington Group Enhanced Set

**201**	Do you have difficulty seeing even when wearing your glasses/contact lenses? Would you say….	1= No difficulty<br>2= Some difficulty<br>3= A lot of difficulty<br>4= Cannot do at all
**202**	Do you have difficulty hearing, even when using a hearing aid(s)? Would you say…	1= No difficulty<br>2= Some difficulty<br>3= A lot of difficulty<br>4= Cannot do at all
**203**	Do you have difficulty walking or climbing steps? Would you say…	1= No difficulty<br>2= Some difficulty<br>3= A lot of difficulty<br>4= Cannot do at all
**204**	Using your usual languate do you have difficulty communicating, for example, understanding or behing understood? Would you say…	1= No difficulty<br>2= Some difficulty<br>3= A lot of difficulty<br>4= Cannot do at all
**205**	Do you have difficulty remembering or concentrating? Would you say…	1= No difficulty<br>2= Some difficulty<br>3= A lot of difficulty<br>4= Cannot do at all
**206**	Do you have difficulty with self care, such as washing all over or dressing? Would you say…	1= No difficulty<br>2= Some difficulty<br>3= A lot of difficulty<br>4= Cannot do at all
**207**	Do you have difficulty raising a 2 liter bottle of water or soda from the waist to eye level? Would you say…	1= No difficulty<br>2= Some difficulty<br>3= A lot of difficulty<br>4= Cannot do at all
**208**	Do you have difficulty using your hands and fingers, such as picking up small objects, for example, a button or pencil, or opening or closing containers or bottles? Would you say…	1= No difficulty<br>2= Some difficulty<br>3= A lot of difficulty<br>4= Cannot do at all
**209**	How often do you feel worried, nervous or anxious? Would you say…	1= Daily<br>2= Weekly<br>3= Monthly<br>4= A few times a year<br>5= Never
	Thinking about the last time you felt worried, nervous or anxious, how would you describe the level of these feelings. Would you say…	1= A little<br>2= A lot<br>3= Somewhere between a little and a lot
**209**	How often do you feel depressed? Would you say…	1= Daily<br>2= Weekly<br>3= Monthly<br>4= A few times a year

For peer review only - http://bmjopen.bmj.com/site/about/guidelines.xhtml

		5= Never
**210**	Thinking about the last time you felt depressed, how would you describe the level of these feelings. Would you say…	1= A little<br>2= A lot<br>3= Somewhere between a little and a lot
**211**	At what age did you first start experiencing these difficulties	_____ years<br>0 if age<1<br>999 if don't know

For peer review only

For peer review only - http://bmjopen.bmj.com/site/about/guidelines.xhtml

SECTION 300 QUALITY OF LIFE (WHO-QOL BREF)

This assessment asks how you feel about your quality of life, health, or other areas of your life. Please answer all the questions. If you are unsure about which response to give to a question, please choose the one that appears most appropriate. This can often be your first response. Please keep in mind your standards, hopes, pleasures and concerns. We ask that you think about your life in the last two weeks.

301	How would you rate your quality of life?	1= Very poor<br>2= Poor<br>3= Neither poor nor good<br>4= Good<br>5= Very good
302	How satisfied are you with your health?	1= Very dissatisfied<br>2= Disatisfied<br>3= Neither satisfied nor dissatisfied<br>4= Satisfied<br>5= Very satisfied
303	To what extent do you feel that physical pain prevents you from doing what you need to do?	1= Not at all<br>2= A little<br>3= A moderate amount<br>4= Very much<br>5= An extreme amount
304	How much do you need any medical treatment to function in your daily life?	1= Not at all<br>2= A little<br>3= A moderate amount<br>4= Very much<br>5= An extreme amount
305	How much do you enjoy life?	1= Not at all<br>2= A little<br>3= A moderate amount<br>4= Very much<br>5= An extreme amount
306	To what extent do you feel your life to be meaningful?	1= Not at all<br>2= A little<br>3= A moderate amount<br>4= Very much<br>5= An extreme amount

For peer review only

307	How well are you able to concentrate?	1= Not at all<br>2= A little<br>3= A moderate amount<br>4= Very much<br>5= Extremely
308	How safe do you feel in your daily life?	1= Not at all<br>2= A little<br>3= A moderate amount<br>4= Very much<br>5= Extremely
309	How healthy is your physical environment?	1= Not at all<br>2= A little<br>3= A moderate amount<br>4= Very much<br>5= Extremely
310	Do you have enough energy for your everday activities?	1= Not at all<br>2= A little<br>3= Moderately<br>4= Mostly<br>5= Completely
311	Are you able to accept your bodily appearance?	1= Not at all<br>2= A little<br>3= Moderately<br>4= Mostly<br>5= Completely
312	Have you enough money to meet your needs?	1= Not at all<br>2= A little<br>3= Moderately<br>4= Mostly<br>5= Completely
313	How available to you is the information that you need in your day-to-day life?	1= Not at all<br>2= A little<br>3= Moderately<br>4= Mostly<br>5= Completely
314	To what extent do you have the opportunity for leisure activities?	1= Not at all<br>2= A little<br>3= Moderately<br>4= Mostly<br>5= Completely

For peer review only - http://bmjopen.bmj.com/site/about/guidelines.xhtml

315	How well are you able to get around?	1=Very poor<br>2= Poor<br>3= Neither poor nor good<br>4= Good<br>5= Very good
316	How satisfied are you with your sleep?	1= Very dissatisfied<br>2= Disatisfied<br>3= Neither satisfied nor dissatisfied<br>4= Satisfied<br>5= Very satisfied
317	How satisfied are you with your ability to perform your daily activities?	1= Very dissatisfied<br>2= Disatisfied<br>3= Neither satisfied nor dissatisfied<br>4= Satisfied<br>5= Very satisfied
318	How satisfied are you with your capacity for work?	1= Very dissatisfied<br>2= Disatisfied<br>3= Neither satisfied nor dissatisfied<br>4= Satisfied<br>5= Very satisfied
319	How satisfied are you with yourself?	1= Very dissatisfied<br>2= Disatisfied<br>3= Neither satisfied nor dissatisfied<br>4= Satisfied<br>5= Very satisfied
320	How satisfied are you with your personal relationships?	1= Very dissatisfied<br>2= Disatisfied<br>3= Neither satisfied nor dissatisfied<br>4= Satisfied<br>5= Very satisfied
321	How satisfied are you with your sex life?	1= Very dissatisfied<br>2= Disatisfied<br>3= Neither satisfied nor dissatisfied<br>4= Satisfied<br>5= Very satisfied
322	How satisfied are you with the support you get from your friends?	1= Very dissatisfied<br>2= Disatisfied<br>3= Neither satisfied nor dissatisfied<br>4= Satisfied<br>5= Very satisfied

For peer review only - http://bmjopen.bmj.com/site/about/guidelines.xhtml

For peer review only

323	How satisfied are you with the conditions of your living place?	1= Very dissatisfied<br>2= Disatisfied<br>3= Neither satisfied nor dissatisfied<br>4= Satisfied<br>5= Very satisfied
324	How satisfied are you with your access to health services?	1= Very dissatisfied<br>2= Disatisfied<br>3= Neither satisfied nor dissatisfied<br>4= Satisfied<br>5= Very satisfied
325	How satisfied are you with your transport?	1= Very dissatisfied<br>2= Disatisfied<br>3= Neither satisfied nor dissatisfied<br>4= Satisfied<br>5= Very satisfied
326	How often do you have negative feelings such as blue mood, depair, anxiety or depression?	1= Never<br>2= Infrequently<br>3= Sometimes<br>4= Frequently<br>5= Always

For peer review only

SECTION 400: HEALTH CONDITIONS AND NUTRITION

I am now going to ask some questions about your health

Self-rated health

401	In general, how would you rate your health?	1= Good<br>2= Rather good<br>3= Poor<br>4= Very poor

Nutrition (WOPS)

402	How many servings of <u>fruit</u> do you eat on a typical day?	Servings
403	How many servings of <u>vegetables</u> do you eat on a typical day?	Servings
404	In the <u>last 12 months</u>, were you ever hungry, but didn't eat because you couldn't afford enough food?	1 = Yes<br>2 = No
405	In the <u>last 12 months</u>, how often did you eat less than you felt you should because there wasn't enough food?	1= every month<br>2= almost every month<br>3= some months. But not every month<br>4= only in 1 or 2 months<br>5= Never

HEALTH STATUS (Model disability survey)

	**Have you been told by a doctor or health professional that you have…**		**If yes, have you been given medicine or treatment in the last 6-12 months?**
**406**	Vision loss	1 = Yes<br>2 = No	
**407**	Hearing loss	1 = Yes<br>2 = No	
**408**	High blood pressure	1 = Yes<br>2 = No	
**409**	Diabetes	1 = Yes<br>2 = No	
**410**	Arthritis	1 = Yes<br>2 = No	
**411**	Heart disease, coronary disease, heart attack	1 = Yes<br>2 = No	
**412**	Chronic bronchitis or emphysema	1 = Yes	

For peer review only - http://bmjopen.bmj.com/site/about/guidelines.xhtml

		2 = No	
**413**	Asthma, allergic, respiratory disease	1 = Yes<br>2 = No	
**414**	Back pain or disc problems	1 = Yes<br>2 = No	
**415**	Migraine (recurrent headaches)	1 = Yes<br>2 = No	
**416**	Stroke	1 = Yes<br>2 = No	
**417**	Depression or anxiety	1 = Yes<br>2 = No	
**418**	Gastritis or ulcer	1 = Yes<br>2 = No	
**419**	Tumour or cancer	1 = Yes<br>2 = No	
**420**	Trauma	1 = Yes<br>2 = No	
**421**	Dementia	1 = Yes<br>2 = No	
**422**	Kidney disease	1 = Yes<br>2 = No	
**423**	Skin disease	1 = Yes<br>2 = No	
**424**	Tuberculosis	1 = Yes<br>2 = No	
**425**	Mental (psychiatric) or behavioural disorders	1 = Yes<br>2 = No	

For peer review only

Section 500: Health care utilization

I would now like to know about your recent experiences with obtaining health care from health care workers, hospitals, clinics and the health care system. I want to know if you needed health care recently, and if so, why you needed health care provider youreceived care from.

501	In the last 2 years did you need health care?	1=Yes<br>2=No
502	The last time you needed health care, did you get health care?<br>**If "yes" Skip to 504**	1= Yes<br>2= No
**Interviewer: if respondent did not get health care, Q 502= "NO", ask Q 503A, 503a,503b and 503I then Skip to question 511**		
503A	**Interviewer: Respondent can select ONLY one <u>main reason</u> for visit. Use showcard**<br>**If respondent is having difficulties answering, you can read the categories**<br>What was the main reason you needed care, even if you did not get care?	
	1= Communicable diseases, infections, malaria, infection TB, HIV	
	2= Nutritional deficiencies	
	3= Acute conditions, (diarrhea, flu, headaches, fever, cough and others)	
	4= Injury (if FALLS record 4 and fill in FALLS in 403a (SPECIFY)	
	5= Surgery	
	6= Sleep problem	
	7= Occupational /work related condition/injury	
	8= Chronic pain in joints/arthritis (joints, back, neck)	
	9= Diabetes or related complications	
	10= Problems with heart including unexplained pain in chest	
	11= Problems with mouth, teeth, swallowing	
	12= Problems with breathing	
	13= High blood pressure, hypertension	
	14= Stroke/ sudden paralysis of one side of body	

For peer review only - http://bmjopen.bmj.com/site/about/guidelines.xhtml

	15= Generalized pain(stomach, muscle or other nonspecific pain)	
	16= Depression, anxiety	
	17= Cancer	
	87= Other	
503a	Other main reason SPECIFY	___________________________
503b	Which reason(s) best explains why you did not get health care?<br><br>**Record all the numbers that respondent mentions spontaneously.**	
	1= could not afford the cost of the visit	
	2= no transport available	
	3= could not afford the cost of the transport	
	4= you were previously badly treated	
	5= could not make time off work or had other commitments	
	6= the health care provider's drugs or equipment were inadequate	
	7= the health care provider's skills were inadequate	
	8= you did not know where to go	
	9= you tried but were denied health care	
	10= you thought you were not sick enough	
	87= other	
503I	Other best reason why not get health care SPECIFY	___________________________<br>___________________________
504	Thinking about health care you needed since last visit / in the last 2 years, where did you go most often when you felt sick or needed to consult someone about your health?	1= Private doctor's office<br>2= Private clinic or health centre<br>3= Government / public clinic or health centre<br>4= Government / public hospital<br>5= Charity or church run clinic

For peer review only - http://bmjopen.bmj.com/site/about/guidelines.xhtml

	**Interviewer: <u>Only one answer</u> allowed**	(name specific ones)
		6= Charity or church run hospital (name specific ones)
		7= Traditional healers / herbalist /shrine
		8= pharmacy
		9= Pastor or religious healer
		87= other
504a	How many visits do you make to a health facility in a year?	Number ___________________
505	Did you have to pay fees for consultation and/or drugs?**If no skip to 507**	1= Yes<br>2= No
506	Who paid for the last visit?	Self
		Spouse
		Son/daughter
		Other relative
		Other not relative
		Insurance
		was free

Interviewer ask Q 507, 508,509, and 510 ONLY when respondent use health services check Q 504
If not using health service skip to 511

For peer review only - http://bmjopen.bmj.com/site/about/guidelines.xhtml

507	When you visit the health facility, how long do you usually have to wait before it is your turn to be seen by a nurse or doctor?	1= Not long<br>2= Quite long<br>3= Long<br>4= Very long
508	When you visit the health facility, do the health professionals usually give you the time to explain to them what your health problem is?	1= Always<br>2= Most of the time<br>3= Sometimes<br>4= Not usually<br>5= Never
509	When you visit the health facility, do the health professionals usually take the time to explain your health problem and treatment in a way that you understand?	1= Always<br>2= Most of the time<br>3= Sometimes<br>4= Not usually<br>5= Never
509a	When you visit the health facility, do the health professionals usually take time to involve you in making decisions for your treatment?[1]	1= Always<br>2= Most of the time<br>3= Sometimes<br>4= Not usually<br>5= Never
510	When you visit the health facility, are you treated respectfully?	1= Always<br>2= Most of the time<br>3= Sometimes<br>4= Not usually<br>5= Never
511	Overall, are you satisfied with the services?	1= Very satisfied<br>2= Satisfied<br>3= Neither satisfied nor dissatisfied<br>4= Dissatisfied<br>5= Very dissatisfied
512	What are the reason(s) that people will go to the traditional healer for treatment?<br>**TICK ALL THAT APPLY**	Closer distance
		Traditional healer are cheaper
		Traditional healer allow you to pay in kind/ with goods
		Traditional healer will wait for payment
		Traditional healer is more knowledgeable about this

[1] Question added from Model Disability Survey

For peer review only - http://bmjopen.bmj.com/site/about/guidelines.xhtml

		problem
		Traditional healers give better treatment
		Decision made by caregiver or family
512a	Have you visited a traditional healer for treatment?<br>**If no skip to 513**	1= yes<br>2= no
512b	During the last 12 months/one year, how often have you visited a traditional healer?	1 = Once or twice<br>2 = Three to six times<br>3 = More than six times<br>8 = Don't know

Health centre/clinic, hospital stays

513	In the last two years have you stayed an overnight in a health centre/ health clinic/hospital?<br>**IF NO SKIP TO 5600**	1= Yes<br>2= No
514	How many times have you been hospitalized in the last 2 years?	Number of admissions<br>_____________
515	What type of hospital was it the last time you were hospitalized? Public, private or church run?	1 = Public hospital<br>2 = Private hospital<br>3 = Charity or church run hospital<br>87 = Other Specify
**516** .	**RECORD REASON HOSPITALISATION**<br>**ONLY ONE REASON**<br>Which reason best describes why you were last hospitalized?<br><br>1= Communicable diseases, infections, malaria, infection TB, HIV<br>2= Nutritional deficiencies<br>3= Acute conditions, (diarrhea, flu, headaches, fever, cough and others)<br>4= Injury (if FALLS record 4 and fill in FALLS in 403a (SPECIFY)<br>5= Surgery<br>6= Sleep problem<br>7= Occupational /work related condition/injury<br>8= Chronic pain in joints/arthritis (joints, back, neck)<br>9= Diabetes or related complications<br>10= Problems with heart including unexplained pain in chest<br>11= Problems with mouth, teeth, swallowing<br>12= Problems with breathing<br>13= High blood pressure, hypertension<br>14= Stroke/ sudden paralysis of one side of body	

For peer review only

	15= Generalized pain(stomach, muscle or other nonspecific pain)<br>16= Depression, anxiety<br>17= Cancer<br>87= Other	
516a	Other best reason respondent was hospitalized	_______________
517	Who paid for this hospitalization?	Self<br>Spouse<br>Son/daughter<br>Other relative<br>Insurance<br>was free<br>Other
517a	Other paid hospitalization specify	
518	What was the outcome or result of your stay in the health centre/ health clinic/hospital? Did your condition…	1= Get much better<br>2= Get better<br>3= No change<br>4= Get worse<br>5= Get much worse
519	Was this the outcome or results you had expected?	1= yes<br>2= no

For peer review only

For peer review only - http://bmjopen.bmj.com/site/about/guidelines.xhtml

SECTION 600: Participation (SINTEF)

I'm going to ask you some questions about your involvement in different aspects of family, social life and society. Please listen to each one and answer yes, no, sometimes, sometimes or not applicable.

Q. #	Question	Codes					Go to Q
		Yes	No	Sometimes	N/A	Don't know	
601	Are you consulted about making household decisions?	1	2	3	4	5	
602	Do you go with the family to events such as family gatherings, weddings, thanksgivings, funerals and birthdays, and other social functions?	1	2	3	4	5	
603	Do you feel involved and part of the household or family?	1	2	3	4	5	
604	Does the family involve you in conversations?	1	2	3	4	5	
605	Does the family help you with daily activities/tasks?	1	2	3	4	5	"No, DK, N/A" →607
606	Do you appreciate it or like the fact that you get this help?	1	2	3	4	5	
607	Do you/did you take part in your own traditional practices (e.g initiation ceremonies)?	1	2	3	4	5	
608	Are you aware of organizations for people with disabilities (DPO)?	1	2				"No" →610
609	Are you a member of a DPO?	1	2				
610	Do you participate in local community meetings?	1	2	3	4	5	"No" →612
611	Do you feel your voice is being heard?	1	2			5	
612	Did you vote in the last election?	1	2			5	**"No"→ sect 700**
613	Was it related to your disability that you didn't vote?	1	2			5	

For peer review only - http://bmjopen.bmj.com/site/about/guidelines.xhtml

Section 700: Attitudes (Model Disability Survey)

Now I want to ask you some questions about the attitudes of people around you. When answering these questions please tell me on a scale from 1 to 5, where 1 is not at all and 5 means completely**.**

Q. #	Question	Codes					
		1 Not at all	2	3	4	5 Yes, complete ly	98 Not applicable
701	Do you have problems getting involved in society because of the attitudes of others?	1	2	3	4	5	98
702	Do you feel that some people treat you unfairly?	1	2	3	4	5	98
703	Do you make your own choices about your day-to-day life? For example, where you go, what to eat.	1	2	3	4	5	98
704	Do you feel other people accept you?	1	2	3	4	5	98
705	Do you feel that other people respect you? For example, do you feel that others value you as a person and listen to what you have to say?	1	2	3	4	5	98
706	Do you consider yourself a burden on society?	1	2	3	4	5	98
707	Do people around you tend to become impatient with you?	1	2	3	4	5	98
708	Do people around you not expect much from you?	1	2	3	4	5	98
709	Is living with dignity a problem for you because of the attitudes and actions of others?	1	2	3	4	5	98

For peer review only

For peer review only - http://bmjopen.bmj.com/site/about/guidelines.xhtml

SECTION 800: PLA-D

BASELINE only

Now I would like to ask you some questions about your needs and expectations of the programme

Nr.		CODE
801	What are the three biggest issues that in your day-to-day life that you find difficult or need support with?	
802	What are the three biggest issues related to your health you find difficult or need support with?	
803	What are your main goals for attending this group?	

END-LINE only

Now I would like to ask you some questions about your feelings about the programme?

Remind the participant of their goals from the baseline

Nr.		CODE
804	Do you feel that you have achieved or met your goals?	1=Not at all<br>2= Partially<br>3= Fully
805	Please explain	
806	How satisfied were you with the content of the group programme?	1= Very dissatisfied<br>2= Disatisfied<br>3= Neither satisfied nor dissatisfied<br>4= Satisfied<br>5= Very satisfied
807	How satisfied were you with the organization of the programme (e.g. transport, location, refreshments)?	1= Very dissatisfied<br>2= Disatisfied<br>3= Neither satisfied nor dissatisfied<br>4= Satisfied<br>5= Very satisfied
808	How satisfied were you with the facilitators of the programme?	1= Very dissatisfied<br>2= Disatisfied<br>3= Neither satisfied nor dissatisfied<br>4= Satisfied<br>5= Very satisfied
809	Can you suggest ways in which the	

For peer review only - http://bmjopen.bmj.com/site/about/guidelines.xhtml

	programme could be improved?	

810	**RECORD THE TIME**	___ Hrs __Min	TIMEPART2END

Closing statement and thank the respondent
INTERVIEWER: This is the end of the interview
This completes the interview. We thank you for your time and answers. I have your contact details and may be in touch again.

For peer review only

For peer review only - http://bmjopen.bmj.com/site/about/guidelines.xhtml

Focus Group Discussion for Participants of PLA-D

Pilot-testing a participatory approach to improve access to healthcare for people with disabilities in Uganda

Objective: To gather information to help further design a participatory approach for people with disabilities to improve health

These questions should be used to guide discussion but do not have to be used in the sequence listed below. The interviewer should follow up on any additional issues that may arise and seem important in relation to the issues above.

Introduction

- Greet them and thank them for their time
- Identify yourself by name and organisation.
- Read out the information sheet. Remind them of confidentiality and anonymity. Check if they have any questions. Remind them that they are free to decline to answer any of the questions or stop the interview at any time.
- Record their consent/assent in the relevant form OR record verbal consent.
- Start recording

Notes: the following details must be recorded in field notes

Participant Code	
Interview date and time	
Interview location or mode (phone, video)	
Interviewer	
Interviewee number	
Gender (number men/women)	
Age (number in broad groups)	
General observations (anything which might impact how the interview is conducted)	

As we have discussed, we are gathering information to help improve the design of the groups about disability that you recently participated in.

1. Share with me your experience participating in the PLA-D groups?
2. What do you think was the purpose of the groups?

3. What did you think about the way the groups were organised?

   Prompt – facilitation, regularity of meetings, location, group facilitator, frequency

4. Are there any sessions that were particularly interesting and important for you, and if so why?

5. What, if any, do you think were the main benefits of the groups?

6. What, if any, were the main problems with the groups?

Prompt – gender, age, different forms of disability, date, location and timing of meetings

7. If we were to plan the groups again, what could we do differently?

8. Is there anything else that you would like to say about the groups that we have not covered?

For peer review only

MRC/UVRI and LSHTM Uganda Research Unit

Medical Research Council

Uganda Virus Research Institute

LONDON SCHOOL of HYGIENE &TROPICAL MEDICINE

LONDON SCHOOL of HYGIENE &TROPICAL MEDICINE

Observation of PLA-D group

The Missing Billion: Pilot-testing PLA-D groups to improve access to healthcare for people with disabilities in Uganda

Objective: To gather information to help further design a PLA-D group

Introduction

- Greet them and thank them for their time
- Identify yourself by name and organisation.
- Read out the information sheet. Remind them of confidentiality and anonymity. Check if they have any questions.
- Record their consent/assent in the relevant form OR record verbal consent.

Notes: the following details must be recorded in field notes

Group Code	
Observation date and time	
Observation location	
Observer name	
Participant gender (number men/women)	
General observations (anything which might impact how the interview is conducted)	

Observe the session and record your observations on the following page:

For peer review only

MRC/UVRI and LSHTM Uganda Research Unit

Facilitation group checklist	Activity demonstrated	Observed		Comment
		Yes	No	
Logistics	The group was held at the date, time and location that was expected			
	Number of facilitators who attended was the same as expected			
	Number of participants who attended were the same as expected			
	Group was conducted and ran according to schedule			
Environment created by facilitator	Room/location set up was in a circular pattern with all participants included			
	Room/location was accessible			
	The plan for the group session was discussed			
	Facilitators endeavoured to engage all the participants.			
	Facilitators used inclusive/accessible communication styles			
Response of participants	All participants contributed during the group, e.g. speaking to each other, speaking aloud in the group			
	Participants were distracted by competing interests, phone calls			
	There was a clear outcome to the session (e.g. barrier identified, plan agreed)			
	Participants appeared positive and engaged about the group			

For peer review only

BMJ Open

Is it feasible to implement a community-based participatory group programme to address issues of access to healthcare for people with disabilities in Luuka district Uganda? A study protocol for a mixed-methods pilot study

Journal:	*BMJ Open*
Manuscript ID	bmjopen-2023-074217.R1
Article Type:	Protocol
Date Submitted by the Author:	18-Jul-2023
Complete List of Authors:	Kuper, Hannah; London School of Hygiene & Tropical Medicine, Department of Population Health<br>Ssemata, Andrew Sentoogo; MRC/UVRI and LSHTM Uganda Research Unit<br>Smythe, Tracey; London School of Hygiene and Tropical Medicine, Clinical Research<br>Drazdzewska, Joanna; Women and Children First<br>Waiswa, Peter; Makerere University College of Health Sciences, School of Public health; Karolinska Institutet,<br>Kagurusi, Patrick; Amref Health Africa in Uganda<br>Rosato, Mikey; Women and Children First (UK)<br>Mbazzi, Femke Bannink; London School of Hygiene & Tropical Medicine, Department of Population Health; UVRI and LSHTM Uganda Research Unit
**Primary Subject Heading**:	Global health
Secondary Subject Heading:	Global health, Health services research
Keywords:	MENTAL HEALTH, Health Services Accessibility, Developmental neurology & neurodisability < PAEDIATRICS

SCHOLARONE™
Manuscripts

Is it feasible to implement a community-based participatory group programme to address issues of access to healthcare for people with disabilities in Luuka district Uganda? A study protocol for a mixed-methods pilot study

Hannah Kuper[1], Andrew Sentoogo Ssemata[2,3], Tracey Smythe[1,4], Joanna Drazdzewska[5], Peter Waiswa[6], Patrick Kagurusi[7], Mikey Rosato[5], Femke Bannink Mbazzi[1,2]

[1]International Centre for Evidence in Disability, Department of Population Health, London School of Hygiene & Tropical Medicine, London, UK

[2] MRC/UVRI and LSHTM Uganda Research Unit, Entebbe, Uganda.

[3] Department of Global Health and Development, London School of Hygiene & Tropical Medicine, London, UK

[4] Division of Physiotherapy, Department of Health and Rehabilitation Sciences, Stellenbosch University, Cape Town, South Africa

[5] Women and Children First (UK), London, UK

[6] School of Public Health, Makerere University, Kampala, Uganda

[7] Amref Health Africa in Uganda, Kampala, Uganda

Author for correspondence: Hannah Kuper, International Centre for Evidence in Disability, Department of Population Health, London School of Hygiene & Tropical Medicine, Keppel Street, London WC1E 7HT, UK. Email: Hannah.kuper@lshtm.ac.uk

KEYWORDS
Disability; community-based participatory learning and action; pilot; evaluation; Uganda; Healthcare access

For peer review only

ABSTRACT

Introduction

On average, people with disabilities face many difficulties in accessing healthcare and experience worse health outcomes. Yet, evidence on how to overcome these barriers is lacking. Participatory approaches are gaining prominence as they can generate low-cost, appropriate and scalable solutions. This study protocol is for the pilot-testing of the co-created Participatory Learning and Action for Disability (PLA-D) groups to assess feasibility.

Methods and analysis

We will pilot-test PLA-D in five groups in Luuka district, Uganda during 2023. Each group will include approximately 20 members (people with disabilities, family members, carers) who will meet every 2-3 weeks over a 9-11 month period. The groups, guided by a trained facilitator, will identify issues about health and healthcare access and plan and implement locally-generated solutions (e.g. raising awareness of rights, advocacy and lobbying, establishing health savings and financing schemes). We will collect diverse sources of data to assess feasibility: 1) in-depth interviews and focus group discussions with group participants, non-participants, and group facilitators, 2) monitoring of group activities; 3) direct observation of groups, 4) quantitative survey of group participants at baseline and endline. Data analyses will be undertaken to assess feasibility in terms of: acceptability, demand, implementation and practicality. We will develop and refine evaluation tools in preparation for a future trial.

Ethics and dissemination

Ethical approval for the study has been received by the London School of Hygiene & Tropical Medicine, and the Uganda Virus Research Institute ethics committees. Informed consent will be obtained from all study participants, making adaptations for people with disabilities as necessary. We will reach different groups for our dissemination activities, including 1) People with disabilities (e.g. community meetings); 2) Policy and programme stakeholders in Uganda and international (e.g. individual meetings, evidence briefs); 3) Academics (journal articles, conference/seminar presentations).

Strengths and limitations of this study

- Key strengths of the pilot-study is that the feasibility of the programme will be assessed using mixed-method approaches, with participation from people with disabilities.
- A limitation is that the pilot-study is only in one setting and will therefore allow assessment of some domains of the Bowen feasibility framework (acceptability, demand, implementation, practicality) but not others (adaptation, integration, expansion, limited efficacy).
- The limited number of participants included in the pilot study may make it difficult to draw inferences for feasibility for different sub-groups, such as people with different impairment types.

For peer review only - http://bmjopen.bmj.com/site/about/guidelines.xhtml

INTRODUCTION

There are at least 1.3 billion people with disabilities globally, more than 80% of whom live in Low and Middle-Income Countries (LMICs).(1, 2) People with disabilities include those who have long-term physical, mental, intellectual or sensory impairments which, in interaction with various barriers, may hinder their full and effective participation in society on an equal basis with others.(2, 3) People with disabilities face a wide range of discrimination and negative attitudes.(2) They are consequently are more likely to be poor, and excluded from education, employment, and societal participation.(2) Another key challenge facing many people with disabilities is exclusion from healthcare and difficulties achieving good health,(1, 2, 4-7) which is the focus of the current study.

On average, people with disabilities have worse health than others in the population.(1, 2, 4-6) By definition, people with disabilities have an underlying health condition and impairment (e.g. diabetes, physical impairment), which are linked to other health risks (e.g. stroke, pressure sores). They are also on average older, poorer and more marginalised, and have a higher prevalence of a range of risk factors (e.g. violence, physical inactivity, diabetes, hypertension).(1, 2, 6) People with disabilities therefore, on average, have greater general healthcare needs than others in the population.(1, 4) They will also require regular healthcare services like anyone else in the population (e.g. vaccinations) and potentially also specialised healthcare services (e.g. physiotherapy). However, they frequently experience widespread barriers to accessing healthcare, including lack of accessible transport and facilities, poor skills of healthcare providers around disability, and high costs.(1, 7) Consequently, people with disabilities are 41-57% more likely to have unmet healthcare needs, according to the WHO's World Report on Disability.(2) Quality and affordability of healthcare services is also often worse for people with disabilities; They are twice as likely to find healthcare providers skills and facilities inadequate, three times more likely to be denied healthcare, and four times more likely to be treated badly in the healthcare system.(2) The WHO report also found that half of people with disabilities cannot afford healthcare, and they are 50% more likely to suffer catastrophic health expenditure.(2) As a result of all these factors, people with disabilities on average have worse health and higher mortality rates than others in the population, including in the African context.(2, 8-11) These patterns can also be observed in Uganda, the context for the current study, where the 2014 census estimated that 12.5% of the population have disabilities.(12) Studies have shown that people with disabilities in Uganda are more likely to experience poverty and exclusion, poor health, and difficulties accessing services and information.(12-17)

Improving access to healthcare for people with disabilities is therefore an important priority for a number of reasons. We will fail to achieve health targets and development goals without an explicit focus on disability, including Sustainable Development Goal (SDG) 3 on "Good health for all" and Universal Health Coverage (UHC).(18) Other SDGs, including those related to education and employment, will also not be met if people with disabilities continue to have poor health. Exclusion from healthcare is a violation of rights of people with disabilities, as set out in the United Nations Convention on the Rights of Persons with Disabilities (UNCRPD) and in the laws of most countries, including Uganda.(18, 19) Good healthcare also matters to people with disabilities and their families as it affects their ability to survive and enjoy good health. Improving healthcare access for people with disabilities also has the potential to be cost-saving,(1) as it may prevent unnecessary cost for the health system and improve health services for all through universal design.

For peer review only - http://bmjopen.bmj.com/site/about/guidelines.xhtml

Rationale for development of a participatory intervention to improve access to healthcare for people with disabilities

Two systematic reviews on access to general and specialist healthcare for people with disabilities in LMICs found few examples of interventions to address the widespread gaps observed.(20, 21) An Evidence and Gap Map on disability-inclusive development also failed to identify good examples of disability-inclusive health interventions.(22) The WHO has made specific recommendations for the promotion of disability-inclusive health,(1, 2) including 1) removing physical barriers to health facilities, information and equipment, 2) making healthcare affordable, 3) training all healthcare workers in disability issues including rights, 4) investing in specific services such as rehabilitation. These "top-down" interventions are important, but unlikely to be sufficient since barriers will vary locally.(7) Participation of people with disabilities is therefore critically important for developing new locally- appropriate solutions – particularly in light of the "Nothing about us, without us" ethos of the disability movement. Participatory approaches are also recommended by the new WHO report on health equity for persons with disabilities.(1)

Participatory approaches are gaining attention from the global health community as they can empower communities to work together to identify local issues and together generate low-cost, appropriate and scalable solutions. A leading example is the Participatory Learning and Action (PLA) approach, which was designed to reduce maternal and neonatal mortality.(23) Through PLA, women's groups are convened and are facilitated to identify underlying barriers to care and care-seeking and then develop, implement and evaluate local solutions to address these issues (Figure 1). Examples of solutions implemented include the creation of emergency crisis funds to pay for medical care for those in poverty, arranging a bicycle ambulance to bring people to hospital, and lobbying local health authorities for more staff. PLA supports care-seeking and home-based care practices through these pathways, and thereby improves health and wellbeing and reduces mortality in women and new-borns. A meta-analysis of 7 RCTs from 4 countries found that PLA was associated with a 22% reduction in maternal mortality and 20% reduction in neonatal mortality.(23) Sub-group analysis of 4 RCTs where at least 30% of pregnant women participated in groups showed even greater reductions in both maternal mortality (49%) and neonatal mortality (33%).(23) Evidence also shows that the PLA methodology is sustainable,(24) scalable,(25) and equitable.(26) It is recommended by WHO as a strategy for improving maternal and new-born health and reducing mortality, particularly in rural settings where mortality rates are high and access to services is low.(27) PLA has been successfully implemented in 15 countries, including in several settings Uganda.

To date, PLA has not been used explicitly for people with disabilities. However, evidence from Nepal showed that there was no difference in group attendance between women with and without disabilities.(28) A non-PLA participatory approach has been used in the Philippines to try to improve access to sexual and reproductive healthcare for people with disabilities through establishment of community groups - W-DARE project (Women with Disability taking Action on REproductive and sexual health.(29) Five groups of women with disabilities (established by impairment type) and one group of parents of children with disabilities met fortnightly for ten meetings to identify and discuss key sexual and reproductive healthcare access issues. A qualitative evaluation highlighted positive benefits of the programme, including increased knowledge and confidence of the women to seek services.(30) Other participatory approaches to promote disability inclusive-health are lacking.

We hypothesise that PLA for people with disabilities (PLA-D) would improve healthcare access, health and wellbeing and reduce mortality of people with disabilities in Uganda. The PLA-D approach is expected to work from the bottom-up as people with disabilities identify and tackle locally-relevant barriers. The potential drivers of action are that group meetings

For peer review only - http://bmjopen.bmj.com/site/about/guidelines.xhtml

allow people with disabilities to meet and identify local issues, and develop and implement solutions that are self-resourced. Existing programmes show that solutions implement address a range of issues, such as lack of awareness (e.g. education on health issues and rights), lack of decision-making authority on health (e.g. lobby families to fund healthcare of person with disabilities) or poverty (e.g. livelihood programmes). The intervention will be supported by health system strengthening activities to improve healthcare access and quality by improving the knowledge, attitudes and skills of healthcare workers and helping identify issues around accessibility of facilities. Furthermore, groups also frequently undertake advocacy activities, for instance, to encourage other members in the community to become involved and take action (e.g. support transport to healthcare facilities). Groups can also lobby decision-makers to encourage policy and programme change and raise awareness on disability. Through these actions, people with disabilities are anticipated to experience better access to healthcare, improved health and wellbeing, and ultimately reduced mortality. The current protocol focusses on the group meetings (PLA-D). We have been guided by the MRC framework in the development of the PLA-D approach, and proposed feasibility assessment, and in the future plan to undertake an evaluation of the intervention.(31)

Adaptation of PLA for people with disabilities

In 2022, we undertook qualitative research with people with disabilities with a range of impairment types (n=27), healthcare workers (n=15) and other stakeholders, including carers (n=11), to describe barriers and facilitators to accessing healthcare by people with disabilities in Uganda and assess the demand for PLA-D. We also reviewed the literature to identify good practice for improving access to healthcare for people with disabilities, including existing systematic reviews,(20-22) key source documents,(2, 6, 32, 33) and a good practice compendium.(34) This activity was supplemented through interviews with key stakeholders, including PLA experts, Organizations of Persons with Disabilities (OPDs), inclusive health experts.

We held a 4-day design workshop in October 2022 to co-create the adapted PLA approach to be accessible, relevant and appropriate for people with disabilities in Uganda (PLA-D), facilitated by Women and Children First. Participants included the research team (n=7), PLA experts (including Amref and Makerere University n=5), health system stakeholders (e.g. District Health Officers n=2), district disability focal person from the community development office (n=1), people with disabilities and representatives from National OPDs (n=8). The group worked together to agree how to adapt the logistics of the intervention delivery (e.g. identifying who the facilitators should be, where groups should be convened, addressing accessibility) and identified appropriate local implementation and OPD partners (Figure 2). The PLA content was then adapted with reference to recommendations from the PLA evidence base and literature, including the facilitator manual, materials (e.g. pictures, cards) and session plans. We updated the Theory of Change for PLA to reflect how the intervention is anticipated to have the expected impact (Figure 3).

This protocol is for a study to pilot-test the co-created Participatory Learning and Action for Disability (PLA-D) groups in order to assess their feasibility, and to inform the design of a future cluster randomised controlled trial.

For peer review only - http://bmjopen.bmj.com/site/about/guidelines.xhtml

METHODS AND ANALYSIS

Overview

A mixed-methods pilot-study will be undertaken in 2023 to assess the feasibility of the PLA-D groups and inform the future development of a cluster randomised controlled trial. The research will be undertaken in Luuka district, Eastern Uganda, as a partnership between researchers from the London School of Hygiene & Tropical Medicine, MRC/ UVRI & LSHTM Uganda Research Unit ("Uganda Research Unit"), Makerere University, and the NGOs Women and Children First, UK and Amref Health Africa in Uganda. Luuka district had a population of 238,000 in the 2014 census. It is the setting of ongoing research by Makerere, and so Makerere has strong existing networks in the district which will facilitate project implementation. The population is >95% rural and almost all households are engaged in agricultural activities.

Conceptualisation of disability

The UN Convention on the Rights of Persons with Disabilities conceptualizes people with disabilities as … *"those who have long-term physical, mental, intellectual or sensory impairments which in interaction with various barriers may hinder their full and effective participation in society on an equal basis with others."*(3) This definition is in line with the interpretation of disability in the Uganda Persons with Disabilities Act, 2020 as *"a substantial functional limitation of a person's daily life activities caused by physical, mental or sensory impairment and environment barriers, resulting in limited participation in society on equal basis with others and includes an impairment specified in Schedule 3 to this Act."* For the purpose of the pilot study, people will self-identify as whether they have a disability or not.

Pilot of the content and implementation of PLA-D groups

We will establish five pilot PLA-D groups in one sub-country in Luuka district, in May-June 2023. The sample size was selected based on logistical feasibility (time, costs) and our previous experience of pilot-testing a group-based intervention suggested that this number was sufficient to assess feasibility of the intervention.(35)

In collaboration with the District Health Officers, we will select the communities to represent a variety of contexts (e.g. very rural or semi-urban, remote or less remote areas). We will work with local community members (e.g. Community health worker [CHWs] or Community Health Extension Workers [CHEWs]) to identify people with disabilities in the community. People with disabilities will then be given information about the purpose and structure of the PLA-D groups and invited to attend. Membership will also be open to family members/ caregivers of people with disabilities, although emphasis will be given on the participation of people with disabilities. We anticipate that each group will include approximately 20 people, potentially at least half of whom will be people with disabilities.

Potential group members, with and without disabilities, will be identified and recruited through the community engagement meetings held at the five different parishes (one per group). Information about the study and groups will be provided and those interested in participation will be asked to sign up/ register their names and contact and will be contacted individually by the research team. Additional people with disabilities will be invited to attend by the focal person with disabilities at the district level, and by people with disabilities who attended the community engagement meetings.

For peer review only - http://bmjopen.bmj.com/site/about/guidelines.xhtml

Groups will meet every 2-3 weeks over a 9 month period (ending December 2023/January 2024), and will go through the PLA cycle (Figure 1). The groups will together choose the location of the meetings, ideally selected to promote accessibility, as well as the timing. The PLA cycle includes sessions on identifying issues in accessing healthcare, developing and implementing solutions (e.g. savings schemes, growing nutritious food), and self-evaluating their impact. Other community members will be reached through regular community members.

The groups will be facilitated by a facilitator (person with disabilities from the local community and/or a CHW). The facilitators will be supported by trained supervisors and managers, which will be coordinated by the NGO Amref. The facilitators, managers and supervisor will be involved in a week-long hands-on PLA-D training, led by Women and Children First, Amref and the Uganda Research Unit, based on the developed manual. The training will cover aspects including understanding of disability, the PLA-D cycle, how group meetings will be conducted and management and facilitation of the groups in the community (e.g. safeguarding, managing expectations, progress recording and reporting).

Feasibility assessment

We will use the Bowen framework to consider the feasibility of the PLA-D groups.(36) Our main focus will be on the first four components of the framework: acceptability, demand, implementation, and practicality. We will not consider the components of adaptation, integration and expansion for this pilot study, as they relate to issues of scaling existing programmes. Limited-efficacy is also not considered, but effectiveness will be considered in a future randomised controlled trial. We will develop and refine evaluation tools of the groups to inform this future study.

There are four main sources of data collection.

1) Programme monitoring

Attendance of sessions and frequency of sessions will be monitored through a simple register which will be collected by the facilitators.

2) Pre-post quantitative survey (Supplementary Materials 1 and 2)

A survey will be completed by adult PLA-D group participants with disabilities at the programme first and last session (either directly, or via a proxy). These will be developed in English and translated into Luganda and Lusoga (administered in the language of choice of the respondent) and will include:

- Socio-demographic characteristics of the participant
- Disability assessment (Washington Group Enhanced Short Set)
- Quality of Life (WHO-BREF)
- Health condition and nutrition
- Health care utilization
- Participation
- Attitudes
- Main goals for the intervention*
- Review of goals achieved**
- Satisfaction with group programme**

*Only asked at baseline; **only asked at end-line

For peer review only

Questionnaires are based upon existing tools (WHOQOL BREF, WOPS questionnaire from cohort in Uganda, WHO Model Disability Survey, and SINTEF survey), which have all been widely used. Any potentially controversial questions have been removed (i.e. SINTEF questions on violence). A separate questionnaire has been developed for children aged 5-17 years, to be completed by the parent/carer as a proxy. Questionnaire data will not be collected for children <5 years.

All questionnaires will be interviewer- administered in order to maintain consistency and uniformity in interpretation of the questions. Interviews will be undertaken by researchers from the Uganda Research Unit. All questionnaires will be translated in Lusoga and Luganda, the most common local languages in Luuka district. Proxy respondents will be used for adults with difficulties understanding or communicating, and with children aged <18 years. Children aged 8-17 will be asked to give assent to the questionnaire being completed on their behalf.

3) Semi-structured interviews and Focus Group Discussions (Supplementary materials 3-6)

Qualitative data will be collected from a range of groups, led by researchers from Uganda Research Unit.

Semi-structured in-depth interviews (IDIs) will be conducted with the group facilitators after each session. The researchers will conduct phone interviews with the group facilitator after each session to review the progress of the session, identify areas that went well, challenges that arose and areas for improvement. This information will be used to allow fast-track learning so that future sessions by that or other groups can be adapted in line with recommendations (e.g. tips for promoting inclusion, location of meetings, etc).

Additionally, semi-structured IDIs and focus group discussions (FGDs) will be undertaken with group participants and facilitators after the final session. The IDI will be undertaken in the language of choice of the respondent (English or Luganda or Lusoga) with a focus on: 1) satisfaction with the groups, 2) perceived challenges, facilitators and barriers to successful group implementation, 3) group capacity, 4) areas for improvement, and 5) perceived positive and negative impacts. Interviews will conducted face-to-face (wherever possible) or remotely. Proxy respondents will be used for adults with difficulties understanding or communicating, and with children aged <18 years.

The IDIs and FGDs will include:

- FGDs with PLA-D participants of the five groups excluding those who participated in the IDIs (6-8 participants per group, 30-40 in total).

- IDIs with 10-15 PLA-D group participants (2-3 participants per group). Participants will be selected to reflect a range of type and severity of disability, gender and socio-economic profile. They will include people with disabilities and caregivers of persons with disabilities and PLA-D members without disabilities. These interviews will support triangulation of data from the FGDs and further inquiry of areas for further exploration raised during the FDGs.

- IDI with 10-15 people eligible to be PLA-D group participants, but who attended 3 or fewer sessions (including no sessions). Participants will be selected to reflect a range of type and severity of disability, gender and socio-economic profile. They will include people with disabilities and caregivers of persons with disabilities.

- IDI with each of the group facilitators (total = 5).

For peer review only - http://bmjopen.bmj.com/site/about/guidelines.xhtml

Topic guides are available in the supplement.

We anticipate that each interview will take approximately 45-60 minutes, and the Focus Group Discussion 60-90 minutes. Carer/proxy interviews will be conducted for people with severe difficulties understanding or communicating even with available adaptations (e.g. people with hearing loss, illiterate and with no knowledge of sign language; people with severe intellectual/cognitive impairments). Inclusion of people with disabilities will be supported through sign language interpretation as needed, accessible interview sites and transport, and researchers skilled at communicating with people with cognitive impairments. Carer/proxy interviews will also be used for children with disabilities below the age of 18 years.

4) Direct observation of groups (Supplementary materials 7)

Direct observation of sessions will be undertaken by the local researchers using a checklist guide. The checklist will contain information about the participatory approaches used by facilitators, the level of interest and engagement of participants and notes of any aspects of the group meeting which went well or did not go well.

Data management and storage

The interviewers will audio record the interviews/focus group discussion. Interviews will be transcribed and translated to English (for those conducted in Luganda or Lusoga). Anonymised transcripts with pseudonyms will be used during the analysis. Quantitative survey data will be anonymised using participant codes and entered into a REDCap database for analysis. All data will be kept as per General Data protection regulation requirements.

Data will be kept strictly confidential (e.g. anonymisation of data to remove personal identifiers, upload/download of data through secure networks and password protecting files). Names of participants will not be given on any documentation and no individuals will be identified in any publication.

Analysis plan

We will use the data to evaluate the feasibility of PLA-D implementation focusing on four of the Bowen components of feasibility (Table 1).(36) Qualitative analysis will be led by two researchers from the Uganda Research Unit (AS, FBM), who are both Ugandan nationals and both have post-graduate degrees in qualitative research. A thematic approach will be used to analyse qualitative data. The data will be coded using NVivo 12, specialist software for qualitative data analysis, and the data will be analysed to develop a fuller framework of themes and subthemes. Particular attention will be given to the feasibility of implementation for people across the full range of impairment types, and whether it addresses the needs of both adults and children.

We will perform descriptive analysis of quantitative data using STATA by a trained epidemiologist (HK). In particular, we will provide data on the levels of knowledge, attitudes and behaviours, self-rated health, and health-seeking behaviour for the study population at baseline and endline. We will analyze data as means with standard deviations, medians with interquartile ranges, or frequencies or perecentages, depending on the indicators. Differences in outcomes between baseline and endline will be described (e.g. comparing change in means or proportions), using appropriate tests (e.g. t-tests, Chi square tests), comparing both all participants and then participants only with both baseline and endline data. Indicators for inclusion in the future randomised controlled trial will be considered (e.g. based upon feasibility of collection, range in answers provided). Indication will also be given of potential

For peer review only - http://bmjopen.bmj.com/site/about/guidelines.xhtml

effect sizes for the planned RCT, based on pre-post differences, to allow calculation of required sample size.

Patient and public involvement

The development and pilot-testing of PLA-D will be in partnership with people with disabilities. During development phase this included: sharing plans with OPDs and people with disabilities; interviewing people with disabilities on their perceived healthcare needs and suggestions for group activities; including people with disabilities in the advisory group and in the co-design workshop. People with disabilities will also have strong involvement in the implementation and pilot-testing of PLA-D, as: group members; group facilitators (wherever possible); research study participants; researchers/data collectors (wherever possible); advisory group members.

ETHICS AND DISSEMINATION

Ethics and consent

Ethical approval for the study has been received from the London School of Hygiene & Tropical Medicine, the Uganda Virus Research Institute ethics committee, and the Ugandan National Council for Science and Technology.

Consent to participate in the study will be sought from each participant only after a full explanation has been given, an information leaflet offered and time allowed for consideration. Signed participant consent will be obtained (including thumb print if necessary). The right of the participant to refuse to participate without giving reasons will be respected. All participants are free to withdraw at any time from the study without giving reasons and without prejudicing further treatment or PLA-D group participation. For any participant with communication or intellectual impairments, we will seek consent from their caregiver. Carer/parent interviews will also be used for children with disabilities below the age of 18. We will seek verbal assent from children aged 8-17 years for a questionnaire to be completed on their behalf.

There is a chance that participants may become distressed or upset during the interviews when discussing stigma and discrimination. To minimise the risk of distress during interviews/questionnaires/focus group discussions: 1) the right of the participant to refuse to answer questions or withdraw from the study at any point will be emphasised 2) Interviewers will be trained to listen non-judgementally, provide short breaks, be aware of signs of distress, fatigue or anxiety and to address concerns appropriately and signpost to available support mechanisms.

Referrals and reimbursement

Group facilitators will make PLA-D group participants aware of local health services available and will encourage them to attend for services if they have an expressed healthcare need. If disclosures are made in the group of safety concerns (e.g. experience of violence) then the group facilitator will liaise with the local researcher for course of action.

We will provide reimbursement of 25,000 Ugandan Shillings (approximately £5.60) for participants in interviews, questionnaires, or focus group discussions to compensate them for their time, and in compliance with Ugandan guidance. Refreshments will be provided at group meetings.

Dissemination plan

For peer review only - http://bmjopen.bmj.com/site/about/guidelines.xhtml

Research is most worthwhile if it can be used to improve policy and practice. We will develop tailored strategies for engagement and communication with our key audience to facilitate influence of policy and practice, using our strong national and international networks, including:

- Community members and people with disabilities: hosting dissemination meetings in the community and with OPDs.

- Policy and programme stakeholders in Uganda (e.g. health systems actors, OPDs and NGOs): Individual meetings with key organizations, production of policy and evidence briefs, relevant to Uganda.

- International policy and programme stakeholders: Production of policy and evidence briefs, including through the Missing Billion Initiative, meetings with key stakeholders, contribution to WHO World Report on Disability and Health.

- Academics: Peer-reviewed journal articles, presentations at conferences/seminars/webinars, and use of material in teaching, including LSHTM's online teaching and online research seminars (e.g. LSHTM, Uganda Research Unit).

Potential limitations

A key limitation is that the pilot-study will only be conducted in one setting. Consequently, it will only allow assessment of some domains of the Bowen feasibility framework (acceptability, demand, implementation, practicality) but not others (adaptation, integration, expansion, limited efficacy). It will therefore not be possible to make inferences on feasibility in other settings, or potentials for scale and impact. Moreover, we will include a limited number of participants in the pilot study, which will make it difficult to draw inferences for feasibility for different sub-groups, such as people with different impairment types. We plan to undertake a full RCT following the feasibility study, which would allow assessment of impact and exploration of feasibility for different sub-groups. There is a concern that participants will respond positively about the feasibility of the intervention because of social desirability bias. We will therefore use objectives markers of feasibility (e.g. group attendance), as well as subjective measures (e.g. reported satisfaction), and will train the interviewers carefully about how to avoid this bias (e.g. asking neutral questions).

For peer review only - http://bmjopen.bmj.com/site/about/guidelines.xhtml

REFERENCES

1. WHO. Global report on health equity for persons with disabilities. Geneva: World Health Organzation; 2022.
2. WHO. World Report on Disability. Geneva: World Health Organisation; 2011.
3. UN. Convention on the rights of persons with disabilities. New York: United Nations; 2006.
4. Kuper H, Heydt P. The Missing Billion. London: LSHTM; 2019.
5. Missing Billion Initiative and Clinton Health Action Inititiative. Reimagining health systems that expect, accept and connect 1 billion people with disabilities.: Missing Billion Initiative and Clinton Health Access Inititiative; 2022.
6. UNDESA. UN Flagship Report on Disability and Development. UNDESA; 2018.
7. Hashemi G, Wickenden M, Bright T, *et al*. Barriers to accessing primary healthcare services for people with disabilities in low and middle-income countries, a Meta-synthesis of qualitative studies. Disabil Rehabil. 2020:1-14.
8. Prynn JE, Dube A, Mkandawire J, *et al*. Self-reported disability in relation to mortality in rural Malawi: a longitudinal study of over 16 000 adults. BMJ Open. 2020;10(8):e034802.
9. Kuper H, Mathenge W, Macleod D, *et al*. Mortality during 6 years of follow-up in relation to visual impairment and eye disease: results from a population-based cohort study of people aged 50 years and above in Nakuru, Kenya. BMJ Open. 2019;9(6):e029700.
10. Gómez-Olivé FX, Thorogood M, Bocquier P, *et al*. Social conditions and disability related to the mortality of older people in rural South Africa. Int J Epidemiol. 2014;43(5):1531-41.
11. Abuga JA, Kariuki SM, Kinyanjui SM, *et al*. Premature mortality in children aged 6-9 years with neurological impairments in rural Kenya: a cohort study. Lancet Glob Health. 2019;7(12):e1728-e35.
12. Uganda Bureau of Statistics. The National Population and Housing Census 2014 – National Analytical Report on persons with disabilities. Kampala, Uganda: Uganda Bureau of Statistics; 2019.
13. Abimanyi-Ochom J, Mannan H, Groce NE, *et al*. HIV/AIDS knowledge, attitudes and behaviour of persons with and without disabilities from the Uganda Demographic and Health Survey 2011: Differential access to HIV/AIDS information and services. PLoS One. 2017;12(4):e0174877.
14. Wandera SO, Golaz V, Kwagala B, *et al*. Factors associated with self-reported ill health among older Ugandans: a cross sectional study. Arch Gerontol Geriatr. 2015;61(2):231-9.
15. Nakamanya S, Siu GE, Lassman R, *et al*. Maternal experiences of caring for an infant with neurological impairment after neonatal encephalopathy in Uganda: a qualitative study. Disabil Rehabil. 2015;37(16):1470-6.
16. Scholten F, Mugisha J, Seeley J, *et al*. Health and functional status among older people with HIV/AIDS in Uganda. BMC Public Health. 2011;11:886.
17. Nyirenda M, Newell M-L, Mugisha J, *et al*. Health, wellbeing, and disability among older people infected or affected by HIV in Uganda and South Africa. Global health action. 2013;6:19201.
18. Kuper H, Hanefeld J. Debate: can we achieve universal health coverage without a focus on disability? BMC Health Serv Res. 2018;18(1):738.
19. Government of Uganda. Uganda Persons with Disabilities Act, 2020, (2020).
20. Bright T, Kuper H. A Systematic Review of Access to General Healthcare Services for People with Disabilities in Low and Middle Income Countries. Int J Environ Res Public Health. 2018;15(9).

For peer review only - http://bmjopen.bmj.com/site/about/guidelines.xhtml

21. Bright T, Wallace S, Kuper H. A Systematic Review of Access to Rehabilitation for People with Disabilities in Low- and Middle-Income Countries. Int J Environ Res Public Health. 2018;15(10).
22. Saran A, White H, Kuper H. Evidence and gap map of studies assessing the effectiveness of interventions for people with disabilities in low-and middle-income countries. Campbell Systematic Reviews. 2020;16(1).
23. Prost A, Colbourn T, Seward N, *et al*. Women's groups practising participatory learning and action to improve maternal and newborn health in low-resource settings: a systematic review and meta-analysis. Lancet. 2013;381(9879):1736-46.
24. Sondaal AEC, Tumbahangphe KM, Neupane R, *et al*. Sustainability of community-based women's groups: reflections from a participatory intervention for newborn and maternal health in Nepal. Community Dev J. 2019;54(4):731-49.
25. Nair N, Tripathy PK, Gope R, *et al*. Effectiveness of participatory women's groups scaled up by the public health system to improve birth outcomes in Jharkhand, eastern India: a pragmatic cluster non-randomised controlled trial. BMJ Glob Health. 2021;6(11).
26. Morrison J, Osrin D, Alcock G, *et al*. Exploring the equity impact of a maternal and newborn health intervention: a qualitative study of participatory women's groups in rural South Asia and Africa. Int J Equity Health. 2019;18(1):55.
27. WHO. Every Newborn Action Plan. Geneva: WHO; 2014.
28. Morrison J, Colbourn T, Budhathoki B, *et al*. Disabled women's attendance at community women's groups in rural Nepal. Health Promot Int. 2017;32(3):464-74.
29. Vaughan C, Zayas J, Devine A, *et al*. W-DARE: a three-year program of participatory action research to improve the sexual and reproductive health of women with disabilities in the Philippines. BMC Public Health. 2015;15:984.
30. Devine A, Ignacio R, Prenter K, *et al*. "Freedom to go where I want": improving access to sexual and reproductive health for women with disabilities in the Philippines. Reprod Health Matters. 2017;25(50):55-65.
31. MRC. Developing and evaluating complex interventions. London: MRC; 2006.
32. WHO. Policy brief on disability-inclusive health systems. Copenhagan, Denmark: WHO EURO; 2021.
33. UNDESA. Toolkit on disability for Africa; inclusive health services for persons with disabilities. 2016.
34. Missing Billion. Good Practice Compendium 2022 [Available from: https://www.themissingbillion.org/good-practices.
35. Duttine A, Smythe T, Calheiros de Sá MR, *et al*. Assessment of the feasibility of Juntos: A support programme for families of children affected by Congenital Zika Syndrome. Wellcome Open Res. 2022;7:77.
36. Bowen DJ, Kreuter M, Spring B, *et al*. How we design feasibility studies. Am J Prev Med. 2009;36(5):452-7.

For peer review only

AUTHORS CONTRIBUTIONS

The study concept and design was conceived by HK, TS, FBM and PW. JD, MR, PK and PW informed the development of PLA-D. FBM and ASS will lead on the data collection. Analysis will be performed by HK, FBM and ASS. HK prepared the first draft of the manuscript. All authors provided edits and critiqued the manuscript for intellectual content.

FUNDING STATEMENT

This work is supported by NIHR grant number NIHR301621. Conceptualization of the programme was supported by the PENDA grant from the Foreign, Commonwealth and Development Office.

COMPETING INTERESTS

None declared

FIGURE LEGENDS

Figure 1: The PLA-D group cycle has 11 meetings across 4 phases
Figure 2: Decisions reached on design of PLA-D
Figure 3: PLA-D Theory of Change

For peer review only

Table 1: Feasibility assessment of PLA-D: domains explored and data to be used (36)

Bowen domain	To what extent…..	Example outcomes	Data used
Acceptability	… is PLA-D judged as suitable, satisfying or attractive to program recipients (people with disabilities and other potential PLA-D members)	Reported satisfaction; perceived appropriateness; intent to continue use	Direct observation; Qualitative data; Questionnaire
Demand	… is PLA-D likely to be used?	PLA-D group attendance; Perceived demand; expressed interest	Monitoring data; Qualitative data
Implementation	… can PLA-D be successfully delivered to intended participants?	Delivery of meetings; success or failure of PLA-D implementation; facilitators and barriers to implementation	Qualitative data; Direct observation
Practicality	… can PLA-D be carried out with intended participants using existing means, resources and circumstances	Solutions implemented; Positive/negative impacts on participants; Cost analysis	Monitoring data; Qualitative data

For peer review only

For peer review only - http://bmjopen.bmj.com/site/about/guidelines.xhtml

Figure 1: The PLA-D group cycle has 11 meetings across 4 phases

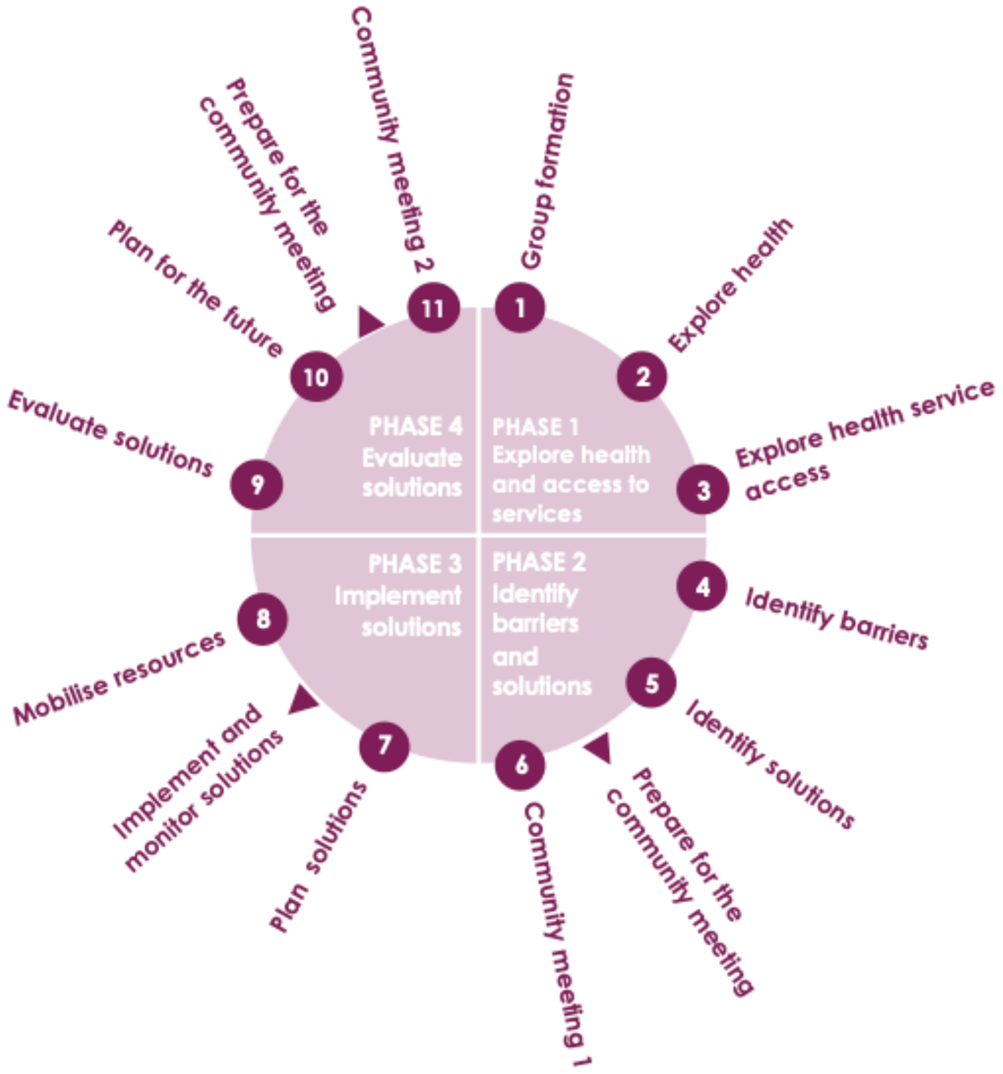

only

Figure 2: Decisions reached on design of PLA-D

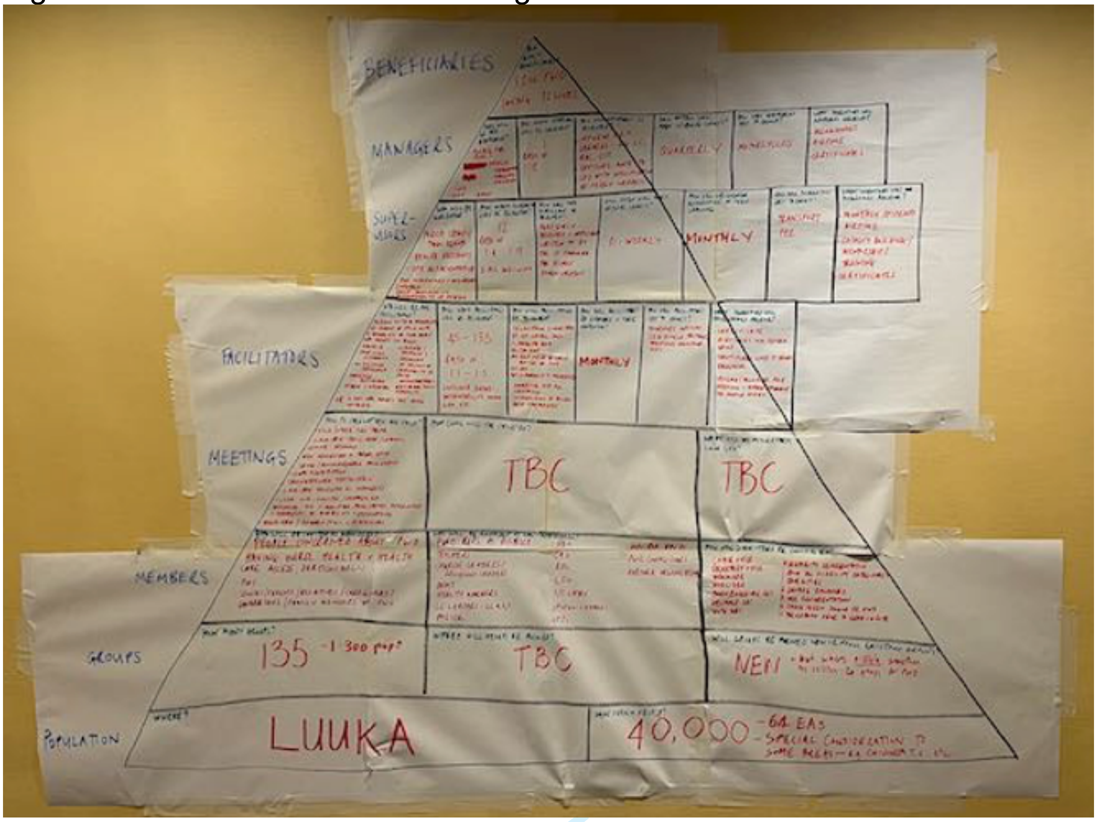

review only

For peer review only

Figure 3: PLA-D Theory of Change

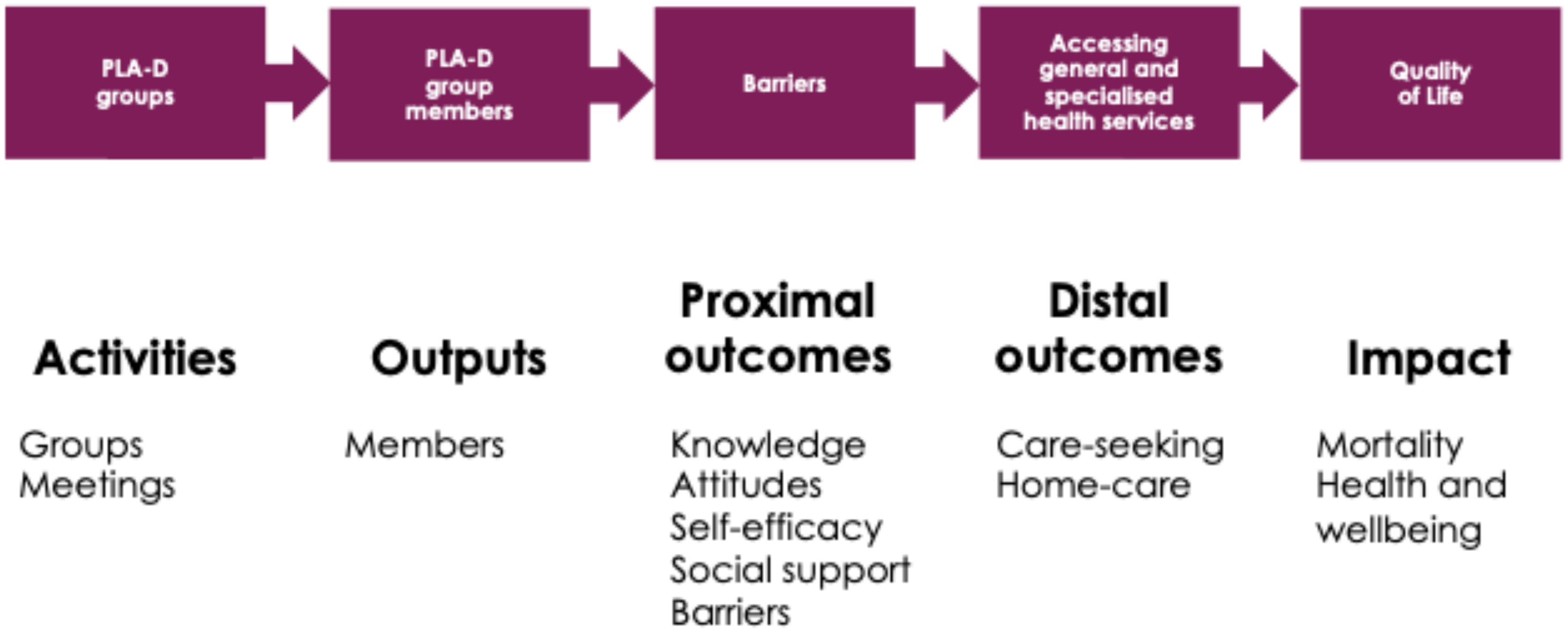

Supplement 1: MRC/UVRI & LSHTM Uganda Research Unit **and LSHTM**

Questionnaire: Adult version

Table of contents

Section 100: Respondent and household characteristics
Section 200: Washington Group Short Set Enhanced
Section 300: Quality of life
Section 400: Health condition and nutrition
Section 500: Health care utilization
Section 600: Participation
Section 700: Attitudes
Section 800: PLA-D specific questions

Nr.		
001	LOCATION ID NUMBER	______________________
002	**ID NUMBER**	______________________
003	**INTERVIEW TYPE**	1=Person with disabilities (direct)<br>2=Proxy for person with disabilities (unable to communicate/understand)<br>2=Proxy for person with disabilities (child<18)<br>______________________
004	**Interviewer 1 initials and code**	
005	**Date of interview**	__ __ 2023<br>(DAY MONTH)
006	**Time start interview**	__ hrs __ min
007	**Time end interview**	__ hrs __ min

For peer review only - http://bmjopen.bmj.com/site/about/guidelines.xhtml

SECTION 100 - RESPONDENT AND HOUSEHOLD CHARACTERISTICS (WOPS)

I am going to start by asking you some questions about yourself and your living situation.

101	SEX	1= Male<br>2= Female
102	How old are you now?	Years……..
103	In what year were you born?<br>**IF KNOWN INCLUDE DATE OF BIRTH; CORRECT INCONSISTENCIES WITH AGE**	Year of birth….
		Date of birth<br>…../……/……….
104	What is current marital status?	1= Single/ Never married<br>2= Married / Cohabitating<br>3= Divorced / Separated<br>4= Widowed
105	What is / was your occupation?<br>Any other?<br>**TICK ALL THAT APPLY**	House work
		Farming
		Fishing
		Labouring (e.g. clearing streets, weeding)
		Tailoring
		Teacher / nurse
		Government administration
		Shop attendant
		other,
105a	Other spouse occupation SPECIFY	____________________
106	What is / was your spouse's occupation? Any other?<br>**TICK ALL THAT APPLY**	House work
		Farming
		Fishing
		Labouring(e.g. clearing streets, weeding etc)
		Tailoring
		Teacher / nurse
		Government administration
		Shop attendant
		Other
106a	Other respondent occupation SPECIFY	__________________
107	Are you still working?	1= Yes, full-time<br>1a= Yes, part-time<br>1b= Yes, self-employed

For peer review only

For peer review only - http://bmjopen.bmj.com/site/about/guidelines.xhtml

		1c= No, retired<br>2= No
108	What is your highest level of education?	1= No formal education<br>2= Less than primary (incomplete) school<br>3= Completed primary school<br>4= complete O level<br>5= Completed A level<br>6= Higher education than secondary school (diploma course or BTVET)<br>7= Adult education only<br>8= College/ University or more<br>88= Don't know
109	What is the total number of people who live in your household?	☐☐<br>Persons

LIVING CONDITIONS

110	What is the predominant material used for roofing?	1= Thatch<br>2= Tile<br>3= Iron sheets<br>4= Mixed iron/tile<br>5= Other
111	What is the state of the roof?	1= Very Good<br>1= Good<br>2= Moderate<br>3= Bad<br>4= Very bad
112	What are the walls of the house made of?	1= Burned brick<br>2= Unburned brick<br>3= Mud
113	What is the house floor made of?	1= Mud<br>2= Cement<br>3= Tiles
114	Who owns the house?	1= Self<br>2= Spouse<br>3= Respondent's children<br>4= Someone else in the household<br>5= Renting (landlord)<br>87= Other

For peer review only

SECTION 200: Washington Group Enhanced Set

**201**	Do you have difficulty seeing even when wearing your glasses/contact lenses? Would you say….	1= No difficulty<br>2= Some difficulty<br>3= A lot of difficulty<br>4= Cannot do at all
**202**	Do you have difficulty hearing, even when using a hearing aid(s)? Would you say…	1= No difficulty<br>2= Some difficulty<br>3= A lot of difficulty<br>4= Cannot do at all
**203**	Do you have difficulty walking or climbing steps? Would you say…	1= No difficulty<br>2= Some difficulty<br>3= A lot of difficulty<br>4= Cannot do at all
**204**	Using your usual languate do you have difficulty communicating, for example, understanding or behing understood? Would you say…	1= No difficulty<br>2= Some difficulty<br>3= A lot of difficulty<br>4= Cannot do at all
**205**	Do you have difficulty remembering or concentrating? Would you say…	1= No difficulty<br>2= Some difficulty<br>3= A lot of difficulty<br>4= Cannot do at all
**206**	Do you have difficulty with self care, such as washing all over or dressing? Would you say…	1= No difficulty<br>2= Some difficulty<br>3= A lot of difficulty<br>4= Cannot do at all
**207**	Do you have difficulty raising a 2 liter bottle of water or soda from the waist to eye level? Would you say…	1= No difficulty<br>2= Some difficulty<br>3= A lot of difficulty<br>4= Cannot do at all
**208**	Do you have difficulty using your hands and fingers, such as picking up small objects, for example, a button or pencil, or opening or closing containers or bottles? Would you say…	1= No difficulty<br>2= Some difficulty<br>3= A lot of difficulty<br>4= Cannot do at all
**209**	How often do you feel worried, nervous or anxious? Would you say…	1= Daily<br>2= Weekly<br>3= Monthly<br>4= A few times a year<br>5= Never
	Thinking about the last time you felt worried, nervous or anxious, how would you describe the level of these feelings. Would you say…	1= A little<br>2= A lot<br>3= Somewhere between a little and a lot
**209**	How often do you feel depressed? Would you say…	1= Daily<br>2= Weekly<br>3= Monthly<br>4= A few times a year

For peer review only - http://bmjopen.bmj.com/site/about/guidelines.xhtml

		5= Never
**210**	Thinking about the last time you felt depressed, how would you describe the level of these feelings. Would you say…	1= A little<br>2= A lot<br>3= Somewhere between a little and a lot
**211**	At what age did you first start experiencing these difficulties	_____ years<br>0 if age<1<br>999 if don't know

For peer review only

For peer review only - http://bmjopen.bmj.com/site/about/guidelines.xhtml

SECTION 300 QUALITY OF LIFE (WHO-QOL BREF)

This assessment asks how you feel about your quality of life, health, or other areas of your life. Please answer all the questions. If you are unsure about which response to give to a question, please choose the one that appears most appropriate. This can often be your first response. Please keep in mind your standards, hopes, pleasures and concerns. We ask that you think about your life in the last two weeks.

301	How would you rate your quality of life?	1= Very poor<br>2= Poor<br>3= Neither poor nor good<br>4= Good<br>5= Very good
302	How satisfied are you with your health?	1= Very dissatisfied<br>2= Disatisfied<br>3= Neither satisfied nor dissatisfied<br>4= Satisfied<br>5= Very satisfied
303	To what extent do you feel that physical pain prevents you from doing what you need to do?	1= Not at all<br>2= A little<br>3= A moderate amount<br>4= Very much<br>5= An extreme amount
304	How much do you need any medical treatment to function in your daily life?	1= Not at all<br>2= A little<br>3= A moderate amount<br>4= Very much<br>5= An extreme amount
305	How much do you enjoy life?	1= Not at all<br>2= A little<br>3= A moderate amount<br>4= Very much<br>5= An extreme amount
306	To what extent do you feel your life to be meaningful?	1= Not at all<br>2= A little<br>3= A moderate amount<br>4= Very much<br>5= An extreme amount

For peer review only - http://bmjopen.bmj.com/site/about/guidelines.xhtml

307	How well are you able to concentrate?	1= Not at all<br>2= A little<br>3= A moderate amount<br>4= Very much<br>5= Extremely
308	How safe do you feel in your daily life?	1= Not at all<br>2= A little<br>3= A moderate amount<br>4= Very much<br>5= Extremely
309	How healthy is your physical environment?	1= Not at all<br>2= A little<br>3= A moderate amount<br>4= Very much<br>5= Extremely
310	Do you have enough energy for your everday activities?	1= Not at all<br>2= A little<br>3= Moderately<br>4= Mostly<br>5= Completely
311	Are you able to accept your bodily appearance?	1= Not at all<br>2= A little<br>3= Moderately<br>4= Mostly<br>5= Completely
312	Have you enough money to meet your needs?	1= Not at all<br>2= A little<br>3= Moderately<br>4= Mostly<br>5= Completely
313	How available to you is the information that you need in your day-to-day life?	1= Not at all<br>2= A little<br>3= Moderately<br>4= Mostly<br>5= Completely
314	To what extent do you have the opportunity for leisure activities?	1= Not at all<br>2= A little<br>3= Moderately<br>4= Mostly<br>5= Completely

For peer review only

315	How well are you able to get around?	1=Very poor<br>2= Poor<br>3= Neither poor nor good<br>4= Good<br>5= Very good
316	How satisfied are you with your sleep?	1= Very dissatisfied<br>2= Disatisfied<br>3= Neither satisfied nor dissatisfied<br>4= Satisfied<br>5= Very satisfied
317	How satisfied are you with your ability to perform your daily activities?	1= Very dissatisfied<br>2= Disatisfied<br>3= Neither satisfied nor dissatisfied<br>4= Satisfied<br>5= Very satisfied
318	How satisfied are you with your capacity for work?	1= Very dissatisfied<br>2= Disatisfied<br>3= Neither satisfied nor dissatisfied<br>4= Satisfied<br>5= Very satisfied
319	How satisfied are you with yourself?	1= Very dissatisfied<br>2= Disatisfied<br>3= Neither satisfied nor dissatisfied<br>4= Satisfied<br>5= Very satisfied
320	How satisfied are you with your personal relationships?	1= Very dissatisfied<br>2= Disatisfied<br>3= Neither satisfied nor dissatisfied<br>4= Satisfied<br>5= Very satisfied
321	How satisfied are you with your sex life?	1= Very dissatisfied<br>2= Disatisfied<br>3= Neither satisfied nor dissatisfied<br>4= Satisfied<br>5= Very satisfied
322	How satisfied are you with the support you get from your friends?	1= Very dissatisfied<br>2= Disatisfied<br>3= Neither satisfied nor dissatisfied<br>4= Satisfied<br>5= Very satisfied

For peer review only

323	How satisfied are you with the conditions of your living place?	1= Very dissatisfied<br>2= Disatisfied<br>3= Neither satisfied nor dissatisfied<br>4= Satisfied<br>5= Very satisfied
324	How satisfied are you with your access to health services?	1= Very dissatisfied<br>2= Disatisfied<br>3= Neither satisfied nor dissatisfied<br>4= Satisfied<br>5= Very satisfied
325	How satisfied are you with your transport?	1= Very dissatisfied<br>2= Disatisfied<br>3= Neither satisfied nor dissatisfied<br>4= Satisfied<br>5= Very satisfied
326	How often do you have negative feelings such as blue mood, depair, anxiety or depression?	1= Never<br>2= Infrequently<br>3= Sometimes<br>4= Frequently<br>5= Always

For peer review only

For peer review only - http://bmjopen.bmj.com/site/about/guidelines.xhtml

SECTION 400: HEALTH CONDITIONS AND NUTRITION

I am now going to ask some questions about your health

Self-rated health

401	In general, how would you rate your health?	1= Good<br>2= Rather good<br>3= Poor<br>4= Very poor

Nutrition (WOPS)

402	How many servings of fruit do you eat on a typical day?	Servings
403	How many servings of vegetables do you eat on a typical day?	Servings
404	In the last 12 months, were you ever hungry, but didn't eat because you couldn't afford enough food?	1 = Yes<br>2 = No
405	In the last 12 months, how often did you eat less than you felt you should because there wasn't enough food?	1= every month<br>2= almost every month<br>3= some months. But not every month<br>4= only in 1 or 2 months<br>5= Never

HEALTH STATUS (Model disability survey)

	**Have you been told by a doctor or health professional that you have…**		**If yes, have you been given medicine or treatment in the last 6-12 months?**
**406**	Vision loss	1 = Yes<br>2 = No	
**407**	Hearing loss	1 = Yes<br>2 = No	
**408**	High blood pressure	1 = Yes<br>2 = No	
**409**	Diabetes	1 = Yes<br>2 = No	
**410**	Arthritis	1 = Yes<br>2 = No	
**411**	Heart disease, coronary disease, heart attack	1 = Yes<br>2 = No	
**412**	Chronic bronchitis or emphysema	1 = Yes	

For peer review only - http://bmjopen.bmj.com/site/about/guidelines.xhtml

		2 = No	
**413**	Asthma, allergic, respiratory disease	1 = Yes<br>2 = No	
**414**	Back pain or disc problems	1 = Yes<br>2 = No	
**415**	Migraine (recurrent headaches)	1 = Yes<br>2 = No	
**416**	Stroke	1 = Yes<br>2 = No	
**417**	Depression or anxiety	1 = Yes<br>2 = No	
**418**	Gastritis or ulcer	1 = Yes<br>2 = No	
**419**	Tumour or cancer	1 = Yes<br>2 = No	
**420**	Trauma	1 = Yes<br>2 = No	
**421**	Dementia	1 = Yes<br>2 = No	
**422**	Kidney disease	1 = Yes<br>2 = No	
**423**	Skin disease	1 = Yes<br>2 = No	
**424**	Tuberculosis	1 = Yes<br>2 = No	
**425**	Mental (psychiatric) or behavioural disorders	1 = Yes<br>2 = No	

For peer review only

Section 500: Health care utilization

I would now like to know about your recent experiences with obtaining health care from health care workers, hospitals, clinics and the health care system. I want to know if you needed health care recently, and if so, why you needed health care provider youreceived care from.

501	In the last 2 years did you need health care?	1=Yes<br>2=No
502	The last time you needed health care, did you get health care?<br>**If "yes" Skip to 504**	1= Yes<br>2= No
**Interviewer: if respondent did not get health care, Q 502= "NO", ask Q 503A, 503a,503b and 503I then Skip to question 511**		
503A	**Interviewer: Respondent can select ONLY one main reason for visit. Use showcard**<br>**If respondent is having difficulties answering, you can read the categories**<br>What was the main reason you needed care, even if you did not get care?	
	1= Communicable diseases, infections, malaria, infection TB, HIV	
	2= Nutritional deficiencies	
	3= Acute conditions, (diarrhea, flu, headaches, fever, cough and others)	
	4= Injury (if FALLS record 4 and fill in FALLS in 403a (SPECIFY)	
	5= Surgery	
	6= Sleep problem	
	7= Occupational /work related condition/injury	
	8= Chronic pain in joints/arthritis (joints, back, neck)	
	9= Diabetes or related complications	
	10= Problems with heart including unexplained pain in chest	
	11= Problems with mouth, teeth, swallowing	
	12= Problems with breathing	
	13= High blood pressure, hypertension	
	14= Stroke/ sudden paralysis of one side of body	

For peer review only - http://bmjopen.bmj.com/site/about/guidelines.xhtml

	15= Generalized pain(stomach, muscle or other nonspecific pain)	
	16= Depression, anxiety	
	17= Cancer	
	87= Other	
503a	Other main reason SPECIFY	______________________________
503b	Which reason(s) best explains why you did not get health care?<br><br>**Record all the numbers that respondent mentions spontaneously.**	
	1= could not afford the cost of the visit	
	2= no transport available	
	3= could not afford the cost of the transport	
	4= you were previously badly treated	
	5= could not make time off work or had other commitments	
	6= the health care provider's drugs or equipment were inadequate	
	7= the health care provider's skills were inadequate	
	8= you did not know where to go	
	9= you tried but were denied health care	
	10= you thought you were not sick enough	
	87= other	
503I	Other best reason why not get health care SPECIFY	______________________________<br>______________________________
504	Thinking about health care you needed since last visit / in the last 2 years, where did you go most often when you felt sick or needed to consult someone about your health?	1= Private doctor's office<br>2= Private clinic or health centre<br>3= Government / public clinic or health centre<br>4= Government / public hospital<br>5= Charity or church run clinic

For peer review only - http://bmjopen.bmj.com/site/about/guidelines.xhtml

	**Interviewer: <u>Only one answer</u> allowed**	(name specific ones)
		6= Charity or church run hospital (name specific ones)
		7= Traditional healers / herbalist /shrine
		8= pharmacy
		9= Pastor or religious healer
		87= other
504a	How many visits do you make to a health facility in a year?	Number ___________________
505	Did you have to pay fees for consultation and/or drugs?**If no skip to 507**	1= Yes<br>2= No
506	Who paid for the last visit?	Self
		Spouse
		Son/daughter
		Other relative
		Other not relative
		Insurance
		was free

Interviewer ask Q 507, 508,509, and 510 ONLY when respondent use health services check Q 504
If not using health service skip to 511

For peer review only - http://bmjopen.bmj.com/site/about/guidelines.xhtml

507	When you visit the health facility, how long do you usually have to wait before it is your turn to be seen by a nurse or doctor?	1= Not long<br>2= Quite long<br>3= Long<br>4= Very long
508	When you visit the health facility, do the health professionals usually give you the time to explain to them what your health problem is?	1= Always<br>2= Most of the time<br>3= Sometimes<br>4= Not usually<br>5= Never
509	When you visit the health facility, do the health professionals usually take the time to explain your health problem and treatment in a way that you understand?	1= Always<br>2= Most of the time<br>3= Sometimes<br>4= Not usually<br>5= Never
509a	When you visit the health facility, do the health professionals usually take time to involve you in making decisions for your treatment?[1]	1= Always<br>2= Most of the time<br>3= Sometimes<br>4= Not usually<br>5= Never
510	When you visit the health facility, are you treated respectfully?	1= Always<br>2= Most of the time<br>3= Sometimes<br>4= Not usually<br>5= Never
511	Overall, are you satisfied with the services?	1= Very satisfied<br>2= Satisfied<br>3= Neither satisfied nor dissatisfied<br>4= Dissatisfied<br>5= Very dissatisfied
512	What are the reason(s) that people will go to the traditional healer for treatment?<br>**TICK ALL THAT APPLY**	Closer distance
		Traditional healer are cheaper
		Traditional healer allow you to pay in kind/ with goods
		Traditional healer will wait for payment
		Traditional healer is more knowledgeable about this

[1] Question added from Model Disability Survey

For peer review only - http://bmjopen.bmj.com/site/about/guidelines.xhtml

		problem
		Traditional healers give better treatment
		Decision made by caregiver or family
512a	Have you visited a traditional healer for treatment? **If no skip to 513**	1= yes<br>2= no
512b	During the last 12 months/one year, how often have you visited a traditional healer?	1 = Once or twice<br>2 = Three to six times<br>3 = More than six times<br>8 = Don't know

Health centre/clinic, hospital stays

513	In the last two years have you stayed an overnight in a health centre/ health clinic/hospital?<br>**IF NO SKIP TO 5600**	1= Yes<br>2= No
514	How many times have you been hospitalized in the last 2 years?	Number of admissions<br>_____________
515	What type of hospital was it the last time you were hospitalized? Public, private or church run?	1 = Public hospital<br>2 = Private hospital<br>3 = Charity or church run hospital<br>87 = Other Specify
**516** .	**RECORD REASON HOSPITALISATION**<br>**ONLY ONE REASON**<br>Which reason best describes why you were last hospitalized?<br><br>1= Communicable diseases, infections, malaria, infection TB, HIV<br>2= Nutritional deficiencies<br>3= Acute conditions, (diarrhea, flu, headaches, fever, cough and others)<br>4= Injury (if FALLS record 4 and fill in FALLS in 403a (SPECIFY)<br>5= Surgery<br>6= Sleep problem<br>7= Occupational /work related condition/injury<br>8= Chronic pain in joints/arthritis (joints, back, neck)<br>9= Diabetes or related complications<br>10= Problems with heart including unexplained pain in chest<br>11= Problems with mouth, teeth, swallowing<br>12= Problems with breathing<br>13= High blood pressure, hypertension<br>14= Stroke/ sudden paralysis of one side of body	

For peer review only - http://bmjopen.bmj.com/site/about/guidelines.xhtml

	15= Generalized pain(stomach, muscle or other nonspecific pain)<br>16= Depression, anxiety<br>17= Cancer<br>87= Other	
516a	Other best reason respondent was hospitalized	______________
517	Who paid for this hospitalization?	Self
		Spouse
		Son/daughter
		Other relative
		Insurance
		was free
		Other
517a	Other paid hospitalization specify	
518	What was the outcome or result of your stay in the health centre/ health clinic/hospital? Did your condition…	1= Get much better<br>2= Get better<br>3= No change<br>4= Get worse<br>5= Get much worse
519	Was this the outcome or results you had expected?	1= yes<br>2= no

For peer review only - http://bmjopen.bmj.com/site/about/guidelines.xhtml

SECTION 600: Participation (SINTEF)

I'm going to ask you some questions about your involvement in different aspects of family, social life and society. Please listen to each one and answer yes, no, sometimes, sometimes or not applicable.

Q. #	Question	Codes					Go to Q
		Yes	No	Sometimes	N/A	Don't know	
601	Are you consulted about making household decisions?	1	2	3	4	5	
602	Do you go with the family to events such as family gatherings, weddings, thanksgivings, funerals and birthdays, and other social functions?	1	2	3	4	5	
603	Do you feel involved and part of the household or family?	1	2	3	4	5	
604	Does the family involve you in conversations?	1	2	3	4	5	
605	Does the family help you with daily activities/tasks?	1	2	3	4	5	"No, DK, N/A" →607
606	Do you appreciate it or like the fact that you get this help?	1	2	3	4	5	
607	Do you/did you take part in your own traditional practices (e.g initiation ceremonies)?	1	2	3	4	5	
608	Are you aware of organizations for people with disabilities (DPO)?	1	2				"No" →610
609	Are you a member of a DPO?	1	2				
610	Do you participate in local community meetings?	1	2	3	4	5	"No" →612
611	Do you feel your voice is being heard?	1	2			5	
612	Did you vote in the last election?	1	2			5	**"No"→ sect 700**
613	Was it related to your disability that you didn't vote?	1	2			5	

For peer review only - http://bmjopen.bmj.com/site/about/guidelines.xhtml

Section 700: Attitudes (Model Disability Survey)

Now I want to ask you some questions about the attitudes of people around you. When answering these questions please tell me on a scale from 1 to 5, where 1 is not at all and 5 means completely.

Q. #	Question	Codes					
		1 Not at all	2	3	4	5 Yes, complete ly	98 Not applicable
701	Do you have problems getting involved in society because of the attitudes of others?	1	2	3	4	5	98
702	Do you feel that some people treat you unfairly?	1	2	3	4	5	98
703	Do you make your own choices about your day-to-day life? For example, where you go, what to eat.	1	2	3	4	5	98
704	Do you feel other people accept you?	1	2	3	4	5	98
705	Do you feel that other people respect you? For example, do you feel that others value you as a person and listen to what you have to say?	1	2	3	4	5	98
706	Do you consider yourself a burden on society?	1	2	3	4	5	98
707	Do people around you tend to become impatient with you?	1	2	3	4	5	98
708	Do people around you not expect much from you?	1	2	3	4	5	98
709	Is living with dignity a problem for you because of the attitudes and actions of others?	1	2	3	4	5	98

For peer review only

SECTION 800: PLA-D

BASELINE only

Now I would like to ask you some questions about your needs and expectations of the programme

Nr.		CODE
801	What are the three biggest issues that in your day-to-day life that you find difficult or need support with?	
802	What are the three biggest issues related to your health you find difficult or need support with?	
803	What are your main goals for attending this group?	

END-LINE only

Now I would like to ask you some questions about your feelings about the programme?

Remind the participant of their goals from the baseline

Nr.		CODE
804	Do you feel that you have achieved or met your goals?	1=Not at all<br>2= Partially<br>3= Fully
805	Please explain	
806	How satisfied were you with the content of the group programme?	1= Very dissatisfied<br>2= Disatisfied<br>3= Neither satisfied nor dissatisfied<br>4= Satisfied<br>5= Very satisfied
807	How satisfied were you with the organization of the programme (e.g. transport, location, refreshments)?	1= Very dissatisfied<br>2= Disatisfied<br>3= Neither satisfied nor dissatisfied<br>4= Satisfied<br>5= Very satisfied
808	How satisfied were you with the facilitators of the programme?	1= Very dissatisfied<br>2= Disatisfied<br>3= Neither satisfied nor dissatisfied<br>4= Satisfied<br>5= Very satisfied
809	Can you suggest ways in which the	

For peer review only - http://bmjopen.bmj.com/site/about/guidelines.xhtml

	programme could be improved?	

810	**RECORD THE TIME**	___ Hrs __Min	TIMEPART2END

Closing statement and thank the respondent
INTERVIEWER: This is the end of the interview
This completes the interview. We thank you for your time and answers. I have your contact details and may be in touch again.

For peer review only

For peer review only - http://bmjopen.bmj.com/site/about/guidelines.xhtml

MRC/UVRI & LSHTM Uganda Research Unit **and LSHTM**

Questionnaire: Child version (aged 5-17)

Table of contents

Section 100: Respondent and household characteristics
Section 200: Washington Group Short Set Enhanced
Section 300: Quality of life
Section 400: Health condition and nutrition
Section 500: Health care utilization
Section 600: Participation
Section 700: Attitudes
Section 800: PLA-D specific questions

Nr.		
001	LOCATION ID NUMBER	________________
002	**ID NUMBER**	________________
003	**INTERVIEW TYPE**	1=Person with disabilities (direct)<br>2=Proxy for person with disabilities (unable to communicate/understand)<br>2=Proxy for person with disabilities (child<18)<br>________________
004	**Interviewer 1 initials and code**	
005	**Date of interview**	__ __ 2023<br>(DAY MONTH)
006	**Time start interview**	__ hrs __ min
007	**Time end interview**	__ hrs __ min

For peer review only - http://bmjopen.bmj.com/site/about/guidelines.xhtml

SECTION 100 - RESPONDENT AND HOUSEHOLD CHARACTERISTICS (WOPS)

I am going to start by asking you some questions about your child and his/her living situation.

101	SEX	1= Male<br>2= Female
102	How old is [NAME] now?	Years……..
103	In what year was [NAME] born?<br>**IF KNOWN INCLUDE DATE OF BIRTH; CORRECT INCONSISTENCIES WITH AGE**	Year of birth….
		Date of birth<br>…../……/……….
104	Has [NAME] ever attended school or any early childhood education programme	1=Yes<br>2=No
105	What is the highest grade of school [NAME] has ever attended?	1=Early childhood education<br>2=Primary<br>3=Lower secondary<br>4=Upper secondary<br>5=Higher
106	At any time during the current school year did [NAME] attend school of any early childhood education programme?	1=Yes<br>2=No
107	During this current school year, what grade of school is [NAME] attending?	1=Early childhood education<br>2=Primary<br>3=Lower secondary<br>4=Upper secondary<br>5=Higher
108	What is the occupation of your head of household? Any other?<br>**TICK ALL THAT APPLY**	House work
		Farming
		Fishing
		Labouring (e.g. clearing streets, weeding)
		Tailoring
		Teacher / nurse
		Government administration
		Shop attendant
		other,
109	What is head of	1= No formal education

For peer review only - http://bmjopen.bmj.com/site/about/guidelines.xhtml

	household's highest level of education?	2= Less than primary (incomplete) school<br>3= Completed primary school<br>4= complete O level<br>5= Completed A level<br>6= Higher education than secondary school (diploma course or BTVET)<br>7= Adult education only<br>8= College/ University or more<br>88= Don't know
110	What is the total number of people who live in your household?	[ ][ ] Persons

LIVING CONDITIONS

111	What is the predominant material used for roofing?	1= Thatch<br>2= Tile<br>3= Iron sheets<br>4= Mixed iron/tile<br>5= Other
112	What is the state of the roof?	1= Very Good<br>1= Good<br>2= Moderate<br>3= Bad<br>4= Very bad
113	What are the walls of the house made of?	1= Burned brick<br>2= Unburned brick<br>3= Mud
114	What is the house floor made of?	1= Mud<br>2= Cement<br>3= Tiles
115	Who owns the house?	1= Self<br>2= Spouse<br>3= Respondent's children<br>4= Someone else in the household<br>5= Renting (landlord)<br>87= Other

For peer review only - http://bmjopen.bmj.com/site/about/guidelines.xhtml

SECTION 200: Washington Group Child Functioning Set
I would like to ask you some questions about difficulties [NAME] may have

201	Does [NAME] wear glasses or contact lenses?	1=Yes<br>2=No	No – skip to 203
202	When wearing his/her glasses, does [NAME] have difficulty seeing? Would you say… [read response categories]	1=No difficulty<br>2=Some difficulty<br>3= A lot of difficulty<br>4= Cannot do	
203	Does [NAME] have difficulty seeing? Would you say… [read response categories]	1=No difficulty<br>2=Some difficulty<br>3= A lot of difficulty<br>4= Cannot do	
204	Does [NAME] use a hearing aid?	1=Yes<br>2=No	No – skip to 206
205	When using his/her hearing aid, does [NAME] have difficulty hearing sounds like people's voices or music? Would you say… [read response categories]	1=No difficulty<br>2=Some difficulty<br>3= A lot of difficulty<br>4= Cannot do	
206	Does [NAME] have difficulty hearing sounds like people's voices or music? Would you say… [read response categories]	1=No difficulty<br>2=Some difficulty<br>3= A lot of difficulty<br>4= Cannot do	
207	Does [NAME] use any equipment or receive assistance for walking?	1=Yes<br>2=No	No – skip to 212
208	Without his/her equipment or assistance, does [NAME] have difficulty walking 100 yards/meters on level ground? That would be about the length of 1 football field. Would you say… [read response categories]	1=No difficulty<br>2=Some difficulty<br>3= A lot of difficulty<br>4= Cannot do	
209	Without (his/her) equipment or assistance, does [NAME] have difficulty walking 500 meters/yards on level ground? That would be about the length of 5 football fields.	1=No difficulty<br>2=Some difficulty<br>3= A lot of difficulty<br>4= Cannot do	
210	With (his/her) equipment or assistance, does [NAME] have difficulty walking 100 meters/yards on level ground? That would be about the length of 1 football field.	1=No difficulty<br>2=Some difficulty<br>3= A lot of difficulty<br>4= Cannot do	
211	With (his/her) equipment or assistance, does [NAME] have difficulty walking 500 meters/yards on level ground? That would be about the length of 5 football fields.	1=No difficulty<br>2=Some difficulty<br>3= A lot of difficulty<br>4= Cannot do	
212	Compared with children of the same age, does [NAME] have difficulty walking 100 meters/yards	1=No difficulty<br>2=Some difficulty	

For peer review only - http://bmjopen.bmj.com/site/about/guidelines.xhtml

	on level ground? That would be about the length of 1 football field.	3= A lot of difficulty<br>4= Cannot do	
213	Compared with children of the same age, does [NAME] have difficulty walking 500 meters/yards on level ground? That would be about the length of 5 football fields.	1=No difficulty<br>2=Some difficulty<br>3= A lot of difficulty<br>4= Cannot do	
214	Does [NAME] have difficulty with self-care such as feeding or dressing (himself/herself)?	1=No difficulty<br>2=Some difficulty<br>3= A lot of difficulty<br>4= Cannot do	
215	When **[NAME]** speaks, does (he/she) have difficulty being understood by people inside of this household?	1=No difficulty<br>2=Some difficulty<br>3= A lot of difficulty<br>4= Cannot do	
216	When [NAME] speaks, does (he/she) have difficulty being understood by people outside of this household?	1=No difficulty<br>2=Some difficulty<br>3= A lot of difficulty<br>4= Cannot do	
217	Compared with children of the same age, does [NAME] have difficulty learning things?	1=No difficulty<br>2=Some difficulty<br>3= A lot of difficulty<br>4= Cannot do	
218	Compared with children of the same age, does [NAME] have difficulty remembering things?	1=No difficulty<br>2=Some difficulty<br>3= A lot of difficulty<br>4= Cannot do	
219	Does [NAME] have difficulty concentrating on an activity that (he/she) enjoys doing?	1=No difficulty<br>2=Some difficulty<br>3= A lot of difficulty<br>4= Cannot do	
220	Does [NAME] have difficulty accepting changes in (his/her) routine?	1=No difficulty<br>2=Some difficulty<br>3= A lot of difficulty<br>4= Cannot do	
221	Compared with children of the same age, does [NAME] have difficulty controlling (his/her) behaviour?	1=No difficulty<br>2=Some difficulty<br>3= A lot of difficulty<br>4= Cannot do	
222	Does [NAME] have difficulty making friends?	1=No difficulty<br>2=Some difficulty<br>3= A lot of difficulty<br>4= Cannot do	
	The next questions have different options for answers. I am going to read these to you after each question.		

For peer review only

223	I would like to know how often [NAME] seems very anxious, nervous or worried.<br><br>Would you say: daily, weekly, monthly, a few times a year or never?	1=Daily<br>2=Weekly<br>3=Monthly<br>4=A few times a year<br>5=Never	
224	I would also like to know how often [NAME] seems very sad or depressed.<br><br>Would you say: daily, weekly, monthly, a few times a year or never?	1=Daily<br>2=Weekly<br>3=Monthly<br>4=A few times a year<br>5=Never	

For peer review only

For peer review only - http://bmjopen.bmj.com/site/about/guidelines.xhtml

SECTION 300 QUALITY OF LIFE (KIDSCREEN-PARENT)

301	In general, how would you say your child's health is?	1=Poor<br>2=Fair<br>3=Good<br>4=Very good<br>5=Excellent
	Over the past 2 weeks….	
302	Has your child felt fit and well?	1=Not at all<br>2=Slightly<br>3=Moderately<br>4=Very<br>5=Extremely
303	Has your child felt full of energy?	1=Never<br>2=Seldom<br>3=Quite often<br>4=Very often<br>5=Always
304	Has your child felt sad?	1=Never<br>2=Seldom<br>3=Quite often<br>4=Very often<br>5=Always
305	Has your child felt lonely?	1=Never<br>2=Seldom<br>3=Quite often<br>4=Very often<br>5=Always
306	Has your child had enough time for him/herself?	1=Never<br>2=Seldom<br>3=Quite often<br>4=Very often<br>5=Always
307	Has your child been able to do the things he/she wants to do in his/her free time?	1=Never<br>2=Seldom<br>3=Quite often<br>4=Very often<br>5=Always

For peer review only

308	Has your child felt that his/her parent(s) treated him/her fairly?	1=Never<br>2=Seldom<br>3=Quite often<br>4=Very often<br>5=Always
309	Has your child had fun with his/her friends?	1=Never<br>2=Seldom<br>3=Quite often<br>4=Very often<br>5=Always
310	Has your child been able to pay attention?	1=Never<br>2=Seldom<br>3=Quite often<br>4=Very often<br>5=Always
311	Has your child got on well at school?	1=Not at all<br>2=Slightly<br>3=Moderately<br>4=Very<br>5=Extremely

For peer review only

SECTION 400: HEALTH CONDITIONS AND NUTRITION
I am now going to ask some questions about your child's health

Nutrition (WOPS)

402	How many servings of fruit does [NAME] eat on a typical day?	Servings
403	How many servings of vegetables does [NAME]eat on a typical day?	Servings
404	In the last 12 months, did [NAME] ever hungry, but didn't eat because you couldn't afford enough food?	1 = Yes<br>2 = No
405	In the last 12 months, how often did [NAME] eat less than you felt [NAME] should because there wasn't enough food?	1= every month<br>2= almost every month<br>3= some months. But not every month<br>4= only in 1 or 2 months<br>5= Never

For peer review only

HEALTH STATUS (Model disability survey)

	**Has [NAME] been told by a doctor or health professional that he/she has…**		**If yes, has [NAME] been given medicine or treatment in the last 6-12 months?**
**406**	Vision loss	1 = Yes<br>2 = No	
**407**	Hearing loss	1 = Yes<br>2 = No	
**408**	High blood pressure	1 = Yes<br>2 = No	
**409**	Diabetes	1 = Yes<br>2 = No	
**410**	Arthritis	1 = Yes<br>2 = No	
**411**	Heart disease, coronary disease, heart attack	1 = Yes<br>2 = No	
**412**	Chronic bronchitis or emphysema	1 = Yes<br>2 = No	
**413**	Asthma, allergic, respiratory disease	1 = Yes<br>2 = No	
**414**	Back pain or disc problems	1 = Yes<br>2 = No	
**415**	Migraine (recurrent headaches)	1 = Yes	

For peer review only - http://bmjopen.bmj.com/site/about/guidelines.xhtml

		2 = No	
**416**	Stroke	1 = Yes<br>2 = No	
**417**	Depression or anxiety	1 = Yes<br>2 = No	
**418**	Gastritis or ulcer	1 = Yes<br>2 = No	
**419**	Tumour or cancer	1 = Yes<br>2 = No	
**420**	Trauma	1 = Yes<br>2 = No	
**421**	Dementia	1 = Yes<br>2 = No	
**422**	Kidney disease	1 = Yes<br>2 = No	
**423**	Skin disease	1 = Yes<br>2 = No	
**424**	Tuberculosis	1 = Yes<br>2 = No	
**425**	Mental (psychiatric) or behavioural disorders	1 = Yes<br>2 = No	

For peer review only

For peer review only - http://bmjopen.bmj.com/site/about/guidelines.xhtml

Section 500: Health care utilization

I would now like to know about your recent experiences with obtaining health care for your child from health care workers, hospitals, clinics and the health care system. I want to know if your child needed health care recently, and if so, why you needed health care provider you received care from.

501	In the last 2 years did your child need health care?	1=Yes<br>2=No
502	The last time your child needed health care, did you get health care?<br>**If "yes" Skip to 504**	1= Yes<br>2= No
**Interviewer: if respondent did not get health care, Q 502= "NO", ask Q 503A, 503a,503b and 503I then Skip to question 511**		
503A	**Interviewer: Respondent can select ONLY one <u>main reason</u> for visit. Use showcard**<br>**If respondent is having difficulties answering, you can read the categories**<br>What was the main reason your child needed care, even if your child did not get care?	
	1= Communicable diseases, infections, malaria, infection TB, HIV	
	2= Nutritional deficiencies	
	3= Acute conditions, (diarrhea, flu, headaches, fever, cough and others)	
	4= Injury (if FALLS record 4 and fill in FALLS in 403a (SPECIFY)	
	5= Surgery	
	6= Sleep problem	
	7= Occupational /work related condition/injury	
	8= Chronic pain in joints/arthritis (joints, back, neck)	
	9= Diabetes or related complications	
	10= Problems with heart including unexplained pain in chest	
	11= Problems with mouth, teeth, swallowing	
	12= Problems with breathing	
	13= High blood pressure, hypertension	

For peer review only

	14= Stroke/ sudden paralysis of one side of body	
	15= Generalized pain(stomach, muscle or other nonspecific pain)	
	16= Depression, anxiety	
	17= Cancer	
	87= Other	
503a	Other main reason SPECIFY	___________________________
503b	Which reason(s) best explains why your child did not get health care?<br><br>**Record all the numbers that respondent mentions spontaneously.**	
	1= could not afford the cost of the visit	
	2= no transport available	
	3= could not afford the cost of the transport	
	4= your child was previously badly treated	
	5= could not make time off work or had other commitments	
	6= the health care provider's drugs or equipment were inadequate	
	7= the health care provider's skills were inadequate	
	8= you did not know where to go	
	9= you tried but were denied health care	
	10= you thought your child was not sick enough	
	87= other	
503I	Other best reason why not get health care SPECIFY	___________________________<br><br>___________________________
504	Thinking about health care your child needed since last visit / in the last 2 years, where did your child go most often when he/she felt sick or needed to consult someone about health?	1= Private doctor's office<br>2= Private clinic or health centre<br>3= Government / public clinic or health centre<br>4= Government / public hospital<br>5= Charity or church run clinic

For peer review only

For peer review only - http://bmjopen.bmj.com/site/about/guidelines.xhtml

	**Interviewer: <u>Only one answer</u> allowed**	(name specific ones)
		6= Charity or church run hospital (name specific ones)
		7= Traditional healers / herbalist /shrine
		8= pharmacy
		9= Pastor or religious healer
		87= other
504a	How many visits do your child make to a health facility in a year?	Number ___________________
505	Did you have to pay fees for consultation and/or drugs?**If no skip to 507**	1= Yes<br>2= No
506	Who paid for the last visit?	Self (i.e. parent being interviewed)
		Spouse
		Son/daughter
		Other relative
		Other not relative
		Insurance
		was free

Interviewer ask Q 507, 508,509, and 510 ONLY when respondent use health services check Q 504
If not using health service skip to 511

For peer review only - http://bmjopen.bmj.com/site/about/guidelines.xhtml

507	When you visit the health facility, how long do you usually have to wait before it is your child's turn to be seen by a nurse or doctor?	1= Not long<br>2= Quite long<br>3= Long<br>4= Very long
508	When you visit the health facility, do the health professionals usually give you the time to explain to them what your child's health problem is?	1= Always<br>2= Most of the time<br>3= Sometimes<br>4= Not usually<br>5= Never
509	When you visit the health facility, do the health professionals usually take the time to explain your child's health problem and treatment in a way that you understand?	1= Always<br>2= Most of the time<br>3= Sometimes<br>4= Not usually<br>5= Never
509a	When you visit the health facility, do the health professionals usually take time to involve you/your child in making decisions for your treatment?[1]	1= Always<br>2= Most of the time<br>3= Sometimes<br>4= Not usually<br>5= Never
510	When you visit the health facility, is your child treated respectfully?	1= Always<br>2= Most of the time<br>3= Sometimes<br>4= Not usually<br>5= Never
511	Overall, are you satisfied with the services?	1= Very satisfied<br>2= Satisfied<br>3= Neither satisfied nor dissatisfied<br>4= Dissatisfied<br>5= Very dissatisfied
512	What are the reason(s) that people will go to the traditional healer for treatment?<br>**TICK ALL THAT APPLY**	Closer distance
		Traditional healer are cheaper
		Traditional healer allow you to pay in kind/ with goods
		Traditional healer will wait for payment
		Traditional healer is more knowledgeable about this

[1] Question added from Model Disability Survey

For peer review only - http://bmjopen.bmj.com/site/about/guidelines.xhtml

		problem
		Traditional healers give better treatment
		Decision made by caregiver or family
512a	Have you visited a traditional healer for treatment for your child?<br>**If no skip to 513**	1= yes<br>2= no
512b	During the last 12 months/one year, how often have you visited a traditional healer for treatment for your child?	1 = Once or twice<br>2 = Three to six times<br>3 = More than six times<br>8 = Don't know

Health centre/clinic, hospital stays

513	In the last two years has your child stayed an overnight in a health centre/ health clinic/hospital?<br>**IF NO SKIP TO 5600**	1= Yes<br>2= No
514	How many times has your child been hospitalized in the last 2 years?	Number of admissions<br>_____________
515	What type of hospital was it the last time your child was hospitalized? Public, private or church run?	1 = Public hospital<br>2 = Private hospital<br>3 = Charity or church run hospital<br>87 = Other Specify

For peer review only

**516.**	**RECORD REASON HOSPITALISATION**<br>**ONLY ONE REASON**<br>Which reason best describes why your child was last hospitalized?<br><br>1= Communicable diseases, infections, malaria, infection TB, HIV<br>2= Nutritional deficiencies<br>3= Acute conditions, (diarrhea, flu, headaches, fever, cough and others)<br>4= Injury (if FALLS record 4 and fill in FALLS in 403a (SPECIFY)<br>5= Surgery<br>6= Sleep problem<br>7= Occupational /work related condition/injury<br>8= Chronic pain in joints/arthritis (joints, back, neck)<br>9= Diabetes or related complications<br>10= Problems with heart including unexplained pain in chest<br>11= Problems with mouth, teeth, swallowing<br>12= Problems with breathing<br>13= High blood pressure, hypertension<br>14= Stroke/ sudden paralysis of one side of body<br>15= Generalized pain(stomach, muscle or other nonspecific pain)<br>16= Depression, anxiety<br>17= Cancer<br>87= Other	
516a	Other best reason child was hospitalized	______________
517	Who paid for this hospitalization?	Self<br>Spouse<br>Son/daughter<br>Other relative<br>Insurance<br>was free<br>Other
517a	Other paid hospitalization specify	
518	What was the outcome or result of your child's stay in the health centre/ health clinic/hospital? Did his/her condition…	1= Get much better<br>2= Get better<br>3= No change<br>4= Get worse<br>5= Get much worse
519	Was this the outcome or results you had expected?	1= yes<br>2= no

For peer review only - http://bmjopen.bmj.com/site/about/guidelines.xhtml

SECTION 600: Participation (CASP)

We are interested in finding out about the activities that your child participates in at home, school and in the community. You will be asked about your child's current level of participation with activities as compared to others their age. For each item, choose one of the following:

Full participation, your child participates in the activities the same as or more than others his/her age. [*With or without assistive devices or equipment*]

Somewhat limited, your child participates in the activities somewhat less than others his/her age. [*He/she may also need occasional supervision or assistance*]

Very limited, your child participates in the activities much less than others his/her age. [*He/she may also need a lot of supervision or assistance*]

Unable, your child cannot participate in the activities, although others his/her age do participate.

Not applicable, others your child's age would not be expected to participate in the activities.

	Compared to other children of your child's age, what is your child's current level of participation in the following activities<br><br>HOME PARTICIPATION	
601	Social, play or leisure activities with family members at home (e.g., games, hobbies, "hanging out")	1=Full participation<br>2=Somewhat limited<br>3=Very limited<br>4=Unable<br>5=Not applicable
602	Social, play or leisure activities with friends at home	1=Full participation<br>2=Somewhat limited<br>3=Very limited<br>4=Unable<br>5=Not applicable
603	Family chores, responsibilities and decisions at home (e.g., involvement in household chores and decisions about plans)**)**	1=Full participation<br>2=Somewhat limited<br>3=Very limited<br>4=Unable<br>5=Not applicable
604	Self-care activities (e.g. eating, dressing, bathing, combing or brushing hair, using the toilet)	1=Full participation<br>2=Somewhat limited<br>3=Very limited<br>4=Unable

For peer review only - http://bmjopen.bmj.com/site/about/guidelines.xhtml

		5=Not applicable
605	Moving about in and around the home	1=Full participation<br>2=Somewhat limited<br>3=Very limited<br>4=Unable<br>5=Not applicable
606	Communicating with others at home	1=Full participation<br>2=Somewhat limited<br>3=Very limited<br>4=Unable<br>5=Not applicable
	NEIGHBOURHOOD & COMMUNITY PARTICIPATION	
607	Social, play, or leisure activities with friends in the neighbourhood and community (e.g., casual games, "hanging out," going to public places like the market, or church)	1=Full participation<br>2=Somewhat limited<br>3=Very limited<br>4=Unable<br>5=Not applicable
608	Structured events and activities in the neighbourhood and community (e.g. football, church or mosque, community meetings, and family functions)	1=Full participation<br>2=Somewhat limited<br>3=Very limited<br>4=Unable<br>5=Not applicable
609	Moving around the neighbourhood and community (e.g., public buildings, taxi's, market) [*Please consider your child's primary way of moving around, NOT your use of transportation*]	1=Full participation<br>2=Somewhat limited<br>3=Very limited<br>4=Unable<br>5=Not applicable
610	Communicating with others in the neighbourhood and community	1=Full participation<br>2=Somewhat limited<br>3=Very limited<br>4=Unable<br>5=Not applicable
	SCHOOL PARTICIPATION	
611	Educational (academic) activities with other pupils in your child's classroom at school	1=Full participation<br>2=Somewhat limited<br>3=Very limited<br>4=Unable<br>5=Not applicable
612	Social, play and recreational activities with other pupils at school (e.g., "hanging out," sports, creative arts, lunchtime or recess)	1=Full participation<br>2=Somewhat limited<br>3=Very limited<br>4=Unable<br>5=Not applicable
613	Moving around at school (e.g., to get to and use bathroom, playground, lunch area, library or other rooms and things that are available to other pupils your child's age)	1=Full participation<br>2=Somewhat limited<br>3=Very limited

For peer review only - http://bmjopen.bmj.com/site/about/guidelines.xhtml

		4=Unable<br>5=Not applicable
614	Using educational materials and equipment that are available to other pupils in your child's classroom/s or that have been modified for your child (e.g., books, chairs and desks)	1=Full participation<br>2=Somewhat limited<br>3=Very limited<br>4=Unable<br>5=Not applicable
615	Communicating with other pupils and adults at school	1=Full participation<br>2=Somewhat limited<br>3=Very limited<br>4=Unable<br>5=Not applicable
	DAILY LIVING ACTIVITIES	
616	Household activities (e.g., preparing meals, doing laundry, washing dishes)	1=Full participation<br>2=Somewhat limited<br>3=Very limited<br>4=Unable<br>5=Not applicable
617	Shopping and managing money (e.g., shopping at market, figuring out correct change)	1=Full participation<br>2=Somewhat limited<br>3=Very limited<br>4=Unable<br>5=Not applicable
618	Managing daily schedule (e.g. doing and completing daily activities on time; getting ready for school and doing homework)	1=Full participation<br>2=Somewhat limited<br>3=Very limited<br>4=Unable<br>5=Not applicable
619	Using transportation to get around in the community (e.g. to and from school, work, social or leisure activities) [*using taxi and body*]	1=Full participation<br>2=Somewhat limited<br>3=Very limited<br>4=Unable<br>5=Not applicable
620	School activities and responsibilities (e.g., completion of tasks, punctuality, attendance and getting along with teachers)	1=Full participation<br>2=Somewhat limited<br>3=Very limited<br>4=Unable<br>5=Not applicable

For peer review only - http://bmjopen.bmj.com/site/about/guidelines.xhtml

SECTION 700: PLA-D

BASELINE only

Now I would like to ask you some questions about your needs and expectations of the programme

Nr.		CODE
701	What are the three biggest issues in [NAME]'s day-to-day life that you/your child find difficult or need support with?	
702	What are the three biggest issues related to [NAME]'s health you/your child find difficult or need support with?	
703	What are your main goals for attending this group?	

END-LINE only

Now I would like to ask you some questions about your feelings about the programme?

Remind the participant of their goals from the baseline

Nr.		CODE
804	Do you feel that you have achieved or met your goals?	1=Not at all<br>2= Partially<br>3= Fully
805	Please explain	
806	How satisfied were you with the content of the group programme?	1= Very dissatisfied<br>2= Disatisfied<br>3= Neither satisfied nor dissatisfied<br>4= Satisfied<br>5= Very satisfied
807	How satisfied were you with the organization of the programme (e.g. transport, location, refreshments)?	1= Very dissatisfied<br>2= Disatisfied<br>3= Neither satisfied nor dissatisfied<br>4= Satisfied<br>5= Very satisfied
808	How satisfied were you with the facilitators of the programme?	1= Very dissatisfied<br>2= Disatisfied<br>3= Neither satisfied nor dissatisfied<br>4= Satisfied<br>5= Very satisfied

For peer review only - http://bmjopen.bmj.com/site/about/guidelines.xhtml

809	Can you suggest ways in which the programme could be improved?	

810	**RECORD THE TIME**	___ Hrs __Min	TIMEPART2END

Closing statement and thank the respondent

INTERVIEWER: This is the end of the interview

This completes the interview. We thank you for your time and answers. I have your contact details and may be in touch again.

For peer review only

MRC Medical Research Council
Uganda Virus Research Institute
LONDON SCHOOL of HYGIENE &TROPICAL MEDICINE

Focus Group Discussion for Participants of PLA-D

Pilot-testing a participatory approach to improve access to healthcare for people with disabilities in Uganda

Objective: To gather information to help further design a participatory approach for people with disabilities to improve health

These questions should be used to guide discussion but do not have to be used in the sequence listed below. The interviewer should follow up on any additional issues that may arise and seem important in relation to the issues above.

Introduction

- Greet them and thank them for their time
- Identify yourself by name and organisation.
- Read out the information sheet. Remind them of confidentiality and anonymity. Check if they have any questions. Remind them that they are free to decline to answer any of the questions or stop the interview at any time.
- Record their consent/assent in the relevant form OR record verbal consent.
- Start recording

Notes: the following details must be recorded in field notes

Participant Code	
Interview date and time	
Interview location or mode (phone, video)	
Interviewer	
Interviewee number	
Gender (number men/women)	
Age (number in broad groups)	
General observations (anything which might impact how the interview is conducted)	

As we have discussed, we are gathering information to help improve the design of the groups about disability that you recently participated in.

1. Share with me your experience participating in the PLA-D groups?
2. What do you think was the purpose of the groups?

For peer review only

3. What did you think about the way the groups were organised?

   Prompt – facilitation, regularity of meetings, location, group facilitator, frequency

4. Are there any sessions that were particularly interesting and important for you, and if so why?

5. What, if any, do you think were the main benefits of the groups?

6. What, if any, were the main problems with the groups?

Prompt – gender, age, different forms of disability, date, location and timing of meetings

7. If we were to plan the groups again, what could we do differently?

8. Is there anything else that you would like to say about the groups that we have not covered?

For peer review only

MRC/UVRI and LSHTM Uganda Research Unit

Qualitative Interview guide for facilitators of PLA-D

Pilot-testing a participatory approach to improve access to healthcare for people with disabilities in Uganda

Objective: To gather information to help further design a participatory approach for people with disabilities to improve health

These questions should be used to guide discussion but do not have to be used in the sequence listed below. The interviewer should follow up on any additional issues that may arise and seem important in relation to the issues above.

Introduction

- Greet them and thank them for their time
- Identify yourself by name and organisation.
- Read out the information sheet. Remind them of confidentiality and anonymity. Check if they have any questions. Remind them that they are free to decline to answer any of the questions or stop the interview at any time.
- Record their consent/assent in the relevant form OR record verbal consent.
- Start recording

Notes: the following details must be recorded in field notes

Participant Code	
Interview date and time	
Interview location or mode (phone, video, in person)	
Interviewer	
Gender	
Age	
Profession	
General observations (anything which might impact how the interview is conducted)	

As we have discussed, we are gathering information to help improve the design of the groups about disability that you recently facilitated.

1. Share with me your experience being a group facilitator.
2. Can you tell me why you decided to become a group facilitator?

For peer review only

MRC/UVRI and LSHTM Uganda Research Unit

3. What were your initial thoughts of the group and being a group facilitator?

4. What do you think was the purpose of the groups?

5. How was the group attendance at the group? Why do you think people did not come?

6. Can you tell me more about the people in the group? Were there men and women? What about different ages and types of impairment? Were there people with disabilities and caregivers? Who else came?

7. What did you think about the way the groups were organised?

   Prompt – facilitation, regularity of meetings, location,

8. What do you think about the type and level of support that you received as a group facilitator?

9. Are there any sessions that were particularly important or interesting for you, and if so why?

10. What, if any, were the main benefits you perceived of the groups?

11. What actions were taken through or by the groups? What do you think were their effects?

12. Can you describe any changes that you have perceived in your life because of the groups? And in the lives of the group members?

13. What, if any, were the main problems with the groups?

14. Can you think of what any suggestions or feedback would be to someone who as thinking of establishing PLA-D groups?

15. If we were to plan the groups again, what could we do differently?

16. Is there anything else that you would like to say about the groups that we have not covered?

For peer review only

MRC/UVRI and LSHTM Uganda Research Unit

Qualitative Interview guide for people who did not participate in PLA-D

Pilot-testing a participatory approach to improve access to healthcare for people with disabilities in Uganda

Objective: To gather information to help further design a participatory approach for people with disabilities to improve health

These questions should be used to guide discussion but do not have to be used in the sequence listed below. The interviewer should follow up on any additional issues that may arise and seem important in relation to the issues above.

Introduction

- Greet them and thank them for their time
- Identify yourself by name and organisation.
- Read out the information sheet. Remind them of confidentiality and anonymity. Check if they have any questions. Remind them that they are free to decline to answer any of the questions or stop the interview at any time.
- Record their consent/assent in the relevant form OR record verbal consent.
- Start recording

Notes: the following details must be recorded in field notes

Participant Code	
Interview date and time	
Interview location or mode (phone, video, in person)	
Interviewer	
Gender	
Age	
Profession	
General observations (anything which might impact how the interview is conducted)	

As we have discussed, we are gathering information to help improve the design of the groups about disability.

1. Can you tell me how you first heard about the group?
    - Can you tell me how many times you were invited? How did you receive this invitation?
    - Did you understand that you could join the group at any point? What could have helped to have made this more clear?

MRC/UVRI and LSHTM Uganda Research Unit

2. What do you think was the purpose of the group?

3. Some people in the groups started coming and then stopped, and others decided not to come at all. Can you tell me why you think some people might have decided to stop, or not to join at all? Can you tell me why you decided not to join the group?

Prompt: information, location, timing, frequency, eligibility

4. How much is your health, or difficulties accessing healthcare, an important issue in your life? Please explain your answer.
   - If yes – did you understand that this topic was the focus of the groups?

5. Can you tell me about other groups in the community that you have joined, whether of people with disabilities or not? What was the reason why you joined that group?

6. If we were to plan the groups again, what could we do differently to encourage you to join?

7. Can you tell me what might help others, or yourself, to attend these groups?
   - What kind of information might be useful?
   - Where could they be located?
   - What advice could you give the organisers of these groups on when the groups were held and how often?
   - How could others, or yourself, be made to feel more welcome and that they could be part of the groups?

8. Is there anything else that you would like to say about the group that we have not covered?

MRC/UVRI and LSHTM Uganda Research Unit

Qualitative Interview guide for people who have participated in PLA-D

Pilot-testing a participatory approach to improve access to healthcare for people with disabilities in Uganda

Objective: To gather information to help further design a participatory approach for people with disabilities to improve health

These questions should be used to guide discussion but do not have to be used in the sequence listed below. The interviewer should follow up on any additional issues that may arise and seem important in relation to the issues above.

Introduction

- Greet them and thank them for their time
- Identify yourself by name and organisation.
- Read out the information sheet. Remind them of confidentiality and anonymity. Check if they have any questions. Remind them that they are free to decline to answer any of the questions or stop the interview at any time.
- Record their consent/assent in the relevant form OR record verbal consent.
- Start recording

Notes: the following details must be recorded in field notes

Participant Code	
Interview date and time	
Interview location or mode (phone, video, in person)	
Interviewer	
Gender	
Age	
Profession	
General observations (anything which might impact how the interview is conducted)	

As we have discussed, we are gathering information to help improve the design of the groups about disability that you recently participated in.

1. Share with me your experience being a member of the group.
2. Can you tell me why you decided to join the group?

MRC/UVRI and LSHTM Uganda Research Unit

3. How many of the group sessions did you attend?
   Probe – if rarely – why?
   Probe – if frequently – why?

4. What do you think was the purpose of the group?

5. What did you think about the way your group was organised?

   Prompt – facilitation, regularity of meetings, location,

6. Are there any sessions that were particularly interesting or useful for you, and if so why?

7. What, if any, were the main benefits you perceived of the groups?

8. What actions were taken through or by your group? What do you think were the effect of your group was?

9. How/ in what ways were you supported by your group for example to attend, to participate?

10. Can you describe any changes that you have perceived in your life because of your group?

11. What interesting or important things have you learnt through participation in your group?

12. What, if any, were the main problems with your group?

13. Can you think of what any suggestions or feedback would be to someone who as thinking of establishing PLA-D groups?

14. If we were to plan the groups again, what could we do differently?

15. Is there anything else that you would like to say about your group that we have not covered?

For peer review only

MRC/UVRI and LSHTM Uganda Research Unit

Medical Research Council
Uganda Virus Research Institute
LONDON SCHOOL of HYGIENE &TROPICAL MEDICINE

LONDON SCHOOL of HYGIENE &TROPICAL MEDICINE

Observation of PLA-D group

The Missing Billion: Pilot-testing PLA-D groups to improve access to healthcare for people with disabilities in Uganda

Objective: To gather information to help further design a PLA-D group

Introduction

- Greet them and thank them for their time
- Identify yourself by name and organisation.
- Read out the information sheet. Remind them of confidentiality and anonymity. Check if they have any questions.
- Record their consent/assent in the relevant form OR record verbal consent.

Notes: the following details must be recorded in field notes

Group Code	
Observation date and time	
Observation location	
Observer name	
Participant gender (number men/women)	
General observations (anything which might impact how the interview is conducted)	

Observe the session and record your observations on the following page:

For peer review only

MRC/UVRI and LSHTM Uganda Research Unit

Facilitation group checklist	Activity demonstrated	Observed		Comment
		Yes	No	
Logistics	The group was held at the date, time and location that was expected			
	Number of facilitators who attended was the same as expected			
	Number of participants who attended were the same as expected			
	Group was conducted and ran according to schedule			
Environment created by facilitator	Room/location set up was in a circular pattern with all participants included			
	Room/location was accessible			
	The plan for the group session was discussed			
	Facilitators endeavoured to engage all the participants.			
	Facilitators used inclusive/accessible communication styles			
Response of participants	All participants contributed during the group, e.g. speaking to each other, speaking aloud in the group			
	Participants were distracted by competing interests, phone calls			
	There was a clear outcome to the session (e.g. barrier identified, plan agreed)			
	Participants appeared positive and engaged about the group			

For peer review only
